# Isolation of ‘Candidatus Ferrigenium straubiae’ – a microaerophilic Fe(II)-oxidizing bacterium and nitrate-reducing Fe(II)-oxidizer within the community of culture KS

**DOI:** 10.1099/ijsem.0.006949

**Published:** 2025-11-14

**Authors:** Stefanie Becker, Andreas Kappler

**Affiliations:** 1 Geomicrobiology, Department of Geosciences, University of Tübingen, Schnarrenbergstrasse 94–96, D-72076 Tübingen, Germany; 2 Cluster of Excellence: EXC 2124: Controlling Microbes to Fight Infection, Tübingen, Germany

**Keywords:** culture KS, *Ferrigenium straubiae*, *Gallionellaceae*, gradient tube, microaerophilic Fe(II) oxidation, nitrate-reducing Fe(II) oxidation

## Abstract

‘*Candidatus* Ferrigenium straubiae’ strain KS (KCTC 25982, DSM 118991) is a neutrophilic, Fe(II)-oxidizing bacterium representing up to 98% of the community in culture KS, the most extensively studied mixed culture known for autotrophic nitrate-reducing Fe(II) oxidation. The phylogeny and genome of this bacterium were previously analysed and identified as ‘*Candidatus* Ferrigenium straubiae’. In this study, we report the first-time successful isolation of ‘*Candidatus* Ferrigenium straubiae’ strain KS and its experimental physiological characterization. The bacterium was identified as a non-stalk-forming, rod-shaped and non-halophilic strain with a Gram-negative classification. We characterized its physiology when grown in agarose-stabilized Fe(II)-O_2_ gradient tubes where Fe(II) stemming from FeS functions as the electron donor and O_2_ as the electron acceptor. It showed growth at temperatures of 20–30 °C (optimal at 25°C) and at pH levels of 6.0–7.5 (optimal at pH 6.5–7.0). The doubling time at 20 °C and pH 6.5 was 16 h. We further optimized the gradient tubes for sustainable culture maintenance using modified Wolfe’s mineral medium (MWMM; 1 g l^−1^ NH_4_Cl) supplemented with 7-vitamin solution, SL-10 trace elements, selenite-tungstate solution and selenite-molybdate-nickel-copper-arsenic-vanadium solution (final concentrations of 10 µM Se, 10 µM Mo, 0.1 µM Ni, 0.1 µM Cu, 0.1 µM As and 5 nM V). We also evaluated several Fe(II) sources (with O_2_ as electron acceptor), as well as both inorganic and organic substrates for their influence on growth. Although a known member of the denitrifying community in culture KS, the isolated strain ‘*Candidatus* Ferrigenium straubiae’ KS exhibited exclusively microaerobic and autotrophic growth in agarose-stabilized Fe(II)-O_2_ gradients, utilizing Fe(II) from FeS as the electron donor.

**Impact Statement**

‘*Candidatus* Ferrigenium straubiae’ (‘*Ca*. F. straubiae’) is the main Fe(II)-oxidizer of the autotrophic enrichment culture KS, which is the most studied culture to investigate nitrate reduction and CO_2_ fixation coupled to Fe(II) oxidation. To gain a deeper understanding of the physiology and Fe(II) oxidation mechanism of culture KS, scientists have been trying to isolate this Fe(II)-oxidizer for decades. Here, we report how we finally isolated ‘*Ca*. F. straubiae’ and how this bacterium, which was previously unculturable as an isolate, can be maintained. Furthermore, we confirm the role of ‘*Ca*. F. straubiae’ as an autotrophic microaerophilic Fe(II)-oxidizer by physiological characterization. Using scanning electron microscopy, we vividly illustrate the distinct morphology of ‘*Ca*. F. straubiae’ as a non-stalk-forming, rod-shaped bacterium. We believe that our data, including a set of conditions under which ‘*Ca*. F. straubiae’ fails to grow, are a valuable piece of information. Furthermore, the isolation of ‘*Ca*. F. straubiae’ will be a milestone to further study and understand the bacterial interplay of autotrophic nitrate-reducing Fe(II)-oxidizing microbial communities.

## Data Summary

The authors confirm that all supporting data, code and protocols have been provided within the article or through supplementary data files.

## Introduction

*Ferrigenium*, *Ferriphaselus, Gallionella* and *Sideroxydans* are neutrophilic chemolithoautotrophic Fe(II)-oxidizing bacteria belonging to the same family, the *Gallionellaceae* [1,4]. While a few representatives [such as ‘*Candidatus* F. straubiae’ (‘*Ca*. F. straubiae’)] have been identified that couple Fe(II) oxidation to nitrate reduction, these bacteria are primarily known to oxidize Fe(II) under microoxic conditions [5,6]. The genus *Gallionella* was first described by Ehrenberg in 1836 and attracted scientific interest because of its ability to form helical structures called twisted stalks [7,8]. These helical structures are Fe(III)-mineral-rich extracellular organic filaments and are typically formed by microaerophilic Fe(II)-oxidizing bacteria. As stalks measure up to 200 µm, they were an ideal object for microscopic studies at the time of their discovery [8,9]. About a century later, the first pure culture of a species of the genus *Gallionella*, specifically *Gallionella ferruginea* Ehrenberg, was obtained by Hanert (1968), who was studying twisted stalk formation [10]. He used opposing Fe(II)-O_2_ gradient tubes containing FeS and air, a cultivation technique developed by Kucera and Wolf (1957), to specifically enrich microaerophilic Fe(II)-oxidizing bacteria [10,12]. However, the enrichment medium proposed by Kucera and Wolf was not sufficient to maintain their bacterial cultures at a certain point of transfers. To overcome this limitation, Kucera and Wolf reported that they had to add tap water which resulted in sustainable growth [11]. Wolfe (1958) stated that this effect was due to calcium which he added, instead of tap water, to refine the medium [13]. Although, with this adaptation, the medium was free of the use of tap water, Hanert applied FeS that was prepared using tap water to isolate *G. ferruginea* Ehrenberg [10]. This raises the question of whether there could be an overlooked importance in using tap water further than simply as a calcium source. Hanert *et al.* (2006) stated that differences in FeS are a critical factor for *Gallionella* cultivation, as improperly prepared batches of FeS inhibited growth. Although FeS was synthesized in distilled water, Hanert *et al.* advocated washing FeS once with tap water to avoid the formation of FeS hydrosols. Notably, this extra wash would also introduce tap-water-derived micronutrients to the final cultivation setup (via sorption to the FeS). Hanert *et al.* further explained that for growing microaerophilic Fe(II)-oxidizing bacteria, the following factors have to be considered: phosphate, ferrous and ferric iron, oxygen, pH and redox potential of the FeS [1]. Overall, maintenance and especially isolation of microaerophilic Fe(II)-oxidizing bacteria remain challenging and illustrate the thin line between cultivable and uncultivable bacteria.

Although several isolates of microaerophilic Fe(II)-oxidizers exist today, so far, no pure culture of chemolithoautotrophic nitrate-reducing Fe(II)-oxidizing (NRFeOx) bacteria that was shown to be transferred under autotrophic conditions for many generations has been obtained. However, our laboratory found that the enrichment cultures KS, BP, AG and HP were robust, autotrophic microbial communities under NRFeOx conditions [5,14,16]. The dominant bacteria of these four NRFeOx enrichment cultures belong to the family *Gallionellaceae* [6], just as it is the case for microaerophilic Fe(II)-oxidizing bacteria. The primary community members of cultures KS, BP and AG have been identified as the genus *Ferrigenium* and further classified as ‘*Ca*. F. straubiae’, ‘*Candidatus* Ferrigenium bremense’ and ‘*Candidatus* Ferrigenium altingense’, respectively [6]. Metagenomic analyses have provided critical insights into the metabolic potential of these bacteria, revealing genes encoding putative Fe(II) oxidases such as Cyc2 and/or MtoAB, as well as denitrification enzymes such as the nitrate reductase (Nar; *narGHI*), nitrite reductase (Nir; *nirK/S*) and/or nitric oxide reductase (Nor; *norBC*) [6,17]. In addition, the presence of the *rbcL* gene indicates the ability to fix carbon dioxide via the Calvin-Benson-Bassham cycle, supporting the hypothesis that these bacteria can perform autotrophic Fe(II) oxidation. However, direct isolation of the responsible autotrophic NRFeOx strains remains elusive, limiting our understanding of the mechanisms underlying this metabolism.

In the present study, we focus on the isolation of ‘*Ca*. F. straubiae’, the main Fe(II)-oxidizer of culture KS. This culture was isolated in Bremen (Germany) in the late 1990s and is the oldest and most extensively studied autotrophic NRFeOx enrichment culture [5,18]. A genomic analysis of culture KS was published by He *et al.* [17], with further phylogenetic, genomic and phenotypic analyses provided by Huang *et al.* [6]. Attempts to isolate ‘*Ca*. F. straubiae’ using Fe(II)/nitrate-containing medium have been unsuccessful, and the lack of a *nor* gene raises questions about its ability to perform nitrate-reducing Fe(II) oxidation independently of other community members. A meta-omic study by Huang *et al.* revealed ‘*Ca*. F. straubiae’ and *Rhodanobacter* species as interdependent key players for Fe(II) oxidation and nitrate reduction [19]. In their proposed model, *Rhodanobacter* detoxifies nitric oxide (NO), a cytotoxic intermediate, and acts as a mixotrophic (chemolithoheterotrophic) Fe(II)-oxidizer, relying on organic matter produced by ‘*Ca*. F. straubiae’. NO, being highly toxic, is typically detoxified by direct interaction of the NO-forming Nir and Nor (potentially as supercomplexes) [20,21], making it surprising that ‘*Ca*. F. straubiae’ appears to rely on passive diffusion of NO to community members. Despite these studies, the precise mechanisms of the autotrophic NRFeOx metabolic pathway remain unclear, underscoring the importance of isolating ‘*Ca*. F. straubiae’ and characterizing its growth, as a pure culture, under NRFeOx conditions. To date, no pure culture of ‘*Ca*. F. straubiae’ has been obtained, particularly not using Fe(II)/nitrate-containing medium. This raises the question of whether a critical growth factor has been overlooked, as has been the case for microaerophilic Fe(II)-oxidizing bacteria [1,4], or whether ‘*Ca*. F. straubiae’ is inherently dependent on its microbial community. Could there be a game-changing factor enabling its growth under these conditions? The present study is the first focused effort to isolate ‘*Ca*. F. straubiae’ since the original enrichment of culture KS. It highlights the enormous challenges of cultivating ‘*Ca*. F. straubiae’ and the complex interplay of factors that control its growth. By isolating and characterizing ‘*Ca*. F. straubiae’, we aim to deepen our understanding of NRFeOx bacteria and elucidate the intricate relationships between metabolic potential, environmental conditions and microbial physiology of these fascinating micro-organisms.

## Methods

### Source of micro-organism

Culture KS, originally cultured by Straub *et al.* [5], has been transferred under autotrophic conditions in our laboratory for more than 20 years. Typically, we transfer culture KS with 1% v/v inoculum on autotrophic, 22 mM bicarbonate-buffered, anoxic, unfiltered mineral medium containing 10 mM FeCl_2_ and 4 mM NaNO_3_ with a final pH of 6.9 to 7.2. The N_2_/CO_2_ ratio in the headspace was 90 : 10 v/v, and the incubation temperature was 28 °C without a light source and without shaking.

### Media and supplement preparation

The 22 mM and 30 mM bicarbonate-buffered mineral media were made after Hegler *et al.* [22], and anoxic media preparation was made after Widdel and Bak [23]. The 7-vitamin solution, SL-10 trace elements, selenite-tungstate solution (11 µM Na_2_SeO_3_ and 12 µM Na_2_WO_4_) were made as 1000× stock solutions as published by Widdel and Bak [23]. Kucera and Wolfe medium was made after Kucera and Wolfe [11]. Wolfe’s medium was made after Wolfe [13]. MWMM was made after Hanert *et al.* [1]. MWMM with 0.1 g l^−1^ NH_4_Cl was made after Lueder *et al.* [24]. Yeast Nitrogen Base (Invitrogen^™^) was prepared according to the manufacturer’s instructions. FeS and ferrihydrite synthesis are described in Lueder *et al.* [24]. The original laboratory’s detailed protocol for synthesizing FeS can be found in the supplemental information (Protocol SI-1, available in the online Supplementary Material).

### Cultivation for isolation and characterization of ‘*Ca*. F. straubiae’

#### Agar shakes

Agar shakes were prepared as described in Pfennig and Trüper [25] using 22 mM bicarbonate mineral medium containing 10 mM FeCl_2_ and 4 mM NaNO_3_.

#### Fe(II)-O_2_ gradient tubes for single-colony growth

The tubes were prepared with FeS and air as Fe^2+^ and O_2_ source, respectively. All variations are listed in Table 1 and were prepared according to the specified medium and referenced technique. The tubes were inoculated using 10% v/v culture KS, pre-grown in Fe(II)/nitrate-containing 22 mM bicarbonate-buffered mineral media.

**Table 1.** Conditions used to prepare Fe(II)-O_2_ gradient tubes aimed at promoting single-colony growth of ‘*Ca*. F. straubiae’. Conditions 2–10 were set up both with and without 5.2% formalin. Tubes with screw caps were prepared with either tightly or loosely closed caps. Additionally, conditions without formalin included test tubes with scratched glass surfaces. The composition of the medium is given as w/v except for tap water which is in v/v

No	FeS	Medium	Sealing	Ref. preparation/technique
1	Precipitate at bottom	Kucera and Wolfe medium (0.01% NH_4_Cl, 0.005% K_2_HPO_4_, 0.002% MgSO_4_ and 20% tap water)	Cotton plug	Kucera and Wolfe [11]
2	Precipitate at bottom	Wolfe’s medium (0.1% NH_4_Cl, 0.5% K_2_HPO_4_, 0.2% MgSO_4_, 0.01% CaCl_2_)	Butyl stopper	Modified after Kucera and Wolfe [11]
3	Precipitate at bottom	MWMM (0.1% NH_4_Cl, 0.005% K_2_HPO_4_, 0.2% MgSO_4_, 0.01% CaCl_2_)	Butyl stopper	Modified after Kucera and Wolfe [11]
4	Precipitate at bottom	30 mM bicarbonate-buffered mineral medium (0.03% NH_4_Cl, 0.6% KH_2_PO_4_, 0.05% MgSO_4_, 0.01% CaCl_2_)	Butyl stopper	Modified after Kucera and Wolfe [11]
5	1.5% agar slant	Wolfe’s medium (0.1% NH_4_Cl, 0.5% K_2_HPO_4_, 0.2% MgSO_4_, 0.01% CaCl_2_)	Screw cap	Wolfe *et al.* [13]
6	1.5% agar slant	MWMM (0.1% NH_4_Cl, 0.005% K_2_HPO_4_, 0.2% MgSO_4_, 0.01% CaCl_2_)	Screw cap	Modified after Wolfe *et al.* [13]
7	1.5% agar slant	30 mM bicarbonate-buffered mineral medium (0.03% NH_4_Cl, 0.6% KH_2_PO_4_, 0.05% MgSO_4_, 0.01% CaCl_2_)	Screw cap	Modified after Wolfe *et al.* [13]
8	1.5% agarose slant	Wolfe’s medium (0.1% NH_4_Cl, 0.5% K_2_HPO_4_, 0.2% MgSO_4_, 0.01% CaCl_2_)	Screw cap	Modified after Wolfe *et al.* [13]
9	1.5% agarose slant	MWMM (0.1% NH_4_Cl, 0.005% K_2_HPO_4_, 0.2% MgSO_4_, 0.01% CaCl_2_)	Screw cap	Modified after Wolfe *et al*. [13]
10	1.5% agarose slant	30 mM bicarbonate-buffered mineral medium (0.03% NH_4_Cl, 0.6% KH_2_PO_4_, 0.05% MgSO_4_, 0.01% CaCl_2_)	Screw cap	Modified after Wolfe *et al.* [13]
11	1.5% agar slant	Wolfe’s medium (0.1% NH_4_Cl, 0.5% K_2_HPO_4_, 0.2% MgSO_4_, 0.01% CaCl_2_)	Screw cap	Nunley and Krieg [47]

#### Agar plates

Sheep blood agar plates (Thermo Scientific^™^ Blood Agar) were ordered from Fisher Scientific. LB agar plates were prepared using Lennox LB Broth with agar (Sigma-Aldrich) according to the manufacturer’s instructions. The plates were incubated under oxic, microoxic and anoxic conditions.

Fe(II)/nitrate-containing agar plates were prepared using 22 mM buffered mineral medium (pH 7), 10 mM FeCl₂, 4 mM NaNO₃ and 1.5% w/v agar–agar Kobe I (Roth). These plates (10 cm in diameter) were poured while being flushed with a N₂/CO₂ gas mixture (90 : 10 v/v) in a glass beaker and were incubated anoxically.

Two-layer agar plates were prepared with a bottom layer (10 ml) containing either FeS slug 50% v/v or zero-valent iron (ZVI) 5% w/v in 1.5% w/v agar-agar Kobe I (Roth). While the bottom layer solidified, the plates were flushed with a N₂/CO₂ gas mixture (90 : 10 v/v) in a glass beaker. The top layer (15 ml) consisted of either 22 mM bicarbonate-buffered mineral medium with 4 mM NaNO₃ (pH 7) or 10 mM bicarbonate-buffered MWMM (pH 6.5). Plates containing nitrate were incubated anoxically, while plates with MWMM were incubated under microoxic conditions. All agar plates were incubated at 22 °C.

#### Incubation under an anoxic atmosphere (agar plates)

An anoxic atmosphere was achieved by use of a gas pack and specific plastic bags (Anaerocult^®^ C Mini, Merck) according to the manufacturer’s instructions.

#### Incubation under a microoxic atmosphere

A microoxic atmosphere of 6–10% atmospheric O_2_ concentration was achieved by the use of a gas pack (BD GasPak EZ Campy; Becton, Dickinson and Co., NJ) which was placed together with the cultures into an acrylic anoxic jar (Anaerocult^®^, Merck, Germany).

#### Dilution to extinction

We diluted to extinction a fresh and active KS culture (not older than 1 week, showing Fe(II) oxidation 2 days after inoculation), pre-grown anoxically in a 50 ml serum bottle at 28 °C. The culture was cultivated in 25 ml of 22 mM bicarbonate-buffered mineral medium containing 10 mM Fe(II) and 4 mM nitrate. For all dilutions, we transferred the sample anoxically using a syringe that had been pre-flushed with a N₂/CO₂ gas mixture (90 : 10 v/v). A 1 ml sample was withdrawn and diluted in 9 ml of the same medium (anoxic) in a test tube (10-fold dilution). A 10-fold serial dilution was further performed by transferring 1 ml from the lower fold into 9 ml of fresh medium (dilutions up to $10^{4}$-fold). Subsequently, a total of 19 test tubes were inoculated with 1.9 ml from the $10^{4}$-fold dilution (1.7 cells ml^−1^). The entire inoculum was collected once and evenly distributed across the 19 tubes, each prefilled with 9.9 ml of medium, resulting in a final $10^{6}$-fold dilution (0.017 cells ml^−1^). The test tubes were incubated at 28 °C and monitored weekly for ~3 months.

#### ZVI plates

ZVI plates were prepared as described in Laufer *et al.* [26] with slight modification by using 100 mg ZVI and 8 ml MWMM.

#### Agarose-stabilized Fe(II)-O_2_ gradient cultures

Agarose-stabilized Fe(II)-O_2_ gradient cultures were prepared as described in Emerson and Floyd [27] with the alteration that we adjust the pH before sterilization. A very detailed manual originating from our laboratory is included in the supplemental information (Protocol SI-2). For the top layer, we used Agarose Low Melt (ROTI^®^Garose, Roth), and for the bottom layer, peqGOLD Universal agarose (VWR). The medium used was 10 mM bicarbonate-buffered MWMM (pH 6.5), containing 7-vitamin solution, SL-10 trace elements and selenite-tungstate solution. The 8 ml gradient tubes, 50 ml serum bottles, 100 ml serum bottles and 500 ml glass bottles were incubated 1 day before oxygenation which was achieved by opening the cultures for ~1 min. The oxygenation step was combined with inoculation (1% v/v). The cultures were incubated at 22 °C (room temperature) in the dark. Any modifications are detailed in the text.

### Experimental setups

#### Physiology of isolated ‘*Ca*. F. straubiae’ growing with Fe(II) and O_2_

To determine the growth curve of ‘*Ca*. F. straubiae’ and to study the effects of Fe in abiotic and biotic agarose-stabilized Fe(II)-O_2_ gradient tubes, we conducted an experiment with sacrificial replicates. Each setup was prepared in triplicate. The gradient tubes were made with MWMM supplemented with 7-vitamin solution, SL-10 trace elements and selenite-tungstate solution and were incubated at 20 °C and pH 6.5. The biotic setups were inoculated with ~280 cells ml^−1^. The abiotic control setup was treated as the biotic setups; however, when opening the tubes for oxygenation, no inoculum was added. Fe(II) and total Fe concentrations were determined by the ferrozine assay, and growth was assessed by flow cytometry at 0, 1, 2, 3, 4, 5 and 7 days of incubation.

To determine the optimal pH, 10 mM bicarbonate-buffered MWMM was adjusted to various pH levels using organic buffers, including sodium citrate buffer (pH 4.5 and 5; 2 mM), MES (pH 5.5, 6 and 6.5; 10 mM) and HEPES (pH 7 and 7.5; 10 mM). To determine the optimum growth temperature, tubes were incubated at 5, 10, 15, 20, 25, 30 and 35 °C. For salinity tolerance, the top layer of the MWMM contained 25, 50, 75, 100, 200 and 300 mM NaCl. The tubes for the pH, temperature and salinity tests were inoculated with ~133 cells ml^−1^. Growth was assessed by flow cytometry 7 days after inoculation.

#### Physiology of microaerophilic Fe(II) oxidation in the presence of different Fe(II) sources and evaluation of alternative substrates in agarose-stabilized medium

Agarose-stabilized O₂ gradient tubes for testing alternative Fe(II) sources and substrates were prepared similarly to agarose-stabilized Fe(II)-O_2_ gradient tubes, but the FeS plug was replaced by an agarose plug composed of MWMM containing the respective substrate (substrates and concentrations tested are detailed in the main text). Cultures were inoculated with ~133 cells ml^−1^ and incubated at 20 °C. Growth was assessed by flow cytometry on days 8 and 9 after inoculation for Fe-containing setups and all other substrates, respectively. ZVI plates were prepared as described above, incubated for 8 days, and growth was assessed by fluorescence microscopy.

The experimental procedure for testing Fe(II) produced by Fe(III) photoreduction from a ferrihydrite plug followed the method described by Lueder *et al.* [24], with slight modifications, including the use of MWMM with 1 g l^−1^ NH₄Cl. Variations in setups and corresponding supplements are detailed in the main text. Cultures were inoculated with ~133 cells ml^−1^ and exposed to UV light (Plus lamp TVX20-ECO UVA 20W) inside a lab cabinet enclosed by an opaque black curtain at ~25 °C. Growth was assessed by flow cytometry 9 days after inoculation.

#### Assessment of denitrification by ‘*Ca*. F. straubiae’ in liquid media

Denitrification ability was tested in 10 ml of liquid medium (22 mM bicarbonate-buffered MWMM) inoculated with ~100 cells ml^−1^. Autotrophic conditions contained 10 mM FeCl₂ and either 4 mM NaNO₃ or 1.4 mM NaNO₂. Mixotrophic conditions contained 10 mM FeCl₂, 10 mM sodium acetate, 0.5 mM Fe(III)-citrate, 1 mM ascorbic acid and either 20 mM NaNO₃ or 10 mM NaNO₂. All four conditions were tested across a pH range of 5.5–8.2 (specifically 5.5, 6.0, 6.6, 7.0, 7.5 and 8.2). The pH 7.5 and 8.2 conditions were adjusted with an additional 25 mM HEPES buffer. Cultures were incubated at 20 °C, and growth was assessed by flow cytometry 10 days after inoculation.

#### Evaluation of different gradient tube protocols including the supplementation of tap water and the effect of FeS ageing

To evaluate growth effects resulting from ingredient modifications, we used a medium consisting of MWMM with 0.1 g l^−1^ NH₄Cl, 7-vitamin solution and SL-10 trace elements, but without selenite-tungstate solution. All modifications are detailed in the main text.

The evaluation of different FeS batches, FeS ageing, FeS washing and the use of tap water was conducted using MWMM with 0.1 g l^−1^ NH₄Cl, 7-vitamin solution, SL-10 trace elements and selenite-tungstate solution. Specific modifications, including supplementation and the use of tap water-medium (MWMM with 0.1 g l^−1^ NH₄Cl prepared with tap water instead of Millipore water), are described in the text. Cultures were incubated at 22 °C, and growth was assessed by flow cytometry 1 month after inoculation.

#### Modification of trace element solution for sustainable growth on gradient tubes

The optimization of the modified trace element solution was performed using MWMM (1 g l^−1^ NH₄Cl) and tap water-MWMM (1 g l^−1^ NH₄Cl), both supplemented with 7-vitamin solution, SL-10 trace elements and selenite-tungstate solution. For the first round of optimization, we used different combinations of 1 µM Se, 1 µM Mo, 0.5 µM Ni, 0.05 µM Cu, 0.05 µM As and 5 nM VO_3_: (1) non; (2) Se and Mo; (3) all; (4) all but no As; (5) all but no Cu; (6) all but no V; (7) Se, Mo and As; (8) Se, Mo and Ni; (9) Se, Mo and Cu; (10) Se, Mo and V. Cultures were incubated at 22 °C, and the appearance of the oxidation layer was followed visually daily for 1 week. Growth was assessed by fluorescence microscopy 1 month after inoculation. In the second round of optimization, we used different combinations of 10 µM Se, 10 µM Mo, 0.1 µM Ni, 0.1 µM Cu, 0.1 µM As and 5 nM VO_3_: (1) non; (2) Se and Mo; (3) Se, Mo, Cu and As; (4) all but no V; (5) all but no Ni; (6) all. Cultures were incubated at 22 °C, and the appearance of the oxidation layer was captured daily for 1 week. Growth was assessed by fluorescence microscopy 1 week after inoculation.

### Analytical methods

#### Determination of Fe(II) and total Fe by ferrozine assay

Total Fe and Fe(II) concentrations were quantified using a colorimetric ferrozine-based microplate assay in 96-well microplates [28,29]. For each measurement, 100 µl samples were taken from the carefully but well-mixed top layer of the gradient tube and immediately fixed in 900 µl of 1 M HCl, resulting in a 10-fold dilution. Fe(III) was reduced to Fe(II) by incubation with hydroxylammonium hydrochloride (HAHCl, 10% w/v in 1 M HCl) for 30 min at 22 °C. Twenty microlitres of the sample were added to 80 µl of 1 M HCl or HAHCl per well. One hundred microlitres of ferrozine reagent were added directly or 30 min later for total Fe measurement, respectively. Each sample was measured in triplicate. A calibration curve was generated using standards ranging from 0 to 1 mM ammonium Fe(II) sulphate in 1 M HCl.

The total Fe and Fe(II) concentrations were determined photometrically at 562 nm as ferrozine–Fe(II) complexes, following reaction with the ferrozine reagent [1 g l^−1^ 3-(2-pyridyl)-5,6-diphenyl-1,2,4-triazine-p,p′-disulphonic acid monosodium salt hydrate, 500 g l^−1^ ammonium acetate] for 5 min at 22 °C in the dark. Data were collected by a Multiskan GO Microplate Spectrophotometer (Thermo Fisher Scientific, Inc.). Total Fe(III) was calculated as the difference between total Fe and Fe(II).

#### Cell counts by flow cytometry

Samples were taken from a well-mixed top layer. Cells were counted by flow cytometry using a fluorescence dye (SYTO^™^ Green Fluorescent Nucleic Acid Stain SYTO 9, Invitrogen by Thermo Fisher Scientific, Inc.) and an Attune NxT connected to an auto sampler (Thermo Fisher Scientific, Inc.). The cell count was always conducted directly after sampling. To dissolve the iron precipitates, the cell culture (200 µl) was mixed gently with 100 mM EDAS-Fe(II) (200 µl) and oxalic acid [600 µl: 28 g l^−1^ ${\text{C}}_{\text{2}}{\text{H}}_{\text{2}}{\text{O}}_{\text{4}}\text{×}{\text{2NH}}_{\text{3}}$, 15 g l^−1^ ${\text{(COOH)}}_{\text{2}}\text{×}{\text{2H}}_{\text{2}}\text{O}$, pH 7.0] and incubated for 5 min at 70 °C followed by 25 min at room temperature. Subsequently, the samples were diluted according to the cell number, however, at least once using oxalic acid, resulting in a final cell concentration of 25–150 cells µl^−1^. The fluorescence dye was added (final concentration 1.25 µM) 12 min before measurement. A sample of the abiotic setup served as negative control, and the corresponding counts have been subtracted from the cell counts. Forty microlitres of samples were acquired at a flow rate of 12 µl min^−1^ using 488 nm blue laser excitation sources and BL1 530/30 channel. The side scatter, forward scatter and BL1 detector were set to 380, 480 and 420 V, respectively, and thresholds were set to 3×10^3^ fluorescence (BL1 530/30) intensity, 0.1×10^3^ side scatter intensity and 1×10^3^ forward scatter intensity. Side scatter, forward scatter and fluorescence were measured and reported as the height of the electronic pulse that comes off from the respective detector.

#### Identification of ‘*Ca*. F. straubiae’ by nearly full 16S ribosomal RNA gene sequencing using the Sanger method

Genomic DNA (gDNA) was extracted from 1 ml of the Fe(II)-oxidized zone (orange appearance) of a gradient tube culture. The sample was centrifuged for 1 min at 4,000 ***g*** and stored at −20 °C until further processing. gDNA extraction was performed using the DNeasy PowerSoil Pro Kit (Qiagen) according to the manufacturer’s instructions, with cell lysis carried out using a FastPrep^®^-24 (M.P. Biomedicals) at 4 m s^−1^ for 20 s.

PCR amplification of bacterial 16S rRNA gene fragments was performed using the general oligonucleotide primer pair GM38f [30]/1392r [31]. KAPA HiFi HotStart ReadyMix (Roche) was used for amplification according to the manufacturer’s protocol, and PCR was run on a Dyad PTC-220 Thermal Cycler (Bio-Rad). The PCR product was then purified using the Wizard SV Gel and PCR Clean-Up System (Promega Corporation).

The purified samples were sent for Sanger sequencing at Eurofins, following the instructions for their LightRun Tube sequencing service, using the following primers: GM38f, 341f [32], 807r [31] and 1392r. The sequencing reads (.ab1 files) were analysed by mapping them to the 16S rRNA gene of ‘*Ca*. F. straubiae’ (at IMG database, Taxon ID: 2565956535, Gene ID: 2566082739 and 2566083050) using Geneious Prime^®^ (Version 2019.2.3). The reads (FASTA file) have been deposited as prokaryotic 16S rRNA at NCBI and are available under the GenBank accession IDs: PV739805, PV739806, PV739807 and PV739808. Please note that the strain has two distinct 16S rRNA gene copies. The Sanger reads (.ab1 files) are visualized by mapping them to both 16S rRNA copies in the supplemental information (Fig. S3).

#### Whole-genome sequencing using the Illumina and Nanopore methods, assembly and analysis

We harvested a 1-year-old agarose-stabilized Fe(II)-O₂ gradient culture, which initially contained 20 ml of a FeS plug, a 50 ml top layer supplemented with selenite-molybdate-nickel-copper-arsenic-vanadium solution and a 30 ml headspace. One month before harvesting, we added 20 ml of fresh top layer to the oxidized zone and aerated the headspace to increase cell yield. Seventy millilitres of the top layer, along with the gel-like oxidized layer attached to the FeS plug, were collected in two 50 ml tubes and centrifuged at 10,000 ***g*** for 10 min at 20 °C. The transparent supernatant was discarded. To wash out the agarose, the remaining agarose-cell-mineral pellet was heated to 60 °C and mixed with 25 ml of DMSO (60 °C) per tube, followed by centrifugation at 6,000 ***g*** for 5 min at 20 °C. The cell-mineral pellets were transferred to two bead-beating tubes (DNeasy UltraClean Microbial Kit 50, Qiagen) without beads using a spatula. Any remaining pellet was washed off the tube wall with 5 ml of DMSO using a pipette. The DMSO prevented the cell-mineral mixture from adhering to the inside of the pipette tips. The samples were heated to 60 °C, followed by centrifugation at 20,238 ***g*** for 1 min at room temperature (table-top centrifuge, Eppendorf Centrifuge 5424) to further remove any remaining agarose and thereby reducing the pellet weight. After discarding the transparent supernatant, the beads were transferred back into the bead-beating tubes containing the cell-mineral pellet. gDNA was extracted using the DNeasy UltraClean Microbial Kit 50 (Qiagen), with the following alterations to the manufacturer’s instructions: (i) an alternative lysis method was used, including incubating at 70 °C for 10 min after adding Solution SL (step 3); (ii) at step 11, the DNA from the previously split sample was combined onto one MB Spin Column; (iii) to increase the yield, 50 µl of elution buffer at 60 °C was used at step 15, and the elution buffer was incubated on the column for 5 min before centrifugation at step 16. The sample was then stored at –20 °C until further processing.

Sample preparation and sequencing were carried out by the Genomics Core Facility of the Medical Faculty at Tübingen University, Institute for Medical Microbiology and Hygiene, University Hospital Tübingen (Germany). Libraries were prepared by 'Illumina DNA Prep, (M) Tagmentation' and 'ONT Native Ligation, LSK 114' for short-read and long-read sequencing, respectively. Illumina short-read sequencing was done by using a NovaSeq 6000 S1 Rgt Kit v1.5 (300 cyc) flow cell, and Nanopore long-read sequencing was done by using PromethION Flow Cell 10.4.1.

The Quantitative Biology Centre (QBiC) and Technology Platforms at the University of Tübingen (Germany) performed the data management and analyses. Quality control, trimming and assembly of the sequencing data were performed using nf-core/bacass v2.4.0 [33,34] (https://github.com/nf-core/bacass). The following tools were used for quality control and trimming: FastQC v0.12.1 [35] and FastP v0.23.4 [36] for short reads and NanoPlot v1.41.6 [37] and PoreChop v0.2.4 [38] for long reads. No obvious contamination was detected using Kraken2 v2.1.2 [39] or kmerfinder v3.0.2 [40]. Both the short-read and long-read data were assembled using Unicycler v0.5.0 [41] and polished using Medaka v1.4.3 [42].

QUAST [43] was used to compare the metagenome-derived genome of strain KS [19] (as used by Huang *et al.* [6]) and the genome derived from the pure culture of strain KS (this study). The QUAST results (reports and graphics) can be found in the supplemental information (Software output SI-1).

BUSCO v5.8.3 [44] was used to test for completeness and contamination. BUSCO estimated the completeness to 100% without contamination based on 116 bacterial markers, and 97.6% completeness with 1% duplicated markers based on 667 marker genes from *Nitrosomonas*. The results using both makers can be found in the supplemental information (Software output SI-2) .

The raw Illumina and Nanopore sequencing data have been deposited in the Sequence Read Archive at NCBI under the BioProject accession number PRJNA1271504, and the assembled genome has been deposited under BioProject PRJNA1271726 (GenBank accession ID: CP194331; IMG Taxon ID: 8132338324). Additionally, the two distinct full-length 16S rRNA gene copies (FASTA file) have been deposited as prokaryotic 16S rRNA at NCBI and are available under the GenBank accession IDs: PV740118 and PV740119.

#### Fluorescence microscopy

Fluorescence microscopy was performed using a Leica CTR5500 Microscope (Leica Microsystems GmbH) with the Leica Application Suite X software. Propidium iodide (PI) staining was conducted using the LIVE/DEAD^™^ BacLight^™^ Bacterial Viability Kit (Invitrogen, Thermo Fisher Scientific, Inc.) according to the manufacturer’s instructions. This kit contains not only PI but also SYTO 9, which stains both live and dead cells.

To assess growth, cells were stained with SYTOX^™^ Green (Invitrogen, Thermo Fisher Scientific, Inc.) after heat treatment at 70 °C for 5 min. SYTOX^™^ Green was diluted 1 : 1000 in Millipore water and stored at 4 °C. The stock solution was used to stain culture samples at a 1 : 1 dilution, resulting in a final staining concentration of 2.5 µM.

#### Scanning electron microscopy

The samples for scanning electron microscopy (SEM) were collected from a 1-month-old ‘*Ca*. F. straubiae’ culture grown on agarose-stabilized Fe(II)-O_2_ gradient serum bottle (100 ml). Cells were fixed in 2.5% glutaraldehyde for at least 24 h at 4 °C by adding 100 µl of 25% glutaraldehyde solution to 900 µl of culture under atmospheric conditions. After fixation, the sample was centrifuged (30 s at 2,348 ***g***), and 900 µl of supernatant was removed. The remaining sample was washed with 900 µl of ultrapure water and centrifuged again (30 s at 2,348 ***g***). This washing procedure was repeated four times. For sample preparation, 50 µl of sample was applied to poly-l-lysine-coated coverslips (coated with 35 µl of 0.01% poly-l-lysine solution, PLANO, Wetzlar, Item #18026) and dried at 50 °C for 2 h. The slide was placed in a 24-well plate, covered with the plate lid and left undisturbed for 30 min to allow the cells to settle. The sample was then dehydrated through a graded ethanol series (25, 50 and 75% for 15 min each, followed by 3×100% for 30 min). The sample was transferred to hexamethyldisilazane (HMDS) via a 1 : 1 ethanol/HMDS mixture for 30 min, followed by pure HMDS for another 30 min. Finally, 250 µl of fresh HMDS was added and allowed to evaporate overnight in a fume hood with the well-plate lid slightly open. After drying, cover the glass slide mounted on aluminium stubs with carbon tape (PLANO, Wetzlar, Item #G301 and G3347) and sputter-coated with ~8 nm platinum using a BAL-TEC SCD 005 sputter coater. SEM imaging was performed on a Zeiss Crossbeam 550L FIB-SEM at an acceleration voltage of 2 kV and a working distance of 4.0 mm, using the SESI detector for image acquisition.

## Results and discussion

### Isolation of ‘*Ca*. F. straubiae’

#### Challenges of growing single colonies and isolation by the dilution-to-extinction method

To isolate ‘*Ca*. F. straubiae’ from culture KS (pre-grown in Fe(II)/nitrate-containing 22 mM bicarbonate-buffered mineral media) as single colonies, we screened for optimal growth conditions using various techniques: Fe(II)-O_2_ gradient tubes, different kinds of agar plates and agar shakes using Fe(II)/nitrate-containing medium.

**Fig. 1.**
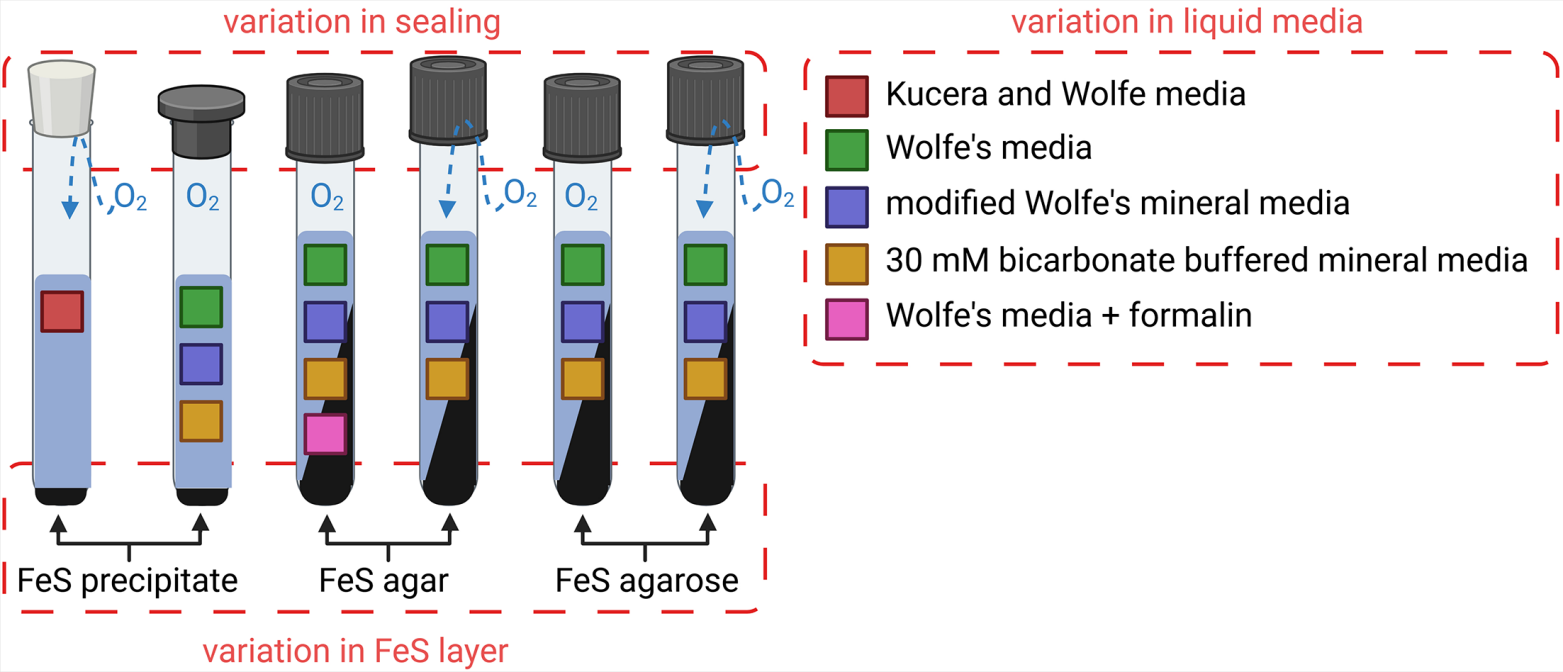
Variations of Fe(II)-O₂ gradient tubes aimed at promoting single-colony growth of ‘*Ca*. F. straubiae’. The setup using formalin was set up after Nunley and Krieg [47] and contained 5.2% formalin. Tubes with screw caps were prepared with either tightly or loosely closed caps. Additionally, conditions without formalin included test tubes with scratched glass surfaces.

Fe(II)-O_2_ gradient tubes rely on opposing gradients of Fe^2+^ dissolving and diffusing upwards from a bottom FeS layer and oxygen diffusing downwards from the air-filled headspace. Gradient tubes were originally developed for the cultivation of microaerophilic Fe(II)-oxidizing bacteria as single colonies attached to the glass wall and have been described in the literature for the specific enrichment of *Gallionella*. A great example was photographed by Verran *et al.* and Eggerichs *et al.* [45,46]. We adapted and modified different gradient tube protocols for our study. The main variation in these protocols lies in the preparation of the FeS layer, which is either a FeS precipitate at the bottom of a test tube [11] or a vertical slant along the tube wall [13]. The latter method was further modified by the addition of formalin [47]. A summary of these protocols and their modifications is shown in Fig. 1 and listed in Table 1.

Wolfe [13] applied screw caps to seal the gradient vessels. Here, we modified the screw-capped conditions (Table 1, conditions 5–10) by setting the tubes up twice, either tightly closed or loosely closed with a half turn. These two variations influence the oxygen concentration in the headspace of the vessel. To mitigate potential inhibitory effects of agar contaminants, the agar-FeS slant was modified by using agarose (Table 1, conditions 8–10). Formalin-free conditions were prepared twice, with one tube modified by glass wall scratches to increase surface area for microbial association and potential biofilm formation. All gradient tubes were incubated at room temperature.

We also tested agar plates prepared with different media and incubated under different oxygen conditions. Sheep agar and LB (Lennox and Luria) agar plates were incubated under different oxygen conditions: anoxic, microoxic and fully oxic atmospheres. In addition, Fe(II)/nitrate-containing agar plates were prepared under strictly anoxic conditions and incubated anoxically. Four specific conditions involved two-layer agar plates: the bottom layer contained either FeS or ZVI [15], while the top layer consisted of either 22 mM bicarbonate-buffered mineral medium with 4 mM nitrate (incubated anoxically) or MWMM (incubated microoxically). The agar shake method was also employed using 22 mM bicarbonate-buffered mineral medium with 10 mM Fe(II) and 4 mM nitrate under anoxic conditions at 28 °C. All setups were examined after 1 week and then monthly. Despite these efforts, none of the conditions and techniques tested, neither gradient tubes, agar plates nor agar shakes, succeeded in growing ‘*Ca*. F. straubiae’ colonies.

We used setups with both agar and agarose (a polysaccharide extracted from agar) because, although the supplier stated that agar is suitable for microbial use, it can form small organic molecules that inhibit growth when heat-sterilized. We expect this problem to be less prevalent with agarose. Another approach to overcoming the potential toxicity of agar is to wash the agar gel after autoclaving [MSc Yongjie Yu (AG Kappler), personal communication, 23 September 2025]. Yongjie Yu autoclaved the agar-water mixture and cut the solidified gel into small pieces. He then covered the gel pieces with Millipore water to dilute the small organic molecules by dialysis overnight. This process is repeated three times before a second heat sterilization, which makes the agar ready for use. Using this procedure, Yongjie Yu made 1.5% w/v agar plates with a trace of ZVI powder and a 22 mM bicarbonate-buffered mineral medium containing 10 mM Fe(II) and 4 mM nitrate under anoxic conditions. Using these plates, we obtained a slightly orange colony that we identified as ‘*Ca*. F. straubiae’ after sub-cultivation in agarose-stabilized Fe(II)-O₂ gradient tubes. However, we have not yet been able to reproduce these results, and no images were taken. Based on this recent finding, we suggest that further research using washed agar might lead to identifying a condition that supports the growth of colonies.

Since single-colony isolation methods failed at that point, the dilution-to-extinction method was used with liquid medium containing 10 mM Fe(II) and 4 mM nitrate (22 mM bicarbonate-buffered mineral medium) using a KS culture pre-grown on autotrophic NRFeOx condition. A total of 19 tubes were inoculated with 0.1 ml of a diluted KS preculture (1.7 cells ml^−1^) resulting in a 100-fold dilution. Of these 19 cultures, 3 isolates (Iso1–3) exhibited Fe(II) oxidation ~2 weeks after inoculation, while 4 more isolates (Iso4–7) exhibited Fe(II) oxidation after ~4 weeks.

The first subsequent cultivation (transfer) revealed different dilution tolerances among the isolates. Five isolates could not be diluted beyond 10-fold dilution, Iso5 was limited to 4-fold dilution and Iso3 could grow from dilution up to 100-fold in Fe(II)/nitrate-containing medium. Therefore, a 10% inoculum was used for successive transfers to Fe(II)/nitrate-containing medium, yet none of the isolates were able to maintain consistent Fe(II) oxidation capabilities over multiple transfers. Iso6 proved to be the most robust culture, maintaining autotrophic Fe(II) oxidation with nitrate for up to five transfers.

gDNA from the first transfer of Iso1–4 and Iso6 culture, as well as the original Iso5 and Iso7 culture, was subjected to Sanger sequencing of the ribosomal 16S rRNA gene. Sequencing confirmed the presence of ‘*Ca*. F. straubiae’ in all isolates, with purity indicated by clear, distinct sequencing reads. Due to its robustness, Iso6 was selected for further purity analysis, including heterotrophic growth testing on LB medium and contamination screening by fluorescence microscopy. Both tests confirmed the purity of the ‘*Ca*. F. straubiae’ culture. Exemplary Sanger reads and micrographs are shown in the supplemental information (Figs S3 and S4, respectively).

Although this clearly showed that the dilution-to-extinction method was successful using Fe(II)/nitrate-containing medium, Iso6 could not be maintained autotrophically on Fe(II)/nitrate-containing liquid medium. To overcome this limitation, we used Iso6 as inoculum, screening for an alternative substrate that could support the continuous growth and maintenance of ‘*Ca*. F. straubiae’.

#### Agarose-stabilized Fe(II)-O₂ gradients – the ultimate conditions to successfully grow ‘*Ca*. F. straubiae**’**

**Fig. 2.**
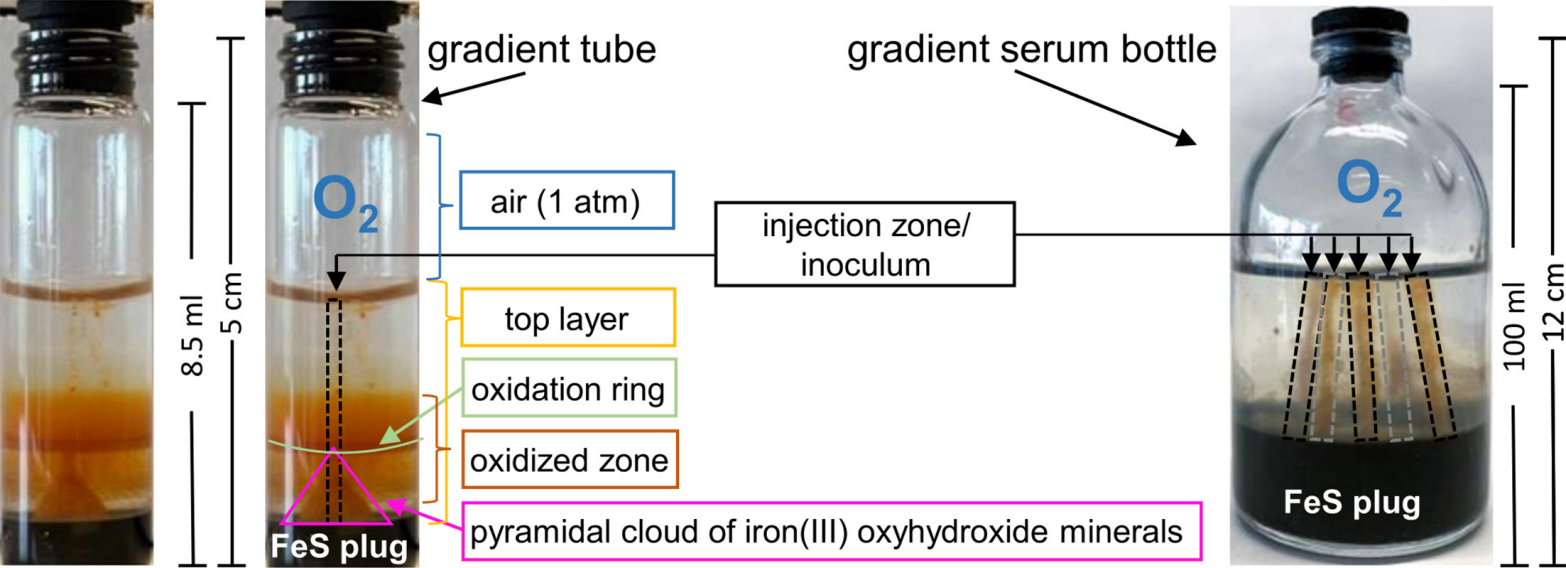
Setup and terminology of agarose-stabilized Fe(II)-O_2_ gradient cultures in a tube (8.5 ml) and in a serum bottle (100 ml). The top layer of 10 mM bicarbonate-buffered medium (pH 6.5) contains 0.15% w/v agarose (yellow), and the bottom layer (black), called the FeS plug, contains 1% w/v agarose, 50% v/v FeS slug and 50% v/v medium. The gradient tube contains a 760 µl FeS plug, 3.875 ml top layer and 3.865 ml headspace. The gradient serum bottle contains a 20 ml FeS plug, 50 ml top layer and 30 ml headspace. The gradient tube has been incubated for ~1 month before the photograph has been taken; the gradient serum bottle has been imaged just after inoculation. While gradient tubes are inoculated with 1% v/v of the top layer in a continuous line starting over the FeS plug to the surface of the top layer, this 1% inoculum was divided into 5 inoculation points, resulting in 5 equal lines in a serum bottle (black and grey dashed boxes). The pyramidal cloud of iron(III) oxyhydroxide minerals visible in the gradient tube indicates bacterial activity and is slightly visible under optimal growth conditions about 2 days after inoculation (pink triangle) and becomes more dominant during the first week of incubation.

To identify conditions that support the maintenance of the pure ‘*Ca*. F. straubiae’ culture, we designed a multi-condition screen that included various combinations of media, substrates and amino acid concentrations (details in supplemental information, Text SI-1). Liquid cultures were inoculated by using 10% of Iso6 pre-grown on Fe(II)/nitrate-containing medium (fifth transfer after isolation). However, it was without success. In parallel, Iso6 was transferred to agarose-stabilized Fe(II)-O_2_ gradient tubes (hereafter referred to as gradient tubes) (Fig. 2). In these gradient tubes, we found ‘*Ca*. F. straubiae’ to grow very well.

Growth was verified using fluorescence microscopy, which showed dense cell populations in association with iron(III) oxyhydroxide minerals (Fig. 5 of section 'Microscopic analysis of ‘*Ca*. F. straubiae’ growing with Fe(II) and O_2_'). Purity testing by Sanger sequencing and microscopy revealed no impurities (Fig. S3 and Fig. S4). Since ‘*Ca*. F. straubiae’ grew exclusively in these microoxic gradient tubes, microaerophilic, Fe(II)-oxidizing growth was further characterized and established as the primary method for culture maintenance. However, it is noteworthy that ‘*Ca*. F. straubiae’ does not recover well when diluted beyond 10^−^² during transfer to a fresh gradient tube.

**Fig. 3.**
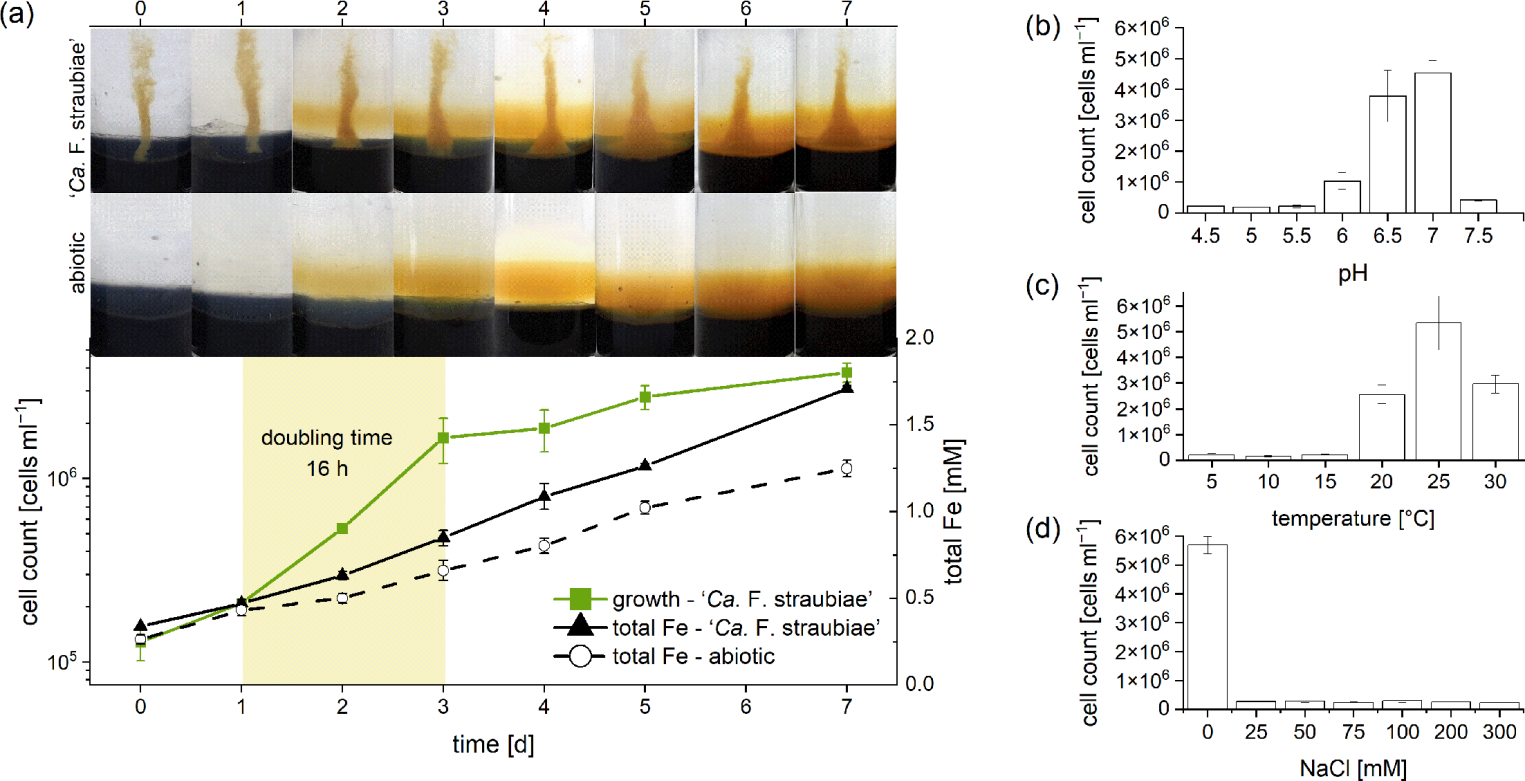
(**a**) Growth behaviour of ‘*Ca*. F. straubiae’ in agarose-stabilized Fe(II)-O_2_ gradient tubes at 20 °C and pH 6.5. For this analysis, we utilized sacrificial replicate setups, with each time point representing biological triplicates. A photograph of one harvested replicate is shown above the corresponding time point in the growth curve and iron concentration plot. (b–d) Growth preferences of ‘*Ca*. F. straubiae’ under varying conditions: pH (**b**), temperature (**c**) and salinity (**d**). The column bars indicate the cell numbers in 7-day-old cultures grown at 20 °C. Error bars correspond to biological triplicates.

Gradient tubes are commonly used to enrich microaerophilic Fe(II)-oxidizing bacteria, which typically form species-specific oxidation layers or clouds at characteristic heights. In the case of ‘*Ca*. F. straubiae’, we commonly observed a pyramidal cloud of iron(III) oxyhydroxide minerals (Fig. 2). When ‘*Ca*. F. straubiae’ was grown at 20 °C and pH 6.5 in gradient tubes, this pyramidal cloud of iron(III) oxyhydroxide minerals appeared 2 days after inoculation, which was clearly distinguishable from the iron minerals which formed abiotically in gradient tubes without microbial inoculation (Fig. 3a of section 'Physiology of the isolated ‘*Ca*. F. straubiae’ growing with Fe(II) and O_2_'). Generally, this pyramidal cloud was observed for fast-growing cultures younger than 1 month. When the cultures grew slowly or when the cultures were some months old, this pyramid could not be identified anymore, although the cell population increased (examples are the first two cultures shown in Fig. 10 of section 'Effect of FeS ageing on the growth of ‘*Ca*. F. straubiae’ in agarose-stabilized Fe(II)-O_2_ gradient tubes').

‘*Ca*. F. straubiae’ cultures were mainly grown in 8.5 ml tubes. However, ‘*Ca*. F. straubiae’ could also be grown in up-scaled gradient cultures using vessels such as 50 ml (Fig. 6 of section '‘*Ca*. F. straubiae’ impedes abiotic iron oxidation in agarose-stabilized Fe(II)-O_2_ gradients'), 100 ml serum bottles (Fig. 2) and 500 ml bottles.

As we included the growth medium for shipment of the bacterial strain to the Korean Collection for Type Cultures, we prepared the bottom and top layers separately. This enabled the curator to add the gelatinized top layer onto the bottom layers via pipetting without heating it, 1 day before use. This method was most convenient as it allowed us to add all the supplements to the top layer, and the curator did not have to sterilize anything. We tested this modified protocol successfully with duplicates in our laboratory, and the curator successfully sub-cultured ‘*Ca*. F. straubiae’ strain KS on the provided medium. This indicates that the method likely works with universal agarose, which has a higher melting point than low-melt agarose and is commonly used for molecular biological work. Initially, low-melt agarose was chosen to allow more time for adjusting the pH of the medium after sterilization (Dr. D. Emerson, personal communication, 23 September 2025). However, although we adjust the pH before sterilization, we have never considered using universal agarose for the top layer. Since even gelatinized medium can be used for sub-culturing, universal agarose is likely an option for laboratories that do not have low-melt agarose in stock.

### Long-term preservation of ‘*Ca*. F. straubiae’ strain KS

The DSMZ (Leibniz Institute – German Collection of Microorganisms and Cell Cultures GmbH) successfully achieved long-term preservation using ‘*Ca*. F. straubiae’ strain KS cultures grown in agarose-stabilized Fe(II)-O₂ gradient tubes. The cryo-stocks were stored in liquid nitrogen with 5% v/v DMSO as the cryoprotectant [Dr. S. Spring (curator at DSMZ), personal communication, 31 March 2025]. Our laboratory verified cell growth after receiving a sample of ‘*Ca*. F. straubiae’ strain KS from the DSMZ. However, our attempts to prepare cryo-stocks with either 20% v/v glycerol or 50% sugar-containing cryo-solution (20% w/v glycerol and 0.05 g ml^−1^ sucrose in H₂O) followed by flash freezing in liquid nitrogen and storage at −80 °C were unsuccessful. Instead, we maintained an active culture in our laboratory with annual transfers using gradient tubes, for more than 6 years so far.

### Whole-genome sequencing of the pure culture of ‘*Ca*. F. straubiae’ strain KS

The assembled genome of the isolated strain of ‘*Ca*. straubiae’ strain KS (GenBank accession ID: CP194331) comprises a single sequence of 2,666,173 bp containing around 2,500 genes, including two 16S rRNA genes (GenBank accession IDs: PV740118 and PV740119). The genome was also added to JGI GOLD (Genomes OnLine Database), and the sequence data were submitted to IMG (Integrated Microbial Genomes) for annotation (IMG Taxon ID: 8132338324; 16S rRNA genes: 8132340740 and 8132340651) [48,49].

Based on 116 bacterial marker genes, the genome is 100% complete with no duplication. Based on 667 marker genes from *Nitrosomonas*, the genome is 97.6% complete with 1% duplication (BUSCO output files are in supplemental information, Software output SI-2). To verify the accuracy of the sequence, we compared it with the metagenome-derived genome of ‘*Ca*. F. straubiae’ strain KS (GenBank ID: GCA_019449655.1), discovering that 99.998% of sequences were identical [6,43]. The average nucleotide identity was found to be 99.9778%, and the G+C content was found to be almost identical (60.24 mol% versus 60.26 mol%). These results suggest that the two sequences are highly similar and correspond to the same strain (QUAST report file is in supplemental information, Software output SI-1). Please note that sequencing errors due to library preparation, sequencing technology or assembly errors due to imperfect software are inevitable. This is especially true for metagenome-assembled genomes because contamination causes additional small errors. Considering that the ‘*Candidatus* F. straubiae’ strain KS was proposed by Huang *et al.* (2022) based on the metagenome derived from the enrichment culture KS (98% ‘*Ca*. F. straubiae’) and that the pure bacterium strain KS described here is isolated from this culture KS and has a nearly identical genome, we conclude that they are the same strain [6].

We refer to the study of Huang *et al.* (2022) proposing the new taxon – ‘*Candidatus* Ferrigenium straubiae’ for general genomic information, analysis of genomic differences between the new taxon and closely related type strains, phylogenetic analysis and further genomic analysis to differentiate between the closely related new taxa ‘*Candidatus* Ferrigenium bremense’ and ‘*Candidatus* Ferrigenium altingense’ [6].

### Physiological characterization of ‘*Ca*. F. straubiae’

#### Physiology of the isolated ‘*Ca*. F. straubiae’ growing with Fe(II) and O_2_

‘*Ca*. F. straubiae’ was able to grow with Fe(II) and O_2_ in agarose-stabilized Fe(II)-O_2_ gradient tubes between 20 and 30°C and between pH 6.0 and 7.5. Optimal growth was observed at 25 °C and pH 6.5–7.0 (Fig. 3b, c). ‘*Ca*. F. straubiae’ showed very little tolerance to salinity, i.e. 25 mM NaCl was already inhibitory (Fig. 3d).

These optimal growth conditions are within the same range as those reported for related bacterial genera (Table 2). It is important to note that buffer systems can affect iron chemistry, which might influence the bioavailability of Fe(II). Furthermore, organic buffers can be toxic to bacteria. However, this should not be the case when using Good buffers [50,51]. Since the chosen buffer systems are Good buffers or were chosen for similar studies, we consider them compatible for testing the growth of ‘*Ca*. F. straubiae’ [4,52]. The pH range (5.5–7.5) in which we observed optimal growth (pH 6.5 and 7.0) was covered by MES and HEPES buffers. Comparing the conditions with MES buffer at pH 6.5 (10 mM) and HEPES buffer at pH 7 (10 mM) to the condition with no organic buffer reveals that the buffer system had no effect on the pH test results. After 7 days, the cell numbers for the conditions with no organic buffer (pH 6.5), MES buffer (pH 6.5) and HEPES buffer (pH 7.0) were ${3.79\times 10}^{6}(\pm 11.04\%)$, ${3.80\times 10}^{6}(\pm 21.94\%)$ and ${4.53\times 10}^{6}(\pm 9.06\%)$ cells ml^−1^, respectively.

**Table 2.** A phenotypic and genotypic comparison of ‘*Candidatus* Ferrigenium straubiae’ with closely related strains from different species and genera within the *Gallionellaceae* family. The table was modified after Huang *et al.* (2022) [6]

Characteristics	‘*Candidatus* Ferrigenium straubiae’ KS	‘*Candidatus* Ferrigenium bremense’ BP	‘*Candidatus* Ferrigenium altingense’ AG	*Ferrigenium kumadai* An22	*Sideroxydans lithotrophicus* ES-1	*Gallionella* capsiferriformans ES-2
Isolation source	Sediment	Sediment	Aquifer	Rice paddy soil	Groundwater	Groundwater
Geographic location	Bremen, Germany	Bremen, Germany	Altingen, Germany	Anjo, Japan	MI, USA	MI, USA
Cell morphology	Curved rod	Curved rod	Curved rod	Curved rod	Helical rod	Curved rod
Cell size (L×W, in μm)	0.8–2.2×0.2–0.7	1.1–2.3×0.2–0.6	0.5–1.8×0.2–0.6	0.9–2.0×0.2–0.4	0.3 diameter	0.7 diameter
Stalk formation	N/O	N/O	N/O	−	−	−
Motile/flagella	N/O	N/O	N/O	+	+	+
Doubling time (h)	16.0	46.1	35.0	6.2	8.0	12.5
Growth temperature (°C)	20–30	28*	25*	12–37	10–35	4–30
Growth pH	6.0–7.5	6.9–7.2*	6.0–7.0*	5.2–6.8	5.5–7.0	5.5–7.0
NaCl (g l^−1^)	Below 1.46	NR	NR	Below 1.46	NR	NR
Carbon fixation	+	+	+	+	+	+
Community member in NRFeOx enrichment culture	+	+	+	NR	NR	NR
Microaerophilic Fe(II)-oxidizer	+	N/O	N/O	+	+	+
Genome: GenBank accession; IMG Taxon ID	CP194331; 8132338324	JAGRPI00000000; 2831290873	JAHRYS000000000; 2860363623	AP019536; 2927562029	CP001965; 646564569	CP002159; 648028028
Genome size (kbp)†	2,666.173	2,408.093	2,180.025	2,572.603	3,003.656	3,162.471
G+C content (mol%)†	60.24	59.05	57.97	60.60	57.54	52.75
16S rRNA gene copies	2	1	1	2	2	3
Completeness (%)†	99.97	94.08	81.34	Complete	Complete	Complete
Reference	Straub *et al.* (1996); He *et al.* (2016); Tominski *et al.* (2018); Huang *et al.* (2021); Huang *et al.* (2022); this study [5,6, 17, 19, 75]	Huang *et al.* (2021); Huang *et al.* (2022) [6,76]	Jakus *et al.* (2021); Huang *et al.* (2021); Huang *et al.* (2022) [6,15, 77]	Khalifa *et al.* (2018) [4]	Emerson *et al.* (2013); Emerson and Moyer (1997) [2,78]	Emerson *et al.* (2013); Emerson and Moyer (1997) [2,78]

*Temperature/pH of the whole enrichment culture BP and AG, respectively.

†Data correspond to IMG pipeline statistics.

N/O, not observed; NR, not reported; NRFeOx, nitrate-reducing Fe(II)-oxidizing.

When ‘*Ca*. F. straubiae’ was grown at 20 °C and pH 6.5, the doubling time was 16 h. We chose 20 °C because we believe it is environmentally relevant. The average monthly temperature in Bremen (Germany), where the strain originates from, does not exceed 20 °C [53]. This is lower than the optimum growth temperature of 25 °C. We also monitored Fe(II) oxidation under the same conditions. After a 1-day lag phase, Fe(II) oxidation [as measured by total iron accumulation over time in the upper layer] of the gradient tube inoculated with ‘*Ca*. F. straubiae’ was significantly higher than that in the abiotic control (Fig. 3a). Very similar data for total Fe and growth were published by Kato *et al.* [52] for the microaerophilic Fe(II)-oxidizing bacteria strain OYT1 growing in gradient tubes, which suggest that Fe(II) was used as substrate by ‘*Ca. F*. straubiae’ with O_2_ as an electron acceptor.

The Fe(II) and Fe(III) data (Figs S1 Fig. S2) clearly indicated drastic geochemical changes within 3 days after the gradient tubes were oxygenated by opening the anoxic headspace exposing it to air at atmospheric pressure and room temperature. Fe(II) and Fe(III) data, as well as the change of growth rates, are discussed in the supplemental information (Text SI-2-5). Cell count data shown in Fig. 3 confirm the growth of ‘*Ca*. F. straubiae’ in Fe(II)-O₂ gradients, and the higher total Fe data compared to the abiotic setup identify the strain as Fe(II)-oxidizer.

#### Microscopic analysis of ‘*Ca*. F. straubiae’ growing with Fe(II) and O_2_

**Fig. 4.**
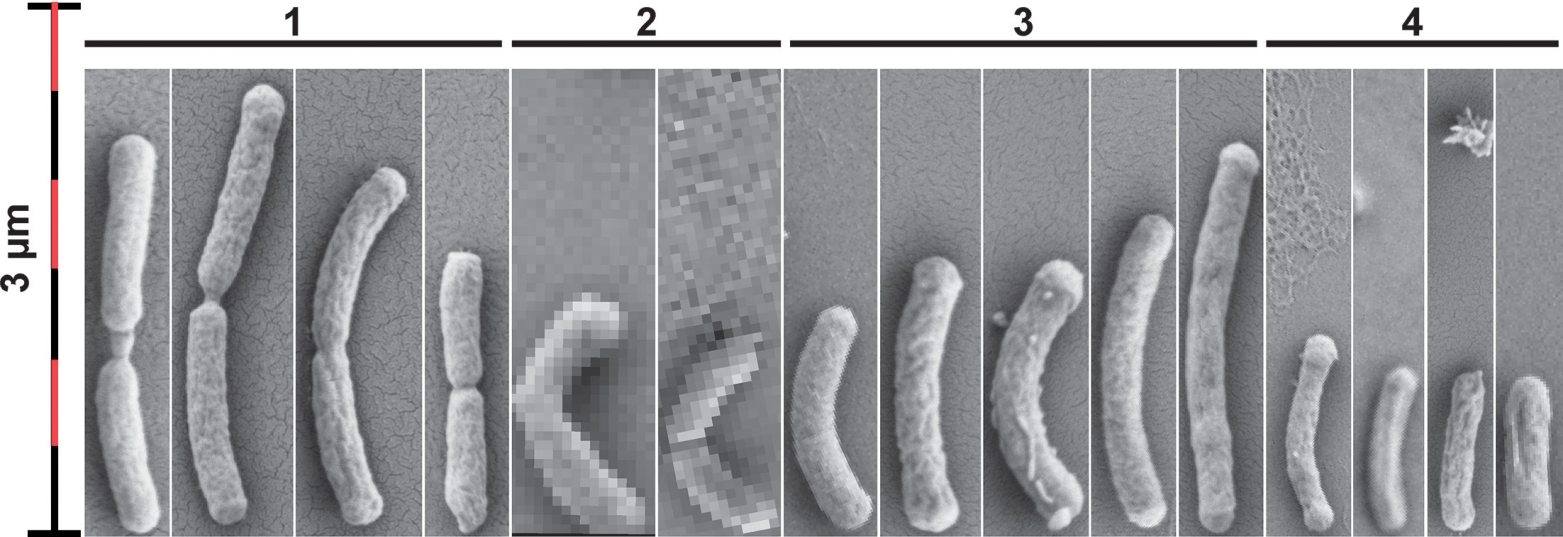
Scanning electron microscopy images showing the morphological variations of ‘*Ca*. F. straubiae’. The cells are grouped into four similar shapes and originate from independent micrographs taken at different resolutions. For comparison, a uniform scale is used across all images, resulting in some micrographs appearing at lower resolution than others. Group 1 shows cells that might be just at the end of division resulting in two distinguishable cells. Group 2 shows two cells connected, appearing like a boomerang. Group 3 shows individual, rather big cells compared to group 4, showing smaller individual cells. The samples analysed were obtained from an agarose-stabilized Fe(II)-O_2_ gradient culture.

‘*Ca*. F. straubiae’ was grown in agarose-stabilized Fe(II)-O_2_ gradient tubes and bottles. In the microscope, ‘*Ca*. F. straubiae’ cells appear as slightly curved rods (∼0.8 to 2 µm long), and the smaller ones may not look curved (Fig. 4, groups 3 and 4). The bacterial cells are often found as two associated rods (Fig. 4, group 1), the very view of which can be found forming a smaller angle, looking like a boomerang (Fig. 4, group 2). Twisted stalks were not observed under any of the conditions tested in this study.

**Fig. 5.**
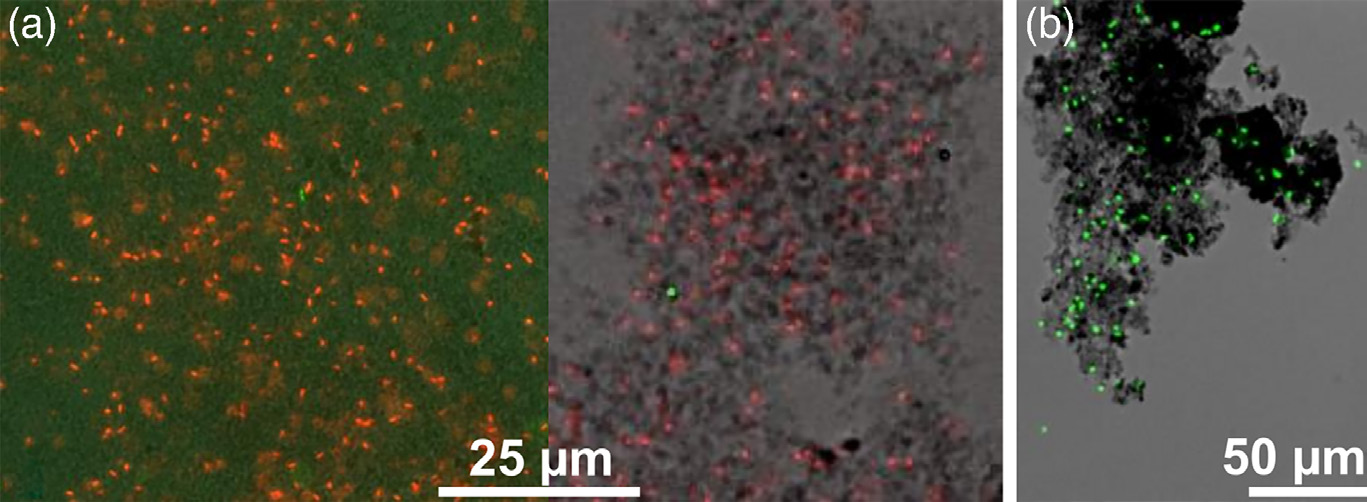
Fluorescence micrograph showing (**a**) PI-stained (red) and (**b**) SYTOX^™^ Green-stained ‘*Ca*. F. straubiae’ cells associated with Fe(III) minerals. The cells stained with SYTOX^™^ Green got a heat treatment of 5 min at 70 °C. Cells grew in an agarose-stabilized Fe(II)-O_2_ gradient in a 100 ml serum bottle and steam from the oxidized zone just above the FeS plug. (Fig. S4 shows additional micrographs of SYTOX^™^ Green-stained cells taken at different magnifications.)

Cell growth took place just above the FeS plug, where the cells grew very dense and were associated with iron minerals (Fig. 5). The micrograph shows cells stained with PI (dead cell stain, Fig. 5a). The cells also stain well with SYTO 9 and appear very bright with SYTOX^™^ Green (Fig. 5b). Additionally, staining intensity improves after heat treatment, which can be achieved either by brief microwaving or a 5 min incubation at 70 °C.

#### ‘*Ca*. F. straubiae’ impedes abiotic iron oxidation in agarose-stabilized Fe(II)-O_2_ gradients

**Fig. 6.**
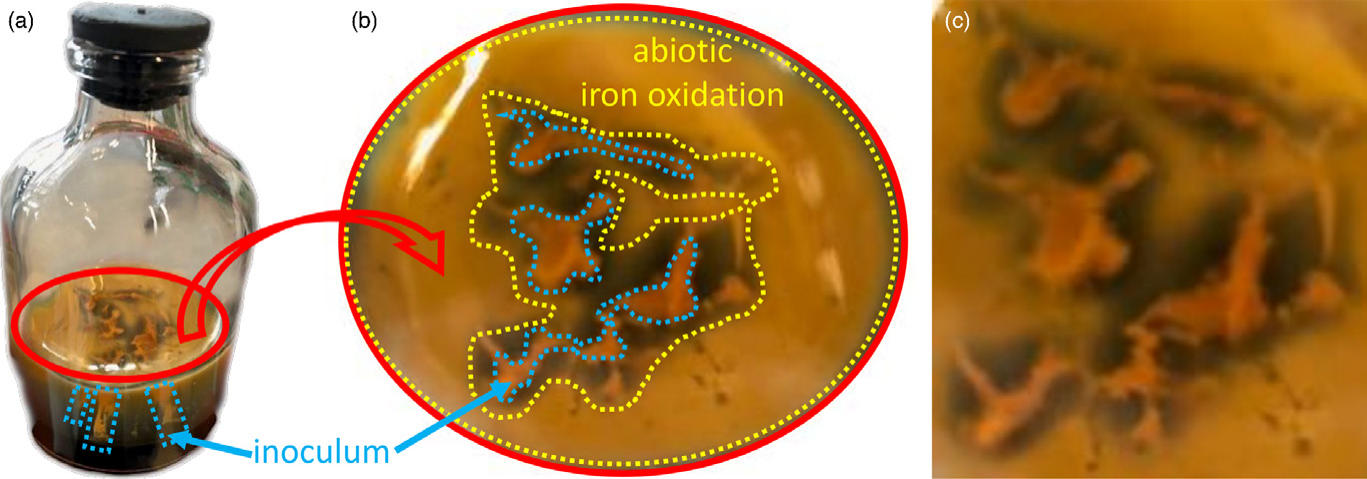
Non-oxidized areas around ‘*Ca. F*. straubiae’ cultures observed in agarose-stabilized Fe(II)-O_2_ gradient serum bottles. (**a**) Culture was inoculated in 5 locations (total 1% v/v of the top layer) into a 50 ml serum bottle containing a 10 ml FeS plug and a 25 ml top layer, and the bottle was incubated for 1 month at room temperature. (**b**) Top view of the culture surface with the abiotically oxidized area and inoculum marked. The non-oxidized areas appear black due to the transparency of the medium, allowing the FeS plug at the bottom of the bottle to show through. The area between the glass wall and the yellow dashed line, as well as the central area enclosed by the yellow dashed line, represents zones abiotically oxidized by oxygen from the headspace. These zones appear orange due to Fe(III) minerals. The inoculum, indicated by the blue dashed lines, contains Fe(III) minerals from the preculture and therefore appears orange as well. (**c**) For clarity, the surface is shown without any markings.

When ‘*Ca*. F. straubiae’ was inoculated into gradient serum bottles, we observed that abiotic oxidation caused by oxygen from the headspace was not present in the areas around the injected bacterial culture. An example is shown in Fig. 6, which shows a 50 ml serum bottle containing a 10 ml FeS plug and a 25 ml top layer (Fig. 6a). When inoculating gradient cultures, the inoculum is evenly distributed in a line starting just above the FeS plug and extending upward to the surface of the top layer. For serum bottles, the inoculum totalling 1% v/v of the top layer is injected in multiple locations, resulting in approximately five lines (clearly visible in Fig. 2b and less so in Fig. 6a). The preculture used for inoculation was harvested from the Fe(II)-oxidized zone of a gradient tube and contained orange Fe(III) minerals. Consequently, the lines where the inoculum was placed appear orange immediately after inoculation due to the presence of Fe(III) minerals. Abiotic Fe(II) oxidation caused by oxygen from the headspace primarily affected the surface of the top layer. This abiotic oxidation also appeared as orange Fe(III) minerals. The non-oxidized areas [without orange Fe(III) minerals] were only visible when looking at the surface of the top layer, as shown in Fig. 6b and c, and were consistently located around the injection sites. It appears black because the medium is transparent when not oxidized, and the black FeS plug at the bottom can be seen.

This observation suggests that ‘*Ca*. F. straubiae’ secretes metabolites (collectively known as the exometabolome) that significantly impede abiotic iron oxidation by O_2_. A similar effect was evidenced by Baker *et al.* [54], who examined the exometabolome of the deep-sea Fe(II)-oxidizing bacterium *Ghiorsea bivora* TAG-1. Their study demonstrated that *G. bivora* TAG-1 chemically alters its environment to limit abiotic interactions between Fe(II) and O_2_. If the exometabolome of ‘*Ca*. F. straubiae’ has a similar chemistry, it could diffuse into the medium surrounding the injection sites, thereby preventing the enclosed area from undergoing abiotic Fe(II) oxidation and explaining the observed non-oxidized zones.

#### Physiology of microaerophilic Fe(II) oxidation by ‘*Ca*. F. straubiae’ in the presence of different Fe(II) sources

Growth assays were performed for ‘*Ca*. F. straubiae’ in agarose-stabilized, Fe(II)-containing media using FeSO₄ (1 and 5 mM), FeCl₂ (1 and 5 mM) or Fe⁰ (ZVI; 20% w/v) in the Fe(II)-containing plug. These tests were performed in triplicate, both as a closed system with an opposing oxygen gradient and in open systems (loosely closed with aluminium foil) which were incubated in a microoxic atmosphere. None of these Fe(II) sources and oxygen variations supported the growth of ‘*Ca*. F. straubiae’. We further tested ZVI as 'ZVI plates' using liquid medium (MWMM) under a microoxic atmosphere, using the same pre-culture inoculum as for the iron(II) source assays, but no growth was observed. Occasionally, population increases were detected using ZVI plates (data not shown), but these results were inconsistent and not reproducible. Maintenance of cultures on ZVI plates was not possible, and dilutions of the inoculum for ZVI plates beyond 100-fold consistently failed completely. Considering that ‘*Ca*. F. straubiae’ oxidizes Fe(II) and cell proliferation is observed (Fig. 3), it is surprising that other Fe(II) sources did not support growth. The Fe(II) concentration in the gradient tubes does not exceed 0.4 mM (Fig. S1A), and the concentration of dissolved Fe²^+^ in a 22 mM bicarbonate medium with 10 mM Fe(II) is ~2 mM [55]. The latter is the condition in which culture KS (98% ‘*Ca*. F. straubiae’ [6]) is commonly cultured. This indicates that the tested Fe(II) concentration was likely not too high; however, further research is needed to determine if there is an Fe(II) toxicity threshold. Fe(II)-bearing minerals such as vivianite, siderite and pyrite, have not yet been tested, but they could potentially support growth by providing a source of Fe(II), similar to the mineral FeS which releases Fe²⁺ continuously. This would be a promising alternative to using Fe(II) sulphate or chloride, both of which dissolve in the medium (batch culture). While minerals would slowly release Fe²^+^, the Fe²^+^ concentration might never exceed a potential toxicity threshold.

**Fig. 7.**
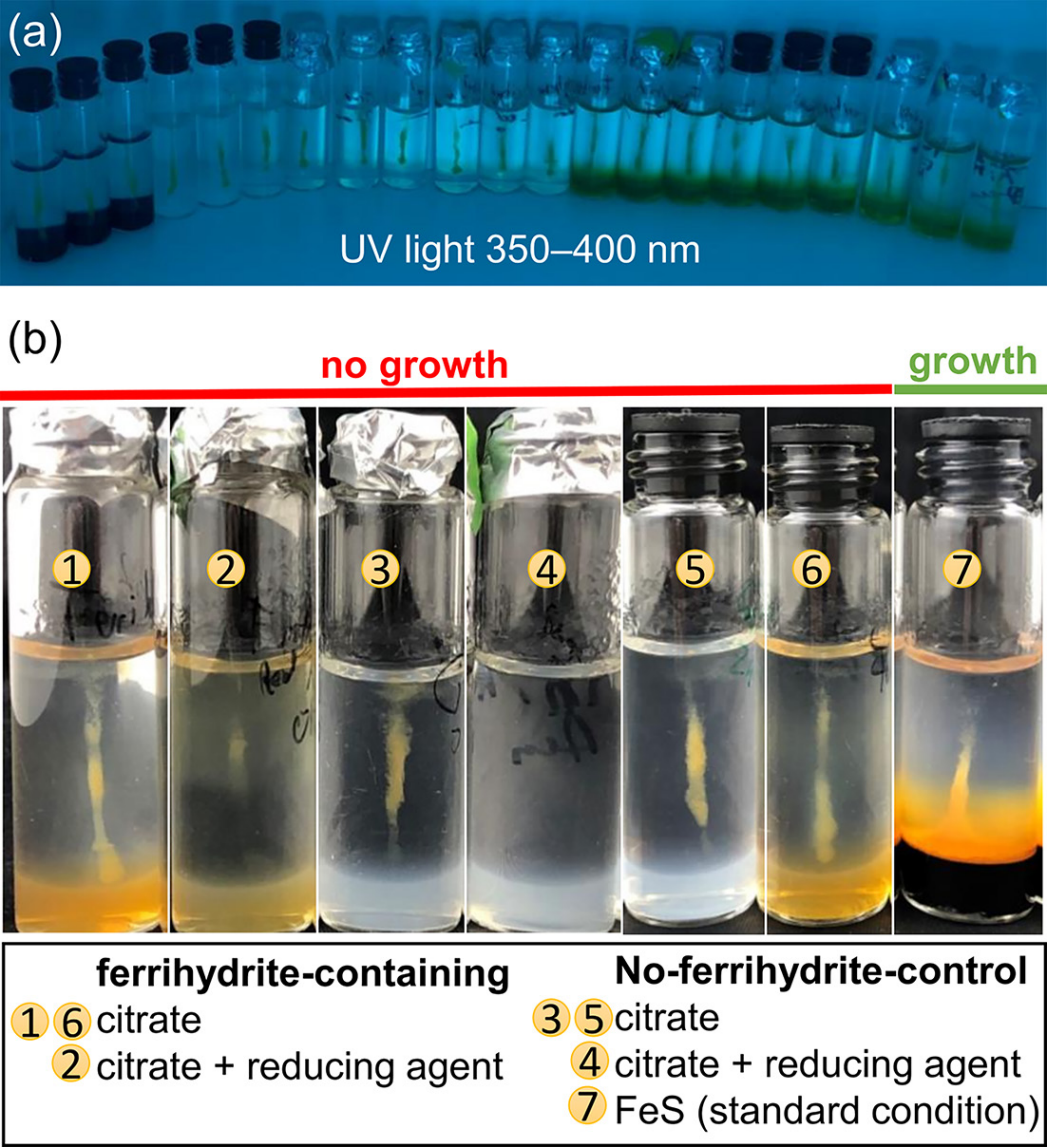
Assay for the utilization of photochemically produced Fe(II) as an electron donor by ‘*Ca*. F. straubiae’. (**a**) Experimental setup showing agarose-stabilized Fe(II)-O_2_ gradient tubes in a UV-irradiated chamber. Open systems were loosely covered with aluminium foil, while closed systems were sealed with butyl stoppers. The experiment was performed in biological triplicates. (**b**) Images of individual setups after 9 days of UV irradiation at 25 °C. Growth supported by photochemically produced Fe(II) was evaluated in setups 1, 2 and 6. However, we did not see growth under these conditions. Setups 3–5 served as negative controls, while setup 7, showing the characteristic pyramidal cloud of Fe(III) oxyhydroxide minerals indicative of growth, functioned as a positive control.

Fe(II) produced by Fe(III) photoreduction from a ferrihydrite [Fe(OH)_3_] plug was also tested for the growth of ‘*Ca*. F. straubiae’, similar to Lueder *et al.* [24]. For a continuous cycle of abiotic oxidation of iron(II) by atmospheric oxygen and phototrophic reduction by UV light and citrate as an electron donor, we set up open systems (Fig. 7, setups 1 and 2). To reduce oxygen toxicity, we added reducing agents previously reported to enhance the growth of anaerobic and microaerophilic bacteria under fully oxic conditions (Fig. 7, setup 2) [56].

In addition, we had a setup that was closed, as this had previously been successfully demonstrated to work for microaerophilic bacteria by Lueder *et al.* [24] (Fig. 7; setups 6). While ‘*Ca*. F. straubiae’ did not grow on citrate and reducing agents alone (Fig. 7; setups 3, 5 and 4, control without ferrihydrite), they were also unable to utilize photochemically produced Fe(II) in combination with citrate (Fig. 7; setups 1, 2 and 6 with ferrihydrite). There was also a positive control exposed to the UV light which showed comparable growth of ‘*Ca*. F. straubiae’ to the setups run in parallel in the dark at 25 °C (Fig. 7; setup 7), demonstrating that the UV light did not inhibit the growth of ‘*Ca*. F. straubiae’.

#### ‘*Ca*. F. straubiae’ utilizing alternative substrates in agarose-stabilized medium

Several alternative substrates for ‘*Ca*. F. straubiae’ were tested in triplicate in agarose-stabilized O_2_-gradient tubes. These were sodium acetate (10 mM), glucose (10 mM), sodium pyruvate (10 mM), sodium citrate (10 mM), sodium thiosulphate (5 mM), sodium sulphide (5 mM) or MnCl_2_ (10 mM) added to a plug at the bottom of the gradient tube and 1% H_2_ in the headspace. Under the experimental conditions, ‘*Ca*. F. straubiae’ showed no growth on Fe(II)-free medium containing the tested inorganic or organic substrates.

#### Assessment of denitrification by ‘*Ca*. F. straubiae’ under autotrophic and heterotrophic conditions within a pH range of 5.5–8.2

‘*Ca*. F. straubiae’ was evaluated in triplicate for its denitrification ability as a pure strain using liquid media (22 mM bicarbonate MWMM). The autotrophic conditions contained 10 mM Fe(II) and either nitrate or nitrite (4 mM). The heterotrophic conditions contained 10 mM acetate and either nitrate (20 mM) or nitrite (10 mM). As *Geobacter metallireducens*, a nitrate-reducing Fe(II)-oxidizer, was been reported to grow well in the presence of Fe(III)-citrate (0.5 mM) [57] and ascorbic acid (1 mM) [58,59] when utilizing Fe(II), acetate and nitrate as substrates, these supplements were added to the heterotrophic setups used for the here-studied ‘*Ca*. F. straubiae’ cultures. All four conditions were tested within a pH range of 5.5 to 8.2 (specifically 5.5, 6.0, 6.6, 7.0, 7.5 and 8.2). In none of the setups was growth observed.

These findings support the widely accepted hypothesis that ‘*Ca*. F. straubiae’ does not function as a nitrate-reducing Fe(II)-oxidizer on its own, but rather within the microbial community of culture KS, particulaly under autotrophic conditions [6,17, 19]. This conclusion is based on previous observations that it could not be isolated using nitrate and Fe(II) alone and that its genome lacks a canonical *nor* gene [19].

Since NO (the product of NO^_2_-^ reduction by Nir) is a highly cytotoxic gas that readily reacts with biomolecules, bacteria require a detoxification strategy. This strategy may involve (i) a Nor complexed with Nir for rapid NO degradation, (ii) a nitric oxide dismutase (Nod) generating intracellular oxygen, or (iii) a community member capable of NO reduction [19,20, 60,62]. Indeed, it has been hypothesized that ‘*Ca*. F. straubiae’ possesses a Nod that produces *‘*dark oxygen,’ which could explain the expression of oxygen-respiratory genes under anoxic conditions [19,63, 64]. However, we could not identify any sequence homologues of canonical Nods or recently described novel Nods (GenBank: APP93273.1; CBE69502.1 and APP93283.1), encoded in the genome of ‘*Ca*. F. straubiae’ (IMG Genome ID: 2878407288) [62]. Therefore, case (ii) seems unlikely, although the existence of an unknown Nod cannot be entirely ruled out.

Despite our efforts to identify optimal conditions for nitrate-reducing Fe(II) oxidation by ‘*Ca*. F. straubiae’ across a range of pH levels, Fe(II) oxidation was not observed under any of the tested conditions. Taken together with the absence of canonical *nor* and *nod* genes, ‘*Ca*. F. straubiae’ likely has a truncated denitrification pathway and thus relies on the 2% flanking community of culture KS, aligning with the NO detoxification strategy (iii) [19].

Identifying bacteria with truncated denitrification pathways requires special care, however Lycus *et al.* [61] successfully isolated 70 denitrifier representatives with truncated denitrification chains, four of which also lacked the ability to reduce NO. Their work provides another great example of this phenomenon, which, although known to occur, has not been well described in the literature [60,61].

Notably, we isolated ‘*Ca. F.* straubiae’ using Fe(II)/nitrate-containing medium (section 'Challenges of growing single colonies and isolation by the dilution-to-extinction method'). Thus, uncertainty remains, leaving us to wonder whether some overlooked factors – such as a growth inhibitor, less-concentrated sodium bicarbonate buffer resulting in lower salinity, a different Fe(II)/nitrate ratio, temperature variations or a different and lower concentrated Fe(II) source – might be influencing the outcome. We also tested the aforementioned liquid medium (pH 6.5) with extra trace elements added, as described in the section 'Modified trace element solution for sustainable growth on agarose-stabilized Fe(II)-O_2_ gradient tubes' for growth, but were unsuccessful.

### Maintenance and long-term storage of ‘*Ca*. F. straubiae’ – importance of FeS, micronutrients and trace element

While maintaining the ‘*Ca*. F. straubiae’ culture on agarose-stabilized Fe(II)-O_2_ gradient tubes, we noticed that not all transfers were successful, and by examining all tubes over several transfers, we found that ‘*Ca*. F. straubiae’ showed a very long viability of at least 1 year. Nevertheless, we had to find a condition in which ‘*Ca*. F. straubiae’ can be maintained sustainably.

#### Evaluation of different agarose-stabilized Fe(II)-O_2_ gradient tube protocols including the supplementation of tap water

For the following reasons, we suspected that selenium/tungstate supplementation, NH_4_Cl concentration and the FeS batch were primary factors that controlled the success of maintaining ‘*Ca*. F. straubiae’.

Culture maintenance was performed using gradient tubes prepared with slightly different protocols. Early subcultures of the ‘*Ca*. F. straubiae’ isolate (Iso6) were successfully grown in gradient tubes containing selenium/tungstate and a lowered NH_4_Cl concentration of 0.1 g l^−1^ according to the protocol of Lueder *et al.* [24]. However, in our laboratory, we also routinely prepare gradient tubes without selenium/tungstate and with 1 g l^−1^ NH_4_Cl as described by Emerson and Floyd [27]. In the experiments conducted in the section 'Physiological characterization of ‘*Ca*. F. straubiae’', 1 g l^−1^ NH_4_Cl was used, which is the standard concentration for MWMM. In addition, selenium/tungstate was included because it was an important supplement in the growth medium of the KS enrichment culture from which ‘*Ca*. F. straubiae’ was derived. These variations in medium composition likely contributed to the inconsistent growth behaviour observed across multiple generations of ‘*Ca*. F. straubiae’ cultures over 3 years.

Another critical factor was the batch of FeS used in the gradient tubes. We observed that, in particular, one batch of FeS consistently supported growth (FeS_good_ batch), while the use of every other batch in our lab failed. Given the limited availability of FeS_good_, we decided to not only investigate the effect of selenium/tungstate supplementation and NH_4_Cl concentration on ‘*Ca*. F. straubiae’ growth but also aimed for a condition that compensates for slight differences in the FeS batch. We hypothesized that nanomolecular impurities of metals in FeS batches might play a role. Given the importance of micronutrients and trace elements, we examined the effects of replacing Millipore water in the growth medium with tap water (tap water-medium). Tap water is, by nature, a complex medium component and has previously been reported by Kucera and Wolfe [11] to support the maintenance of microaerophilic Fe(II)-oxidizers. In addition, we tested whether increasing the concentration of trace elements in the medium could enhance growth. This approach is more consistent with the concept of a defined medium composition and provides a more sustainable solution for supporting growth than using a complex medium component such as tap water.

**Fig. 8.**
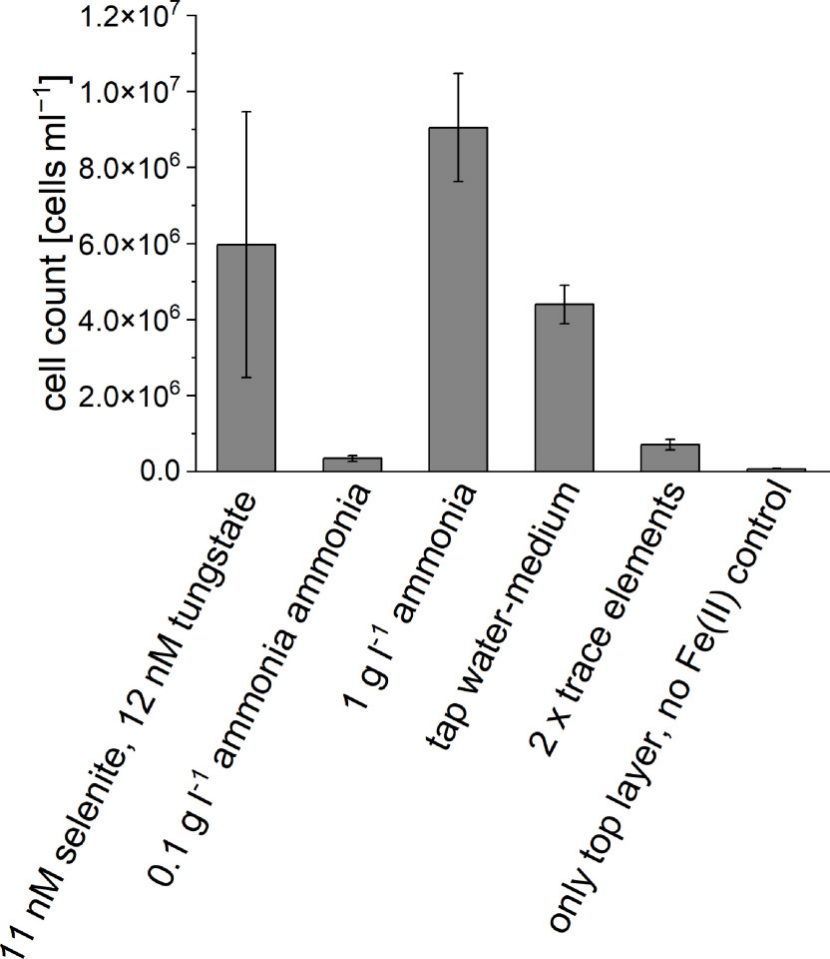
Evaluation of growth effects due to ingredient modifications of agarose-stabilized Fe(II)-O_2_ gradient tubes. Cells were counted via flow cytometry after 1 month of incubation at room temperature. Error bars represent biological triplicates.

The four criteria – (i) selenium/tungstate supplementation, (ii) NH_4_Cl concentration, (iii) use of tap water-medium and (iv) trace element concentration – were investigated under the use of FeS_good_ in the FeS plug. We found that selenium/tungstate supplementation improved growth, resulting in a 17.2 times higher cell number compared to the control setup without selenium/tungstate (Fig. 8). The use of 1 g l^−1^ NH_4_Cl (standard MWMM) promoted 26.1 times greater growth compared to 0.1 g l^−1^ NH_4_Cl (medium after Lueder *et al.* [24]). When tap water-medium was used in the upper layer of the gradient tube, the cell number increased 12.7-fold compared to the reference culture prepared with Millipore water-medium. Doubling the trace element concentration resulted in a twofold increase in cell growth compared to the reference culture prepared with standard trace element concentrations.

The supplementation with selenite/tungstate indicates that either selenite- or tungstate-containing enzymes (or both) play a role for the autotrophic growth of ‘*Ca*. F. straubiae’ on Fe(II)/O_2_. Thus, omitting this supplement is a growth-limiting factor. Further, our results indicate that NH_4_Cl, functioning as nitrogen source in the medium, is a growth-limiting factor at a concentration of 0.1 g l^−1^. Supplementing twice as much trace elements than the standard concentration of SL-10 did enhance growth; however, it was 6.2 times less efficient than the complex additive of tap water. If tap water can indeed sustain the ‘*Ca*. F. straubiae’ culture, it would offer a simple, long-term solution. The experiment shown in Fig. 8 was conducted by using FeS_good_ which was expected to support growth in all setups at least to a certain grade. Although tap water clearly showed a positive effect on growth, it remains elusive whether the positive effect of using tap water is sustainable when using different FeS batches. Therefore, a different FeS batch will be examined in the following experiment.

**Fig. 9.**
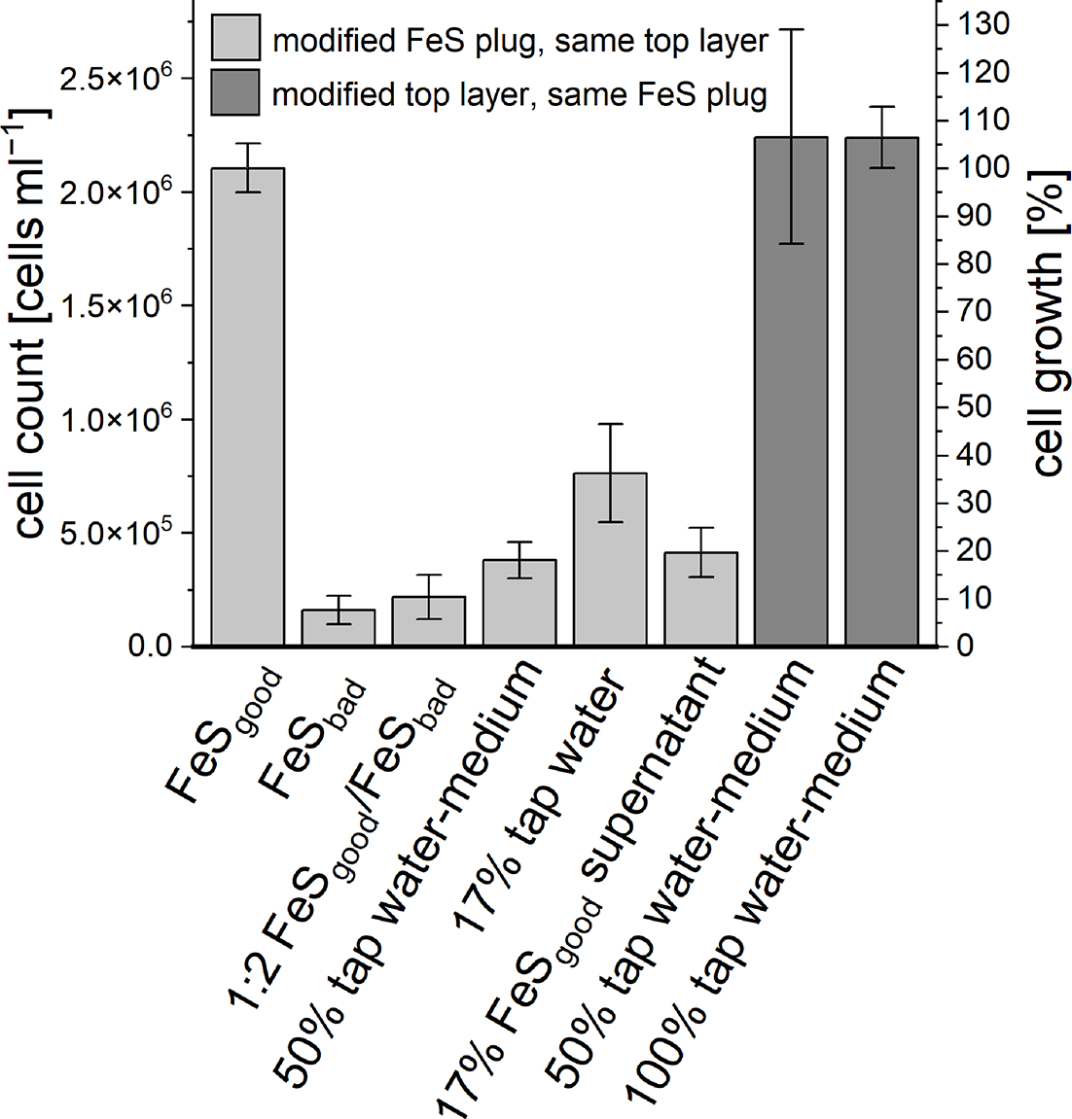
Evaluation of the different batches of FeS and the use of tap water on the growth of ‘*Ca*. F. straubiae' in agarose-stabilized Fe(II)-O_2_ gradient tubes. The first bar represents cultures grown with a FeS batch (FeS_good_) that consistently supported robust growth. The second bar corresponds to cultures using a FeS batch observed to result in reduced or no growth (FeS_bad_). The third bar represents cultures grown with a mix of both FeS batches (1 : 2 ratio of FeS_good_ to FeS_bad_). The fourth bar corresponds to a setup where the medium (typically prepared with Millipore water) in the FeS_bad_ plug was fully replaced with medium prepared with tap water (50% FeS_bad_+50% tap water-medium). In the fifth and sixth setups, the FeS_bad_ plug contained 17% tap water or the supernatant of the favourable FeS_good_ batch, respectively, with the supplement replacing Millipore medium. In the seventh and eighth setups, the top layer was replaced with 50% tap water-medium (the other 50% being Millipore medium) and 100% tap water-medium, respectively. Cells were counted via flow cytometry after 1 month of incubation at room temperature. Error bars indicate biological triplicates.

While hypothesizing that a nanoscale impurity of FeS_good_ might be the growth-enhancing factor, we must also consider a reverse scenario in which a nanoscale impurity of the other FeS batches might be a growth-inhibiting factor. In the following, we present the investigation of different modifications involving tap water in preparing gradient tubes and a variation on setups investigating the effect of two different FeS batches. We used the FeS_good_ batch (2 years old at the time point when performing the experiment) as a reference and another batch (FeS_bad_, 1 year old) from our laboratory for comparison, with the goal of improving the 'FeS_bad_ condition' to match the growth efficiency of the better FeS batch. We tested different methods of introducing tap water into the gradient tube, either by adding it to the FeS plug or to the medium used for the top layer. The aim was to assess how much growth efficiency (100%; Fig. 9) could be restored by each adaptation. Further, we investigated the growth-supporting effect of using some FeS_good_ (ratio 1 : 2 of FeS_good_ to FeS_bad_ in the FeS plug) or using supernatant of the bottle storing the FeS_good_ added to the FeS plug (17%, replacing medium). As the growth was slightly increased by the adaptation of using FeS_good_ or supernatant of FeS_good_, it indicates that there was nothing about FeS_bad_ which inhibited growth (Fig. 9).

The results based on tap-water adaptations showed that using tap water in the top layer was prior over the effect of introducing tap water to the FeS plug (Fig. 9). Fifty per cent tap water-medium in the top layer was sufficient to restore full growth efficiency. However, the tests were conducted using a preculture pre-grown on FeS_good_. Therefore, the results in Fig. 9 show that growth deficiency as observed using FeS_bad_ alone can be restored using tap water in the top layer.

Based on the drinking water report from the local municipal utility company, Stadtwerke Tübingen, we designed an artificial tap water that focuses on the major components: Mg^2+^, Ca^2+^, PO₄^3−^ and CO₃²⁻. We prepared the medium identical to the MWMM but adjusted the concentrations of these components to correspond to the ‘Morgenstelle’ region of Tübingen drinking water (0.6 g l^−1^ MgSO₄·7 H₂O, 0.3 g l^−1^ CaCl₂·2 H₂O, 0.05 g K₂HPO₄ and 1.01 g NaHCO₃) [65]. We then used this new medium to prepare gradient tubes according to the standard protocol. We hypothesized that the phosphate sources in tap water might be more bioavailable than K₂HPO₄, which can react with Fe(II) to form vivianite [66]. Therefore, we performed preliminary tests by supplementing the top layers of standard gradient tubes, as well as tubes prepared with the new medium, with *β*-glycerophosphate disodium salt hydrate (final concentrations of 0.06 g l^−1^ and 0.267 mM), after sterilization. Neither attempt to substitute tap water showed improvement in bacterial growth. Therefore, we considered using tap water for culture maintenance. However, whether this adaptation can sustain growth over multiple transfers remains elusive. The sustainability of tap water will be issued in the section 'Modified trace element solution for sustainable growth on agarose-stabilized Fe(II)-O_2_ gradient tubes'.

#### Effect of FeS ageing on the growth of ‘*Ca*. F. straubiae’ in agarose-stabilized Fe(II)-O_2_ gradient tubes

We also investigated the effect of FeS ageing and FeS concentration in the bottom Fe(II) plug. The experiment was conducted with 1- and 2-year-old FeS batches, as well as with a ½-year-old batch that had been washed five times with distilled water (standard procedure when synthesizing FeS) after FeS production and one that had been washed four times. The pH of all the FeS batches was neutral.

**Fig. 10.**
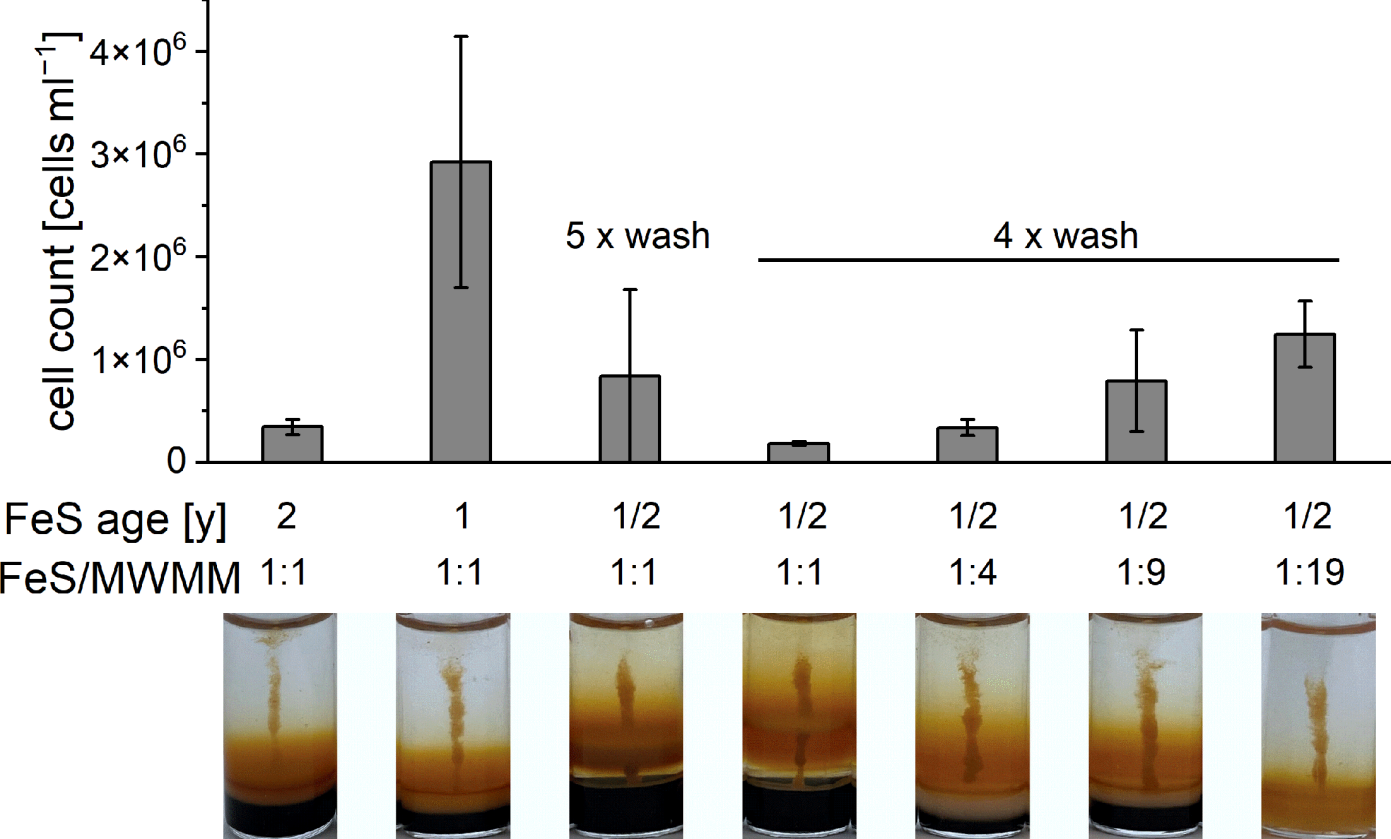
Evaluation of growth effects due to different aged and washed batches of FeS in agarose-stabilized Fe(II)-O_2_ gradient tubes. Cells were counted via flow cytometry after 1 month of incubation at room temperature. Error bars indicate biological triplicates.

The more Fe^2+^ was released from the FeS into the overlaying agar forming the Fe^2+^ gradient, the higher and more intense the abiotic oxidation zone formed (Fig. 10). Optimal growth of ‘*Ca*. F. straubiae’ was observed when Fe^2+^ release was minimal, and the oxidation ring appeared just above the FeS plug. This was observed using the 1- and 2-year FeS batches (Fig. 10, column bars 1 and 2). Setups using a FeS plug with FeS to medium ratios of 1 : 1, 1 : 4, 1 : 9 and 1 : 19 indicated that by using less FeS, the Fe^2+^ concentration can be reduced to levels more favourable for growth (Fig. 10, columns 4–7). However, this results in a more rapid depletion of the Fe(II) reservoir compared to a plug with a higher FeS concentration using an older batch. Consequently, we observed less growth for the FeS plugs with lower FeS concentration.

The oxidation ring resulting in setups with the FeS that was washed five times with distilled water was located at a lower position in the tube than in setups with the FeS that was washed four times (Fig. 10, images under columns 3 and 4), indicating that more washing of the FeS plug lowers the amount of fast-releasing Fe^2+^, resulting in lower Fe^2+^ concentrations in the top layer. Since the FeS that was washed five times led to a higher cell number, more washing of the FeS (lowering obviously its solubility) might support greater growth as indicated by our findings (Fig. 10, columns 3 and 4). It remains to be determined whether a newly prepared batch can be washed extensively (to remove the easily mobilizable and fast-releasing Fe^2+^) to achieve the same quality as an older batch which has a lower amount of fast-releasing Fe^2+^.

While observing different heights and intensities of the iron oxidation zone in the gradient tubes – which clearly indicate that the FeS batches released varying amounts of Fe^2+^ into the top layer – the dissolution rate of Fe^2+^ can be directly linked to bacterial growth. When Fe²^+^ dissolves from FeS, HS⁻ is formed. This, in addition to a high Fe²^+^ concentration, may affect growth. Toxicity tests using HS⁻ could reveal whether Fe²^+^, HS⁻ or a combination of both affects growth. It is possible that a high Fe²^+^ concentration is toxic to the cells or that it solely changes the kinetics of the system towards a faster abiotic Fe(II) oxidation rate, which, in turn, reduces biological oxidation and growth. It may be difficult to distinguish between these two possibilities experimentally.

Furthermore, other more complex factors than just the age of the FeS batch must also be considered. The 2- and 1-year-old batches were prepared by the same individual (Lars Grimm, AG Kappler), whereas the two younger batches were prepared collaboratively by Dr. Verena Nikeleit and MSc Phillip Liebknecht (AG Kappler). Despite following the same protocol, the younger batches were found to contain larger FeS particles that clogged pipette syringes (not shown), demonstrating that the batches differ in more than just age. Additionally, the FeS was stored in bottles filled completely with oxic distilled water, and these containers were opened and refilled with oxic water at different times for gradient tube preparation.

Age, the individual scientist producing the FeS and the frequency of use were shown to affect FeS chemistry and, therefore, its quality (mainly its solubility). Considering the complexity of iron sulphide chemistry, including changes over time, particle size and dissolution kinetics as a function of system equilibrium [67,70], further experiments are necessary to fully interpret these findings. Additionally, it has yet to be tested whether using tap water to wash FeS once, or throughout all synthesis steps (to prevent FeS hydrosols), would enhance bacterial growth [1,10]. The primary conclusion from this experiment is that a low rate of continuous Fe(II) dissolution promotes greater cell growth.

#### Modified trace element solution for sustainable growth on agarose-stabilized Fe(II)-O_2_ gradient tubes

In the previous experiments, we determined differences in ‘*Ca*. F. straubiae’ growth in gradient tubes due to protocol alterations including selenium/tungstate supplementation, NH_4_Cl concentration and modifications to introducing tap water and use of different batches of FeS. The results indicated that optimum growth conditions include supplementing the medium with selenite/tungstate, preparing the FeS plug using at least a 1-year-old FeS batch and using MWMM prepared with tap water for the top layer. As the tests were carried out using a well-grown culture from the FeS_good_ batch (the FeS batch consistently supporting growth), it remained unclear whether these factors were a sustainable approach to culture maintenance. We therefore transferred the culture to gradient tubes following the above criteria for optimum growth. After three transfers on a monthly basis, the growth was drastically reduced. Therefore, we have not determined the exact composition of our tap water in order to make reproducible conditions by artificial tap water. This finding pushed us for further medium optimization which was more targeted than the simple use of a complex medium ingredient such as tap water. We aimed to achieve this goal by modifying the trace element solution and focused on those that were previously reported to improve bacterial activity such as As, Ni, Cu, Se, Mo [71,72] and the 'newer' essential trace element vanadium [73], which is abundant in the groundwater of Bremen [74], the origin of culture KS that contains ‘*Ca*. F. straubiae’. After two rounds of optimization, both with normal MWMM (made with Millipore water) or MWMM prepared with tap water in the top layer and with different combinations of these trace elements, we decided to use all the additives tested, as none of them seemed to have an inhibitory effect. Notably, the medium for all setups was supplemented with the standard trace element solution as well. Each condition of the two optimization rounds was performed as duplicates. The gradient tubes were monitored for the presence of a pyramidal cloud of iron(III) oxyhydroxide minerals, indicating growth (as shown in Fig. 2), and were analysed by fluorescence microscopy after 1 month of incubation (data not shown). Interestingly, the setups containing normal MWMM performed better than those with tap water. According to the results, the final concentration of extra trace elements in the upper layer was adjusted to 10 µM Na_2_SeO_3_, 10 µM Na_2_MoO_4_, 0.1 µM NiCl_2_, 0.1 µM CuCl_2_, 0.1 µM AsO_3_ and 5 nM NH_4_VO_3_. Since this adjustment, the culture has been transferred several times on an ~1-year-old batch (different from FeS_good_) on normal MWMM without loss of growth.

## Conclusion – recommendations for cultivation of ‘*Ca*. F. Straubiae’

As a pure strain, ‘*Ca*. F. straubiae’ remained unculturable with all substrates tested, with the exception of Fe^2+^ released from FeS in combination with O_2_ as the electron acceptor. Therefore, we grew ‘*Ca*. F. straubiae’ on agarose-stabilized Fe(II)-O_2_ gradient tubes prepared from a rather old batch of FeS than a fresh one. If your laboratory does not have FeS older than 1 year, we recommend washing the FeS to neutral pH at least five times. Trials with different concentrations of FeS in the plug will help to find growth-promoting conditions. A well-grown culture can be recovered from tubes as old as 1 year if the FeS plug has residual Fe(II) minerals. We also recommend the use of MWMM (1 g l^−1^ NH_4_Cl) supplemented with 7-vitamins, SL-10 trace elements, selenite-tungstate solution and selenite-molybdate-nickel-copper-arsenic-vanadium solution (final concentrations of 10 µM Se, 10 µM Mo, 0.1 µM Ni, 0.1 µM Cu, 0.1 µM As and 5 nM V). The bacteria grow well at room temperature and pH 6.5. From our experience, we recommend using SYTOX^™^ Green (Invitrogen, Thermo Fisher Scientific, Inc.) to stain heat-treated ‘*Ca*. F. straubiae’, when examining growth by fluorescence microscope. The stain is very bright, and 2.5 µM of stain is sufficient. A working solution of the stain (5 µM) can be made by using H_2_O and storage at 4 °C for months.

## Description of *Ferrigenium straubiae* sp. nov.

*Ferrigenium straubiae* (strau’bi.ae. N.L. gen. n. *straubiae*, named after Kristina Straub in honour of her original isolation of the enrichment culture KS). The cells are slightly curved rods (0.7–2 µm) and are Gram-negative. This mesophilic, neutrophilic, non-halophilic organism grows autotrophically under microoxic conditions, using Fe(II) from FeS as the electron donor and O₂ as the electron acceptor in agarose-stabilized medium. Cultured under this condition, the bacterium was non-stalk-forming, and a flagellum could not be observed using SEM. Growth occurs at 20–30 °C (optimum 25 °C) and pH 6.0–7.5 (optimum 6.5–7.0). It does not utilize hydrogen, thiosulphate, sulphide, nitrite, nitrate, Mn²^+^, glucose, acetate, pyruvate or citrate as an energy source. This bacterium belongs to the *Gallionellaceae* family, and the type strain is KS (DSM 118991, KCTC 25982). Strain KS was isolated from the 26-year-old laboratory-maintained enrichment culture KS, which was originally collected from freshwater sediment in Bremen, Germany (53.10971 N 8.84510 E).

The bacterium has two distinct copies of the 16S rRNA gene, the full-length sequences gained from Illumina sequencing are deposited at GenBank under the accession numbers PV740118 and PV740119 and the partial sequences gained from Sanger sequencing are under the accession numbers PV739805, PV739806, PV739807 and PV739808. Additionally, the full-length 16S rRNA genes were annotated in the IMG database and can be accessed using the following gene IDs: 8132340740 and 8132340651 [48,49].

Huang *et al.* [6] initially proposed the new taxon as ‘*Candidatus* Ferrigenium straubiae’ based on a phylogenetic analysis that differentiated closely related taxa. Table 2 lists phenotypic and genotypic comparisons to closely related strains.

## Supplementary material

10.1099/ijsem.0.006949 Uncited Supplementary Material 2.

## Abbreviations

gDNA: genomic DNA
HAHCl: hydroxylammonium hydrochloride
HMDS: hexamethyldisilazane
MWMM: modified Wolfe’s mineral medium
Nir: nitrite reductase
NO: nitric oxide
Nod: nitric oxide dismutase
Nor: nitric oxide reductase
NRFeOx: nitrate-reducing Fe(II)-oxidizing
PI: propidium iodide
SEM: scanning electron microscopy
ZVI: zero-valent iron

## References

[1] Hanert HH, Dworkin M, Falkow S, Rosenberg E (2006). The Prokaryotes: Volume 7: Proteobacteria: Delta, Epsilon Subclass.

[2] Emerson D, Moyer C (1997). Isolation and characterization of novel iron-oxidizing bacteria that grow at circumneutral pH. Appl Environ Microbiol.

[3] Kato S, Ohkuma M, Powell DH, Krepski ST, Oshima K (2015). Comparative genomic insights into ecophysiology of neutrophilic, microaerophilic iron oxidizing bacteria. Front Microbiol.

[4] Khalifa A, Nakasuji Y, Saka N, Honjo H, Asakawa S (2018). *Ferrigenium kumadai* gen. nov., sp. nov., a microaerophilic iron-oxidizing bacterium isolated from a paddy field soil. Int J Syst Evol Microbiol.

[5] Straub KL, Benz M, Schink B, Widdel F (1996). Anaerobic, nitrate-dependent microbial oxidation of ferrous iron. Appl Environ Microbiol.

[6] Huang Y-M, Jakus N, Straub D, Konstantinidis KT, Blackwell N (2022). ‘*Candidatus* ferrigenium straubiae’ sp. nov., ‘*Candidatus* ferrigenium bremense’ sp. nov., ‘*Candidatus* ferrigenium altingense’ sp. nov., are autotrophic Fe(II)-oxidizing bacteria of the family Gallionellaceae. Syst Appl Microbiol.

[7] Ehrenberg CG (1836). Vorläufige Mittheilungen über das wirkliche Vorkommen fossiler Infusorien und ihre grosse Verbreitung. Annalen der Physik.

[8] Rohrer E (1952). *Gallionella ferruginea* Ehrenberg, ein Beitrag zur Kenntnis der Eisenbakterien. Schweiz Z Hydrologie.

[9] Griffith JW (1853). XLIV.— On *Gallionella ferruginea* (Ehrenb.). Annals and Magazine of Natural History.

[10] Hanert H (1968). Untersuchungen zur Isolierung, Stoffwechselphysiologie und Morphologie von *Gallionella ferruginea* Ehrenberg. Archiv Mikrobiol.

[11] Kucera S, Wolfe RS (1957). A selective enrichment method for *Gallionella ferruginea*. J Bacteriol.

[12] Engel H, Hanert H (1967). Isolation of *Gallionella ferruginea* Ehrenberg. Naturwissenschaften.

[13] Wolfe RS (1958). Cultivation, morphology, and classification of the iron bacteria. Journal AWWA.

[14] Huang Y-M, Straub D, Kappler A, Smith N, Blackwell N (2021). A novel enrichment culture highlights core features of microbial networks contributing to autotrophic Fe(II) oxidation coupled to nitrate reduction. *Microb Physiol*.

[15] Jakus N, Blackwell N, Straub D, Kappler A, Kleindienst S (2021). Presence of Fe(II) and nitrate shapes aquifer-originating communities leading to an autotrophic enrichment dominated by an Fe(II)-oxidizing *Gallionellaceae* sp. FEMS Microbiol Ecol.

[16] Grimm H, Lorenz J, Straub D, Joshi P, Shuster J (2025). Nitrous oxide is the main product during nitrate reduction by a novel lithoautotrophic iron(II)-oxidizing culture from an organic-rich paddy soil. Appl Environ Microbiol.

[17] He S, Tominski C, Kappler A, Behrens S, Roden EE (2016). Metagenomic analyses of the autotrophic Fe(II)-oxidizing, nitrate-reducing enrichment culture KS. Appl Environ Microbiol.

[18] Becker S, Enright AML, Kappler A (2021). 7 LIVING ON IRON De Gruyter.

[19] Huang Y-M, Straub D, Blackwell N, Kappler A, Kleindienst S (2021). Meta-omics reveal *Gallionellaceae* and *Rhodanobacter* species as interdependent key players for Fe(II) Oxidation and nitrate reduction in the autotrophic enrichment culture KS. Appl Environ Microbiol.

[20] Terasaka E, Yamada K, Wang P-H, Hosokawa K, Yamagiwa R (2017). Dynamics of nitric oxide controlled by protein complex in bacterial system. Proc Natl Acad Sci USA.

[21] Albertsson I, Sjöholm J, Ter Beek J, Watmough NJ, Widengren J (2019). Functional interactions between nitrite reductase and nitric oxide reductase from *Paracoccus denitrificans*. Sci Rep.

[22] Hegler F, Posth NR, Jiang J, Kappler A (2008). Physiology of phototrophic iron(II)-oxidizing bacteria: implications for modern and ancient environments. FEMS Microbiol Ecol.

[23] Widdel F, Bak F, Balows A, Trüper HG, Dworkin M (1992). The Prokaryotes: A Handbook on the Biology of Bacteria: Ecophysiology, Isolation, Identification, Applications.

[24] Lueder U, Maisch M, Jørgensen BB, Druschel G, Schmidt C (2022). Growth of microaerophilic Fe(II)-oxidizing bacteria using Fe(II) produced by Fe(III) photoreduction. Geobiology.

[25] Pfennig N, Trüper HG, Starr MP, Stolp H, Trüper HD (1981). The Prokaryotes: A Handbook on Habitats, Isolation, and Identification of Bacteria.

[26] Laufer K, Nordhoff M, Røy H, Schmidt C, Behrens S (2016). Coexistence of microaerophilic, nitrate-reducing, and phototrophic Fe(II) oxidizers and Fe(III) reducers in coastal marine sediment. Appl Environ Microbiol.

[27] Emerson D, Floyd MM (2005). Enrichment and isolation of iron-oxidizing bacteria at neutral pH. *Methods Enzymol*.

[28] Stookey LL (1970). Ferrozine-a new spectrophotometric reagent for iron. Anal Chem.

[29] Schaedler F, Kappler A, Schmidt C (2018). A revised iron extraction protocol for environmental samples rich in nitrite and carbonate. Geomicrobiology Journal.

[30] Muyzer G, Teske A, Wirsen CO, Jannasch HW (1995). Phylogenetic relationships of Thiomicrospira species and their identification in deep-sea hydrothermal vent samples by denaturing gradient gel electrophoresis of 16S rDNA fragments. Arch Microbiol.

[31] Lane DJ, Pace B, Olsen GJ, Stahl DA, Sogin ML (1985). Rapid determination of 16S ribosomal RNA sequences for phylogenetic analyses. Proc Natl Acad Sci USA.

[32] Klindworth A, Pruesse E, Schweer T, Peplies J, Quast C (2013). Evaluation of general 16S ribosomal RNA gene PCR primers for classical and next-generation sequencing-based diversity studies. Nucleic Acids Res.

[33] Ewels PA, Peltzer A, Fillinger S, Patel H, Alneberg J (2020). The nf-core framework for community-curated bioinformatics pipelines. Nat Biotechnol.

[34] Di Tommaso P, Chatzou M, Floden EW, Barja PP, Palumbo E (2017). Nextflow enables reproducible computational workflows. Nat Biotechnol.

[35] Andrews S (2025). https://github.com/s-Andrews/FastQC.

[36] Chen S, Zhou Y, Chen Y, Gu J (2018). fastp: an ultra-fast all-in-one FASTQ preprocessor. Bioinformatics.

[37] De Coster W, D’Hert S, Schultz DT, Cruts M, Van Broeckhoven C (2018). NanoPack: visualizing and processing long-read sequencing data. Bioinformatics.

[38] Volkening J, Wick R, Nickloman (2018). PoreChop v0.2.4 - adapter trimmer for oxford nanopore reads. https://github.com/Rrwick/Porechop.

[39] Wood DE, Lu J, Langmead B (2019). Improved metagenomic analysis with Kraken 2. Genome Biol.

[40] Larsen MV, Cosentino S, Lukjancenko O, Saputra D, Rasmussen S (2014). Benchmarking of methods for genomic taxonomy. J Clin Microbiol.

[41] Wick RR, Judd LM, Gorrie CL, Holt KE (2017). Unicycler: Resolving bacterial genome assemblies from short and long sequencing reads. PLoS Comput Biol.

[42] Oxford Nanopore Technologies (2021). https://github.com/nanoporetech/medaka.

[43] Gurevich A, Saveliev V, Vyahhi N, Tesler G (2013). QUAST: quality assessment tool for genome assemblies. Bioinformatics.

[44] Manni M, Berkeley MR, Seppey M, Simão FA, Zdobnov EM (2021). BUSCO update: novel and streamlined workflows along with broader and deeper phylogenetic coverage for scoring of eukaryotic, prokaryotic, and viral genomes. Mol Biol Evol.

[45] Verran J, Stott JFD, Quarmby SL, Bedwell M (1995). Detection, cultivation and maintenance of *Gallionella* in laboratory microcosms. Lett Appl Microbiol.

[46] Eggerichs T, Wiegand M, Neumann K, Opel O, Thronicker O (2020). Growth of iron-oxidizing bacteria *Gallionella ferruginea* and *Leptothrix cholodnii* in oligotrophic environments: Ca, Mg, and C as limiting factors and *G. ferruginea* necromass as C-Source. Geomicrobiology Journal.

[47] Nunley JW, Krieg NR (1968). Isolation of *Gallionella ferruginea* by use of formalin. Can J Microbiol.

[48] Mukherjee S, Stamatis D, Li CT, Ovchinnikova G, Kandimalla M (2025). Genomes online database (GOLD) v.10: new features and updates. Nucleic Acids Res.

[49] Chen I-MA, Chu K, Palaniappan K, Ratner A, Huang J (2023). The IMG/M data management and analysis system v.7: content updates and new features. Nucleic Acids Res.

[50] Good NE, Winget GD, Winter W, Connolly TN, Izawa S (1966). Hydrogen ion buffers for biological research. Biochemistry.

[51] Ferreira CMH, Pinto ISS, Soares EV, Soares HMVM (2015). (Un)suitability of the use of pH buffers in biological, biochemical and environmental studies and their interaction with metal ions – a review. RSC Adv.

[52] Kato S, Chan C, Itoh T, Ohkuma M (2013). Functional gene analysis of freshwater iron-rich flocs at circumneutral pH and isolation of a stalk-forming microaerophilic iron-oxidizing bacterium. Appl Environ Microbiol.

[53] Winzig W, Püschel A, Herbeck L (2024). Klimareport Bremen und Bremerhaven. Deutscher Wetterdienst, Deutschland.

[54] Baker IR, Matzen SL, Schuler CJ, Toner BM, Girguis PR (2023). Aerobic iron-oxidizing bacteria secrete metabolites that markedly impede abiotic iron oxidation. PNAS Nexus.

[55] Becker S, Dang TT, Wei R, Kappler A (2025). Evaluation of *Thiobacillus denitrificans*’ sustainability in nitrate-reducing Fe(II) oxidation and the potential significance of Fe(II) as a growth-supporting reductant. FEMS Microbiol Ecol.

[56] La Scola B, Khelaifia S, Lagier J-C, Raoult D (2014). Aerobic culture of anaerobic bacteria using antioxidants: a preliminary report. Eur J Clin Microbiol Infect Dis.

[57] Senko JM, Stolz JF (2001). Evidence for iron-dependent nitrate respiration in the dissimilatory iron-reducing bacterium *Geobacter metallireducens*. Appl Environ Microbiol.

[58] Gorby YA, Lovley DR (1991). Electron Transport in the Dissimilatory Iron Reducer, GS-15. Appl Environ Microbiol.

[59] Weber KA, Urrutia MM, Churchill PF, Kukkadapu RK, Roden EE (2006). Anaerobic redox cycling of iron by freshwater sediment microorganisms. Environ Microbiol.

[60] Falk S, Liu B, Braker G (2010). Isolation, genetic and functional characterization of novel soil nirK-type denitrifiers. Syst Appl Microbiol.

[61] Lycus P, Lovise Bøthun K, Bergaust L, Peele Shapleigh J, Reier Bakken L (2017). Phenotypic and genotypic richness of denitrifiers revealed by a novel isolation strategy. ISME J.

[62] Bai M, He J, Zheng F, Lv S, Wang Z (2024). Gene cloning, expression and performance validation of nitric oxide dismutase. Science of The Total Environment.

[63] Ruff SE, Schwab L, Vidal E, Hemingway JD, Kraft B (2024). Widespread occurrence of dissolved oxygen anomalies, aerobic microbes, and oxygen-producing metabolic pathways in apparently anoxic environments. FEMS Microbiol Ecol.

[64] Sweetman AK, Smith AJ, de Jonge DSW, Hahn T, Schroedl P (2024). Evidence of dark oxygen production at the abyssal seafloor. Nat Geosci.

[65] Stadtwerke Tübingen (2023). Trinkwasserbericht 2023. https://www.swtue.de/wasser/trinkwasserbericht.html.

[66] Goedhart R, Müller S, van Loosdrecht MCM, van Halem D (2022). Vivianite precipitation for iron recovery from anaerobic groundwater. Water Res.

[67] Pankow JF, Morgan JJ (1979). Dissolution of tetragonal ferrous sulfide (mackinawite) in anoxic aqueous systems. 1. Dissolution rate as a function of pH, temperature, and ionic strength. Environ Sci Technol.

[68] Pankow JF, Morgan JJ (1980). Dissolution of tetragonal ferrous sulfide (mackinawite) in anoxic aqueous systems. 2. Implications for the cycling of iron, sulfur, and trace metals. Environ Sci Technol.

[69] Rickard D (2006). The solubility of FeS. Geochimica et Cosmochimica Acta.

[70] Rickard D, Luther GW (2007). Chemistry of iron sulfides. Chem Rev.

[71] Lester RL, DeMoss JA (1971). Effects of molybdate and selenite on formate and nitrate metabolism in *Escherichia coli*. J Bacteriol.

[72] Carboni MF, Arriaga S, Lens PNL (2024). Effect of copper, arsenic and nickel on pyrite-based autotrophic denitrification. Biodegradation.

[73] Mertz W (1974). The newer essential trace elements, chromium, tin, vanadium, nickel and silicon. Proc Nutr Soc.

[74] Hamer D, Hofmann A, Liedtke N (2013). Grundwassergütebericht 2013 der Freien Hansestadt Bremen. https://www.umwelt.bremen.de.

[75] Tominski C, Lösekann-Behrens T, Ruecker A, Hagemann N, Kleindienst S (2018). Insights into carbon metabolism provided by fluorescence *In Situ* hybridization-secondary ion mass spectrometry imaging of an autotrophic, nitrate-reducing, Fe(II)-oxidizing enrichment culture. Appl Environ Microbiol.

[76] Huang Y-M, Straub D, Kappler A, Smith N, Blackwell N (2021). A novel enrichment culture highlights core features of microbial networks contributing to autotrophic Fe(II) oxidation coupled to nitrate reduction. Microb Physiol.

[77] Jakus N, Mellage A, Höschen C, Maisch M, Byrne JM (2021). Anaerobic neutrophilic pyrite oxidation by a chemolithoautotrophic nitrate-reducing iron(II)-oxidizing culture enriched from a fractured aquifer. Environ Sci Technol.

[78] Emerson D, Field EK, Chertkov O, Davenport KW, Goodwin L (2013). Comparative genomics of freshwater Fe-oxidizing bacteria: implications for physiology, ecology, and systematics. Front Microbiol.

