# Supplementary material for: Isolation of ‘Candidatus Ferrigenium straubiae’ – a microaerophilic Fe(II)-oxidizing bacterium and nitrate-reducing Fe(II)-oxidizer within the community of culture KS
Source: Int J Syst Evol Microbiol. 2025 Nov 14;75(11):006949. doi: 10.1099/ijsem.0.006949 (PMC12622760; doi:10.1099/ijsem.0.006949)
Supplement: Uncited Supplementary Material 2. [file ijsem-75-06949-s001.pdf]

SUPPLEMENTAL INFORMATION

Isolation of "*Candidatus* Ferrigenium straubiae"—a microaerophilic Fe(II)-oxidizing bacterium and nitrate-reducing Fe(II)-oxidizer within the community of culture KS

Stefanie Becker[1] (ORCID iD: https://orcid.org/0009-0000-6909-797X), Andreas Kappler[1,2] (ORCID iD: https://orcid.org/0000-0002-3558-9500)

[1]Geomicrobiology, Department of Geosciences, University of Tübingen, Schnarrenbergstrasse 94–96, D-72076 Tübingen, Germany

[2]Cluster of Excellence: EXC 2124: Controlling Microbes to Fight Infection, Tübingen, Germany

Corresponding author and email address
Stefanie Becker, email: stefanie.becker@geo.uni-tuebingen.de

Text SI-1: Screening for alternative substrates

To identify conditions that support the maintenance of the pure "*Ca.* F. straubiae" culture, we designed a multi-condition screening that included various combinations of media, substrates, and amino acid concentrations. The media tested included Wolfe's medium, 30 mM bicarbonate-containing mineral medium, and an organic-rich medium (yeast nitrogen base (YNB) medium). These were paired with six substrate conditions and three amino acid concentrations: full concentration (according to the supplier's microbial growth protocol), half concentration, and one-tenth concentration. The amino acid mixture consisted of all standard amino acids at a concentration of 76 mg/L each except for leucine which had a concentration of 380 mg/L. Additionally it contained adenine (18 mg/L), inositol (76 mg/L) and p-aminobenzoic acid (8 mg/L). The six substrate conditions were:

1. sugar mixture/nitrate (4 mM)
2. sugar mixture/Fe(III) (4 mM)
3. sugar mixture/sulfate mixture
4. thiosulfate (20 mM)/nitrate (4 mM)
5. Fe(II) (10 mM)/acetate (2 mM)/nitrate (4 mM)
6. Fe(II) (10 mM)/acetate (2 mM)/sulfate mixture.

where the sugar mixture consisted of 0.25 mM glucose, 0.25 mM fructose, 0.25 mM saccharose, and 2 mM acetate, and the sulfate mixture consisted of 0.4 mM $Al_2(SO_4)_3$ and 0.4 mM Na_2SO_4. The screening was designed for testing multiple substrates within one condition to increase the chance to find an electron donor/acceptor couple which supports growth. Sulfate was included to test its usage as an electron acceptor (Plugge *et al.* 2011). The Fe(II)-free conditions (conditions 1–4) using 30 mM bicarbonate-containing mineral medium and YNM medium were set up twice. One was incubated anoxically and one under atmospheric oxygen concentration. The second set of incubations was done anoxically. Cultures were inoculated by using 10% of the sixth "*Ca.* F. straubiae" isolate (Iso6) obtained from an dilution to extinction method using Fe(II)-/nitrate-containing medium. The utilized preculture was the fifth transfer using Fe(II)-/nitrate-containing medium after its isolation. Cell growth was monitored by fluorescence microscopy. However, no growth of "*Ca.* F. straubiae" was observed in any of the substrate × medium × amino acid combinations.

Text SI-2: Temporal development of geochemical conditions in gradient tubes and consequences for microbial Fe(II) oxidation

It is important to note that the agarose-stabilized Fe(II)-oxygen gradient tubes were prepared 24 h before inoculation to allow the Fe^{2+} (dissolved Fe(II)) gradient to form while the headspace remained anoxic. The Fe^{2+} diffuses from the FeS plug at the bottom of the tube; there was no other source of iron during the course of the experiment. Thus, the measured "total iron" value refers to the iron released from FeS, which is considered to be Fe(II) before oxidation to Fe(III). After the initial incubation time (24 h), the top layer contained 0.25±0.02 mM Fe(II) (Fig. 3A), which steadily increased over the course of the experiment (rates in Fig. 5C). The majority of this Fe(II) is dissolved Fe^{2+}, however, over time some of it will adsorb to the produced Fe(III) minerals. Our extraction-based measurements capture all Fe(II) and we do not distinguish between adsorbed Fe(II) and Fe^{2+}.

24 h after preparing the gradient tubes, all tubes were opened, the headspace became oxygenated, and the biological setups were inoculated with "*Ca.* F. straubiae". In this early stages of the experiment, oxygen dissolved from the headspace into the agar layer and diffused rapidly downwards, reacting

abiotically with Fe(II) to form Fe(III). In the biotic setups some of the oxygen is used by the bacteria as electron acceptor for Fe(II) oxidation. The Fe(II) oxidation proceeds rapidly within 3 days after oxygenation and inoculation of the gradient tubes and subsequently slowed down (Fig. S2B). This change of the Fe(II) oxidation rate occurring between day 3 and 4 was significant, but not visible when looking at total Fe data as shown in the main manuscript (Fig. 1A) alone. In the following, we aim to explain this change of rate. For simplicity, we explain the relevant processes occurring in the abiotic setup. These abiotic processes are also happening in the biological setup; however, in the biological setups the Fe(II) oxidation is composed of three reactions: (i) biological Fe(II) oxidation coupled to oxygen reduction for energy conservation, (ii) biological Fe(II) oxidation to harness electrons for CO_2 fixation, (iii) the abiotic spontaneous oxidation of free Fe(II) by oxygen and of Fe(II) on mineral surfaces by oxygen (heterogeneous Fe(II) oxidation).

Since the tubes with the FeS were set-up already for 24 h before oxygenation, the Fe(II) gradient is established already and is oxidized very quickly (within the first 3 days) after opening the tubes to the atmosphere. Due to this pre-established Fe(II) gradient, the initial oxidation of Fe(II) was faster than the dissolution of Fe(II) from the FeS plug. Putting this into numbers: When opening the tubes to the atmosphere, about 0.25 mM Fe(II) was present in the top layer, and subsequently (in the following three days) about 0.65 mM Fe(II) was additionally released from the FeS plug. (Fe(II) concentration, Fe(II) oxidation rate and Fe(II) release rate is given in Fig. S1A in black, Fig. S2B and C, respectively.)

Hence, given the fast oxidation rate within the first 3 days, the Fe(II) in the top layer was oxidized faster than the rate at which fresh Fe(II) can be supplied from the FeS plug. Further oxidation requires that fresh Fe(II) reaches the oxidation front first, which explains the momentarily slow oxidation rate observed between day 3 and 4.

We were also curious whether this change in Fe(II) oxidation dynamics was primarily driven by the depletion of Fe(II) (due to limited diffusion and Fe(II) redissolution rates) or by the depletion of oxygen in the headspace. An estimate of the total amount of oxygen available in the headspace (3.865 mL at 1 atm, RT) is equivalent to 39.72 µmol (equation S1–3), which could theoretically oxidize 159.9 µmol of Fe(II) (1:4 molecular ratio (Stumm and Lee 1961)). 3 days after oxygenation, the total Fe(III) in the top layer of the abiotic setup was 2.3±0.2 µmol, indicating that only ~1.44% of the total available oxygen had been consumed. This suggests that oxygen limitation was not responsible for the observed change in Fe(II) oxidation rates.

Text SI-3: Reasoning for larger error bars in data sets after 3 days and occasionally negative values for 'Fe(II) oxidation' in abiotic setups

For the determination of Fe(II) oxidation rates and extents, we had to use sacrificial sampling, meaning the gradient tubes of each sampling point were unique, sampled completely at each time point and discarded afterwards. We observed larger variation in the obtained data after three days of incubation compared to the first three days and can explain this as follows: Due to fast Fe(II) oxidation within the first three days after oxygenation (average 0.2±0.05 mM/day), small differences between gradient tubes were not apparent, as any variability was masked by the fast initial abiotic iron oxidation kinetics. Because the Fe(II) oxidation rates slowed down considerably after three days (Fig. S2B), minor variations of measured Fe(II) and total Fe became more pronounced at sampling points after 3 days (Fig.1A and Fig. S1A). In some tubes, we even measured negative 'Fe(II) oxidation rates' in the abiotic setup (Fig. S2B), suggesting production of Fe(II). However, as we are not aware of any reduction process in our tubes we explain this observation due to experimental variability between the sampled gradient tubes. Indeed we believe that the Fe(II) oxidation rates were occasionally basically zero (between sampling days 3 and 4).

Text SI-4: Further analyses of total Fe, Fe(II), Fe(III) and cell counts, showing different perspectives on how these values correlate with each other

Evaluation of biotic vs. abiotic Fe(II) oxidation based on Fe(II) and Fe(III) concentrations

Although the inoculated tubes contained additional iron stemming from the inoculum, the difference in accumulated Fe(III) between the inoculated and the abiotic tubes was very small after 1 day. Nevertheless, in the following days the microbially active tubes accumulated much more Fe(III) than the abiotic tubes, providing clear evidence for microbial Fe(II) oxidation (Fig. S2A and B). Specifically, during the first four days of incubation, Fe(II) concentrations in the bacterial cultures and abiotic controls remain almost identical (Fig. S1A). Thereafter, minor variations appear not only between the two different setups (biotic vs. abiotic) but also between the individual gradient tubes of one setup reflected in the increasing error bars (Fig. S1A). From day 2 until the end of the experiment, Fe(III) concentrations in the microbially active tubes were approximately 33±6% higher than in the abiotic controls. Despite this, the Fe(II)/Fe(III) ratio remained similar between the bacterial cultures and abiotic controls throughout the experiment (Fig. 4D).

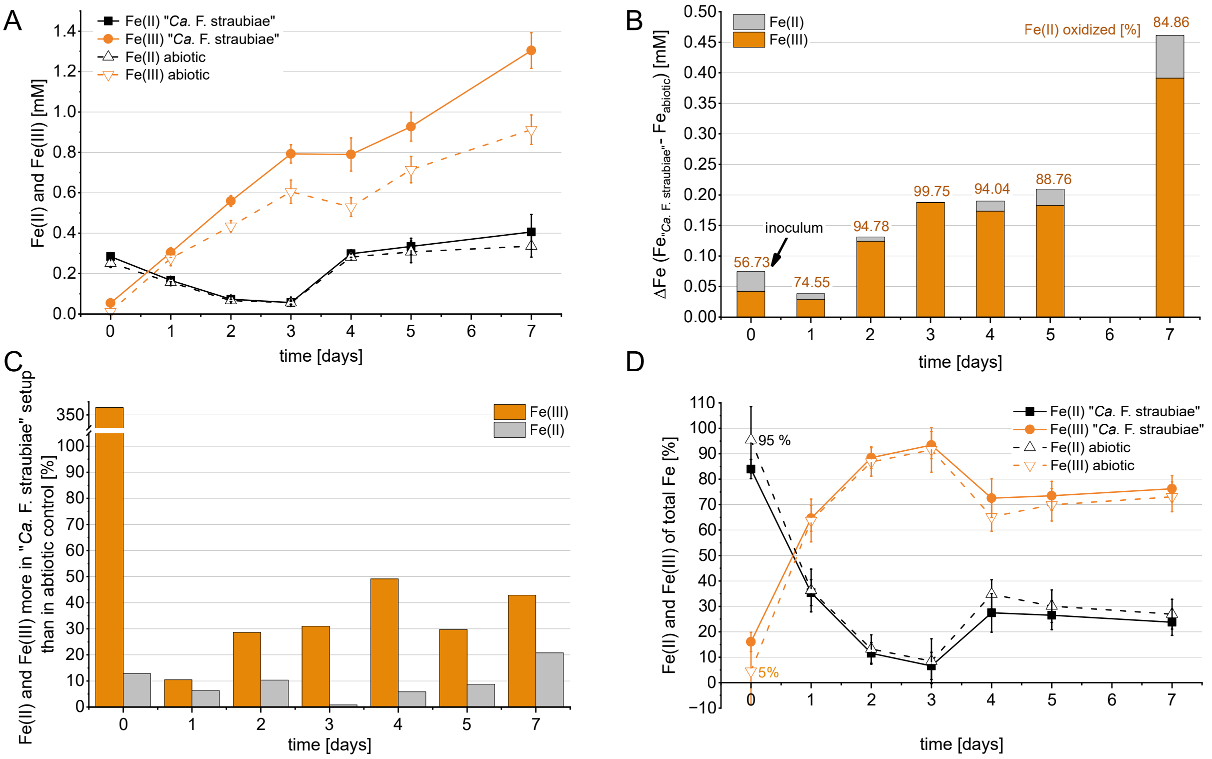

Figure S1: Abundance of Fe(II) and Fe(III) in "*Ca.* F. straubiae" cultures grown in agarose-stabilized Fe(II)-oxygen gradient tube vs abiotic controls. Samples were collected from a homogenized top layer. (A) Fe(II) and Fe(III) concentration in mM. Error bars correspond to biological triplicates. (B) Total difference in Fe concentration which is the sum of ΔFe(III) (orange) and ΔFe(II) (gray). (C) shows how much more Fe(III) and Fe(II) is present in the biotic setups vs the abiotic controls. ΔFe(III) (orange) and ΔFe(II) (gray) were normalized by the total amount of Fe(III) or Fe(II), respectively, of the abiotic controls. (D) Fe(II) and Fe(III) normalized to total Fe concentration in "*Ca.* F. straubiae" gradient tube cultures and abiotic setups, respectively. Error bars correspond to biological triplicates. Culture was grown under optimal conditions (pH 6.5, 25°C).

Fe(II) accumulates after 4 days of incubation

While Fe(II) was continuously released from the FeS plug into the top layer of the culture (rates in Fig. S2C concentrations in main text Fig.3A), Fe(II) oxidation changed significantly between days 3 and 4 (rates in Fig. 2B, concentrations in Fig. S1A). As a result, the Fe(II) dissolution process becomes more dominant over the Fe(II) oxidation, leading to an increase in Fe(II) concentration (accumulation) in the top layer (rates Fig. S2C; concentrations Fig. S1A).

Comparison of Fe(II) oxidation and growth rates

The geochemical change between days 3 and 4, as explained in section 'Text SI-2' was observed not only in the abiotic control but also in the inoculated setups and affected the growth of "*Ca.* F. straubiae", which slowed drastically before resuming after day 4. We speculate that this reduced growth rate is attributed to the same factors affecting the abiotic reaction which was limited diffusion of new substrates to the reaction site.

This sudden stop of the exponential growth phase after day 3 has also been also found for strain OYT1 grown under comparable conditions (Kato *et al.* 2015). This suggests that the dynamics of both systems, despite higher Fe concentrations in the strain OY1 setup, changes after 3 days of introducing oxygen to the system. However, an additional biological response may have played a role for this intermediate lag phase. The bacteria likely adapted to the changing environment, possibly by producing an oxygen reductase with a higher affinity for oxygen, as suggested by (Han *et al.* 2011).

Our cell quantification data revealed that cell growth and iron(II) oxidation rates are not proportional, i.e. while Fe(II) oxidation is similar over the first three days after inoculation, bacterial growth constantly increases (Fig. S2B and Fig. S2A). However, this is not surprising as the abiotic and the biological Fe(II) oxidation occur simultaneously.

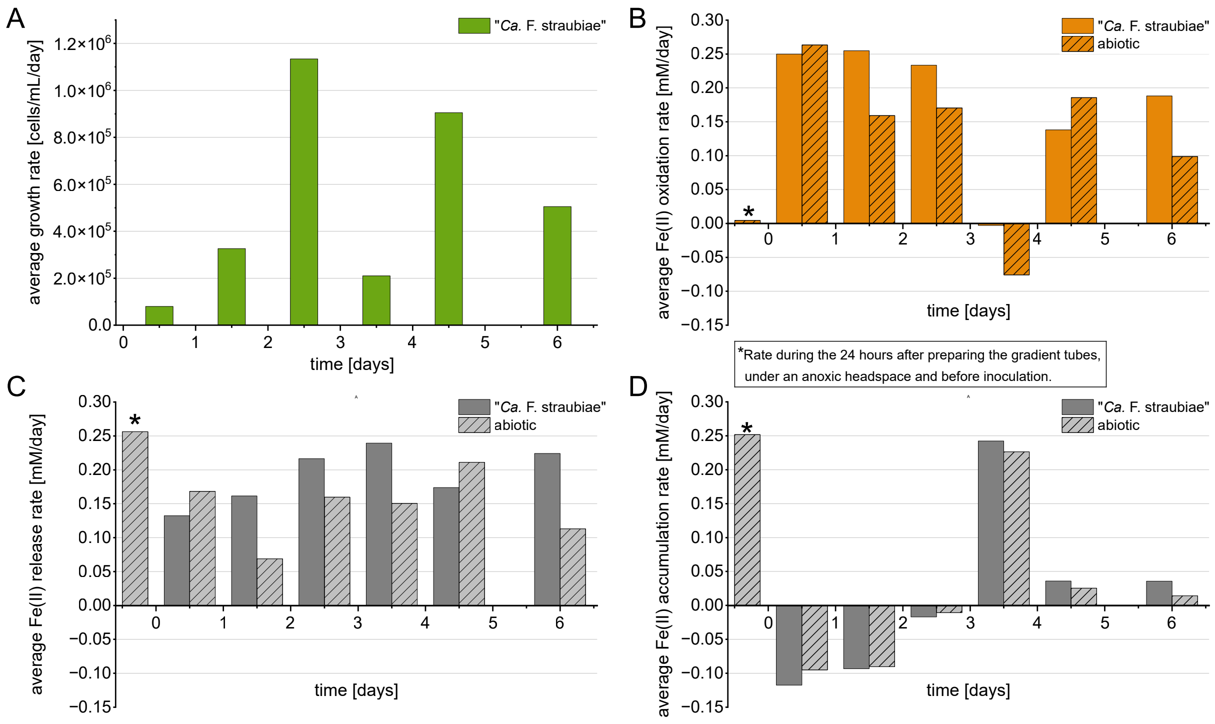

Figure S2: Rates of growth (A), Fe(II) oxidation (B), Fe(II) release from the FeS plug into the top layer (C), and Fe(II) decrease and accumulation in the top layer (D) for gradient tubes inoculated with "*Ca.* F. straubiae" and abiotic controls, respectively. Plot (D) is the difference of Fe(II) entering the system (as shown in C) and Fe(II) becoming oxidized (as shown in B). The negative values represent the decrease in Fe(II) concentration while the positive numbers represent the accumulation which starts three days after inoculation, and the timepoint when the Fe(II) oxidation becomes slower than the rate of Fe(II) entering the system. Errors in the raw data, corresponding to biological triplicates, are plotted in main text Fig. 3 and Fig. S2. For clarity, propagated errors are not displayed. The average rates are plotted in the middle of the two data points used to calculate the backward derivation. Thus, the rates represent the average rate of the system in between this two sampling points. Culture was grown under optimal conditions (pH 6.5, 25°C).

While the cells are initially in a lag phase, most of the Fe(II) oxidation can be attributed to an abiotic reaction. Consequently, the Fe(II) oxidation observed between days 0 and 1 was abiotic and thus no significant population increase was observed. After 1 day, the bacteria become active and oxidize Fe(II)

control (Fig. S2B). Further it is important to note that the biological and the abiotic reactions are competing—this means that in the biological setup there is likely less abiotic Fe(II) oxidation than in the abiotic setup. The proportion of Fe(II) oxidized by bacteria versus abiotic oxidation cannot be directly determined by our data. Given the higher growth rate between day 2 and 3, we speculate that biological contribution started to become more important around this time.

A caveat of this is that Fe(II) oxidation may not be directly coupled to increase in cell numbers, but the fixed carbon is rather stored intracellularly in a bioavailable from (carbon storage). In this scenario, biological Fe(II) oxidation is not proportional to population increase but to biomass increase, which was not measured.

Text SI-5: Calculation of headspace oxygen

The total oxygen availability in our tubes in µmol has been calculated using the ideal gas law (equation S1), assuming 21% O_2 in the air:

$$\frac{pV}{RT} = n \qquad \text{(S1)}$$

pressure (atmospheric pressure: 1 atm)
volume of the gas (headspace: 0.003865 L)
amount of substance (moles)
gas constant (0.0821 L atm/mol/K))
temperature (room temperature: 298.15 K)

By using these constants and a 3.865 mL headspace at room temperature the formula is:

$$\frac{1\ \mathrm{atm} \times 0.003865\ \mathrm{L}}{0.0821\ \mathrm{L} \cdot \frac{\mathrm{atm}}{(\mathrm{mol} \times \mathrm{K})} \times 298.15\ \mathrm{K}} = 0.000158\ \mathrm{mol} = 0.158\ \mathrm{mmol} \qquad \text{(S2)}$$

0.158 mmol was then multiplied by 21% oxygen in air, resulting in:

$$0.158\ \mathrm{mmol} \times 21\% = 0.033\ \mathrm{mmol} = 33.16\ \mathrm{\mu mol} \qquad \text{(S3)}$$

Protocol SI-1: Original protocol for FeS synthesis by AG Kappler

FACULTY OF SCIENCE
Department Geoscience
Geomicrobiology
Prof. Kappler

EBERHARD KARLS
UNIVERSITÄT
TÜBINGEN

FeS synthesis

Valid from: August 2016
Author: Tina Gauger
Modified from: W. Ruschmeier and Stefanie Becker

Preparation of FeS:

Materials:

- Gloves and safety goggles
- Scale
- Water bath
- Plastic spatula and tweezer
- 1 × Erlenmeyer flask (250ml)
- 1 × 0.5 L Schott bottle, butyl stopper and a needle
- Water and small container for rinsing of the Na_2S crystals
- Paper towel to dry the Na_2S crystals
- Little stabile plastic bag to weigh Na_2S crystals
- Hammer and towel (or other cloth to underlay)
- Tubing with clamp, syringe and bucket for washing procedure

Chemicals:

- $Na_2S \times 9H_2O$ (39.6 g/L)
- $FeSO_4 \times 7H_2O$ (46.2 g/L)
- 3 x 1 L 60°C warm dest. or milliQ water

Procedure:

- Heat up the 1 L water to 60°C in the waterbath, leave the lid open to let the O_2 outgasing
- **Work under the fume hood with gloves and safety goggles!**
- Have a scale under the fume hood
- Scale 19.8 g Na_2S
- Fill Na_2S in a the Schott bottle and add 100 ml warm water
- Weigh 23.1 g $FeSO_4$ in the Erlenmeyer flask and add 100 ml warm water
- Dissolve both solutions through gently mixing
- **Fairly quickly**, add the $FeSO_4$ to the Na_2S, you should observe a color change to black
- Fill **gently** the bottle with water until it overflows
- Stick a needle though the stopper. Close the bottle with the needle in the stopper, and avoid having any air in the headspace. Take out the needle when the bottle is closed!
- Leave it overnight
- When the FeS settled down start washing
- Heat up 2 L water in a water bath
- Exchange the water layer over the FeS with 2 L water until it looks clear
- Use a bottle of water as shown in the picture which is placed higher than the FeS bottle

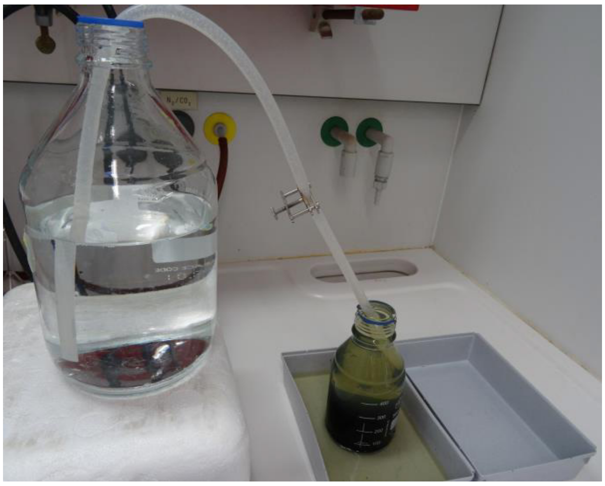

- Put the FeS bottle in a bucket to collect the overflow
- Place one end of the tubing in the warm water and pull with a syringe at the other end
- Adjust the flow rate with the clamp
- Let the water flow into the FeS bottle. Caution: tubing should not go into the FeS layer as it will mix the two layers
- When the water over the FeS looks clean, fill the bottle with water completely and close it with a stopper

After washing, the pH of the FeS solution should be close to neutrality. The FeS layer is normally quite hydrous and can be pipetted with a standard 10 or 5-ml pipette.
After removing FeS for use, it is important to top the bottle up with
dH_2O and keep it stoppered tightly to limit the influx of oxygen. With
limited oxygen exposure, the FeS can be maintained at room temperature. Do not stir or shake the sediment.
FeS does age and becomes "weaker", releases less Fe(II) when used for gradient tubes. Each batch of FeS is slightly different. **If the FeS smells strongly of sulfide following the washing steps and when it has a strongly alkaline pH, it should be discarded.**

In the presence of acid, sodium sulfide will immediately release hydrogen sulfide, an extremely toxic gas. Preparing FeS in a chemical fume hood is strongly recommended.

Protocol SI-2: Original protocol for gradient tube preparation by AG Kappler

FACULTY OF SCIENCE
Department Geoscience
Geomicrobiology
Prof. Kappler

EBERHARD KARLS
UNIVERSITÄT
TÜBINGEN

Gradient tubes

Valid from: September 2016
Author: Tina Gauger, Juanjuan, Marie Muehe
Modified from: David Emerson, Eva Winkler, Eva Wartha, Sarah Richter
Adapted to "*Ca.* F. straubiae" cultivation by Stefanie Becker (Sep. 2025)

Gradient tubes for cultivation of microorganisms

Setup for microaerophilic Fe(II)-oxidizers:

A natural, diluted or cultured sample is inoculated into a semi-liquid medium that exhibits opposing gradients of oxygen (electron acceptor) and Fe(II) (electron source). The microorganisms will grow at a height in the tube which offers the most favorable growth conditions, i.e. optimal concentrations of Fe(II) and O_2 to compete successfully with chemical Fe(II) oxidation.

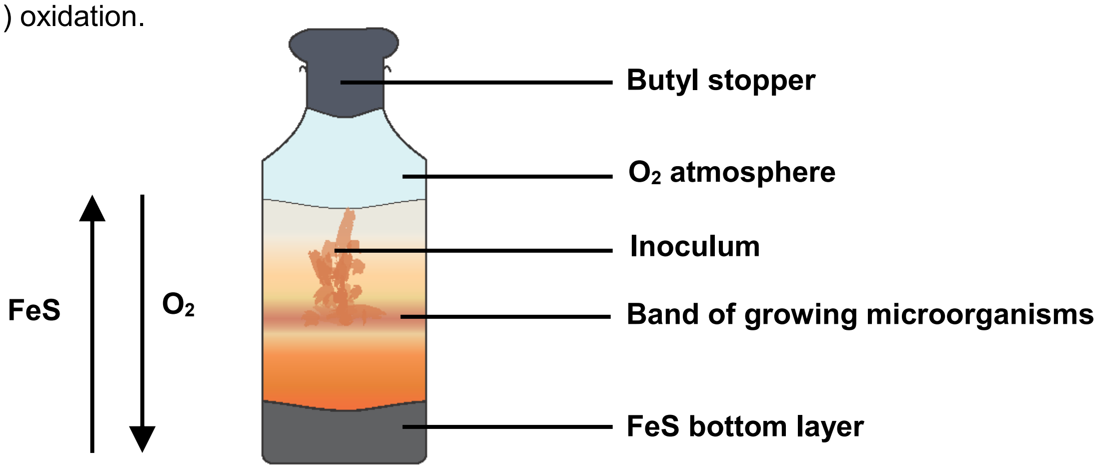

Preparation of solutions:

1) FeS

We all use the FeS together, so one person makes it and we all use it up. That means we need to trust each other to work cleanly and to always prepare and treat the FeS to one general protocol!
→ see detailed FeS manual

If you need FeS, take some with a long plastic pipette and replace the volume by adding VE water, do not stir or shake the sediment

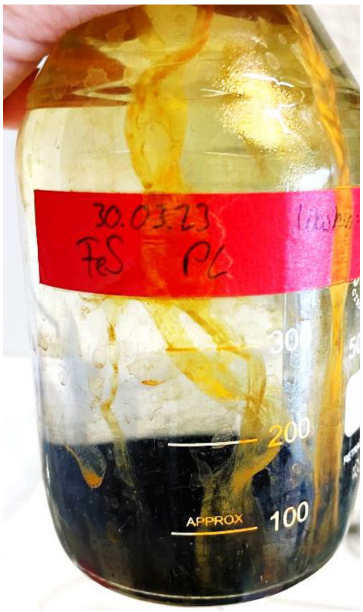

2) **1 MWMM-Medium (according to Emerson & Floyd 2005)**

- Dissolve each of these salts one after the other in 900 mL MQ water
  - 1 g ammonium chloride (NH_4Cl)
  - 0.2 g magnesium sulfate heptahydrate ($MgSO_4$ x 7 H_2O)
  - 0.1 g calcium chloride dihydrate ($CaCl_2$ x 2 H_2O)
  - 0.05 dipotassium hydrogen phosphate (K_2HPO4)
- Fill up to 1 liter with Millipore water and stir until dissolved
- Autoclave
- This medium is low in sulfate in order to minimized the growth of sulfate-reducers

Preparation of tubes and layers (1 batch = 22-24 tubes):

1) **Preparations**
   - Autoclave clean butyl stoppers
   - Autoclave and dry gradient tubes with lids (always use the green lids) in 60°C oven

2) **Bottom layer**
   - Weigh 0.1 g high melt agarose in headspace vial (c_{end} = 1%)
   - Add 5 mL of MWMM (oxic)
   - Add 5 mL of FeS (sensible to oxidation!)
   - Flush with N_2 and close with stopper and crimp
   - You need 2 headspace vials for 1 batch
   - Autoclave
   - Keep at 65 °C until use or heat to 90 °C in a water bath just before use

3) **Top layer**
   - Weigh 0.15 g low melt agarose in a 250 mL SCHOTT bottle
   - Weigh 0.084 g sodium hydrogen carbonate ($NaHCO_3$) in another 250mL SCHOTT bottle (10 mM buffer)
   - Add 100 mL MWMM (oxic) to $NaHCO_3$ (mix gently)
   - Measure the pH and adjust to 6.5 by adding 1 M HCl
   - Add the buffered MWMM to the low melt agarose (It is important to have the buffer in the medium, otherwise the agar will not crosslink properly.)
   - Flush the headspace with N_2/CO_2 for 3-5 min, then close the bottle with a butyl stopper
   - Autoclave
   - After autoclaving keep at 50-65°C in a water bath

Making the tubes:

- For filling the tubes work in the fume hood, your Bunsen burner is the center of your setup, your stoppers, ice box and pipette tips should be located around it

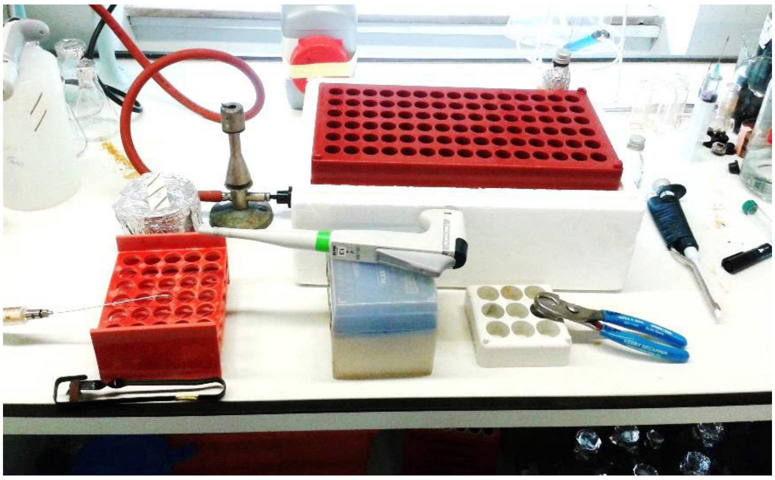

- Place 22-24 tubes on the cooled rack (in ice box)
- Turn on the Bunsen burner and screw open the tubes carefully
- Take 1 bottom layer vial out of the water bath, shake it thoroughly
- Open the vial carefully with the decrimper (close the window of the fume hood as much as you can as the bottom layer might spill when opened)
- Fill 760 µL of the bottom layer in the first 11-12 vials using sterile pipette tips
- The bottom layers should harden at once
- Repeat these steps with 2nd bottom layer vial
- Cool down top layer until it is lukewarm (hand-hot), otherwise your vitamins might be destroyed, then
  - Add 100 µL 7 vitamine solution (c_{end} = 1 mL/L)
  - Add 100 µL trace element solution SL10 (c_{end} = 1 mL/L)
  Modification for "*Candidatus* Ferrigenium Straubiae":
  - Extra Supplements, final concentration: 10 µM Na_2SeO_3, 10 µM Na_2MoO_4, 0.1 µM $NiCl_2$, 0.1 µM $CuCl_2$, 0.1 µM AsO_3, 15 nM NH_4VO_3. (Stock solution containing all the additives maybe be prepared as 1:200, this is what I used.)
  - Selenite-tungstate, final concentration: 6 µg Na_2SeO_3x5H_2O and 8 µg Na_2WO_4x2H_2O. (Standard stock solution is 1:1000)
- Mix gently
- Check that bottom layer is solidified
- Using the 5 mL pipette, add 3.875 mL top layer carefully to each tube (let it slide down the wall, then the bottom layer won't start floating)
- Flush each tube with N_2/CO_2
- Close the tube with a stopper
- Store all tubes in drawer

Inoculating the tubes *(work next to flame or clean bench!)*:

- You inoculate the gradient tubes at the next day after preparation
- Inoculate the tubes from previous gradient tube. Have it ready.
- Open the fresh tube and place the stopper on a glass cylinder which is tuned up-site down. The bottom of the cylinder functions as your table which you previously sterilized by ethanol and flame.
- Open the old tube and carefully take up 40 µL sample with a pipette from the oxidized band/growth area. Ideally use tips with filter.
- Now you go to the fresh tube and inject the sample into the medium just above the FeS.
- While injecting the 40 µL sample, take out the pipette at the same time to spread the inoculum from bottom to top of the top layer.
- Flame off stopper and close the tube.

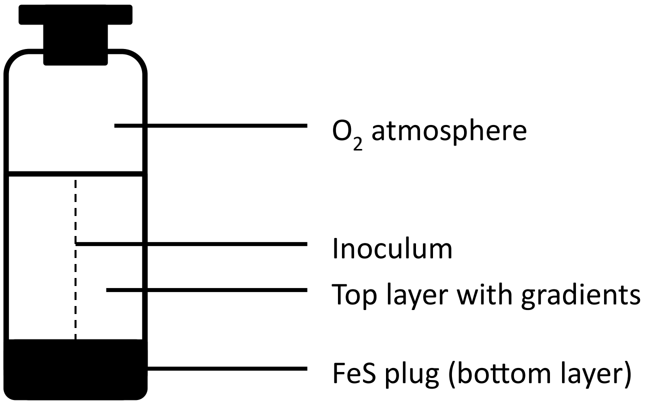

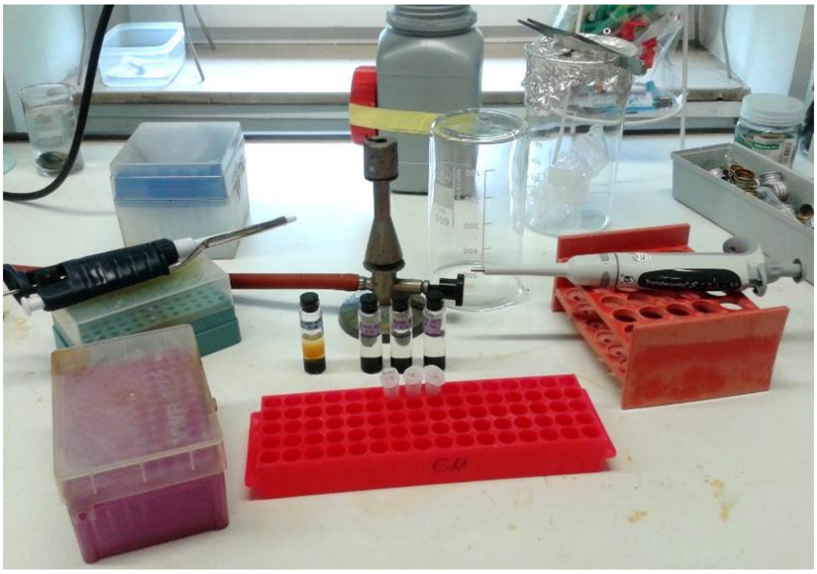

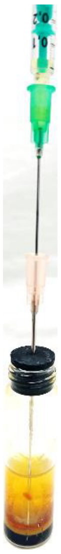

Two needle sampling *(to check viability while avoiding contamination)*:

- Sterilize the stopper by ethanol and flame.
- Pull a Ø 1.2 × 40 mm needle through the stopper
- Insert the thinner needle Ø 0.8 × 120 mm though the wider needle that goes through the stopper (see figure on the left)
- Take a sample using a 1 mL syringe

Figure SI-3: Sanger sequencing reads for the identification of "*Ca.* F. straubiae"

(A : 1/4)

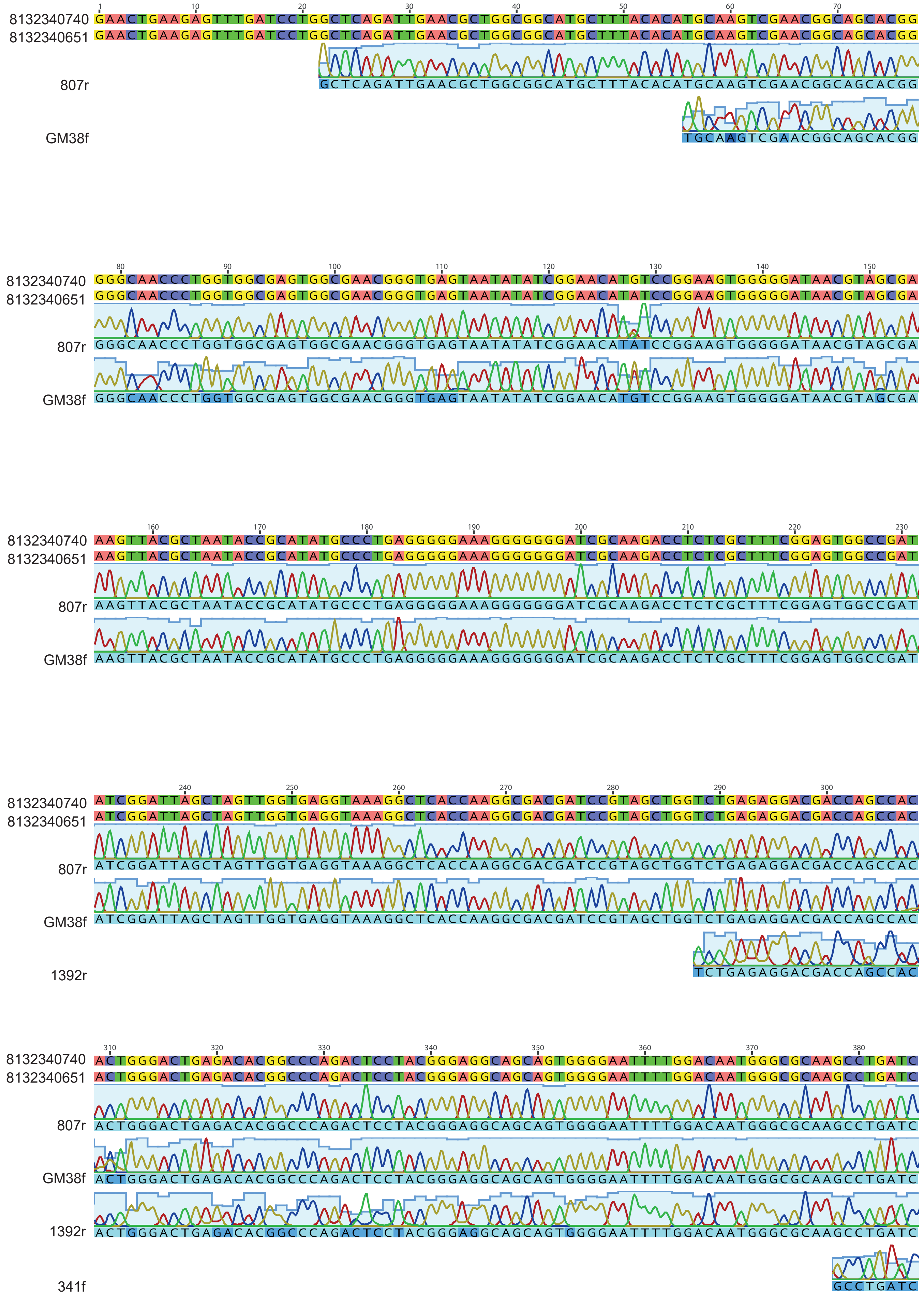

(A : 2/4)

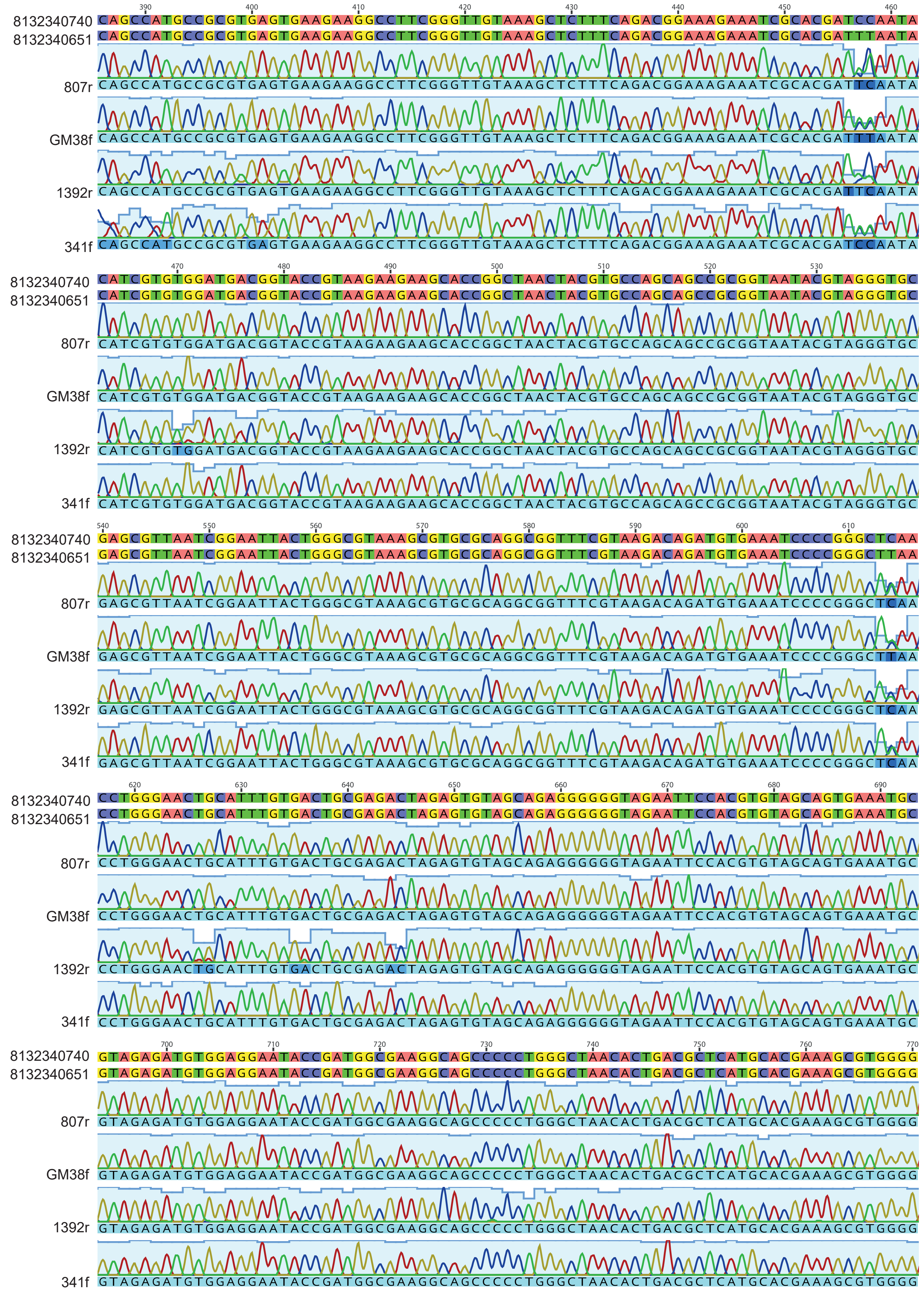

8132340740
8132340651
807r
GM38f
1392r
341f
390
400
410
420
430
440
450
460
470
480
490
500
510
520
530
540
550
560
570
580
590
600
610
620
630
640
650
660
670
680
690
700
710
720
730
740
750
760
770

(A : 3/4)

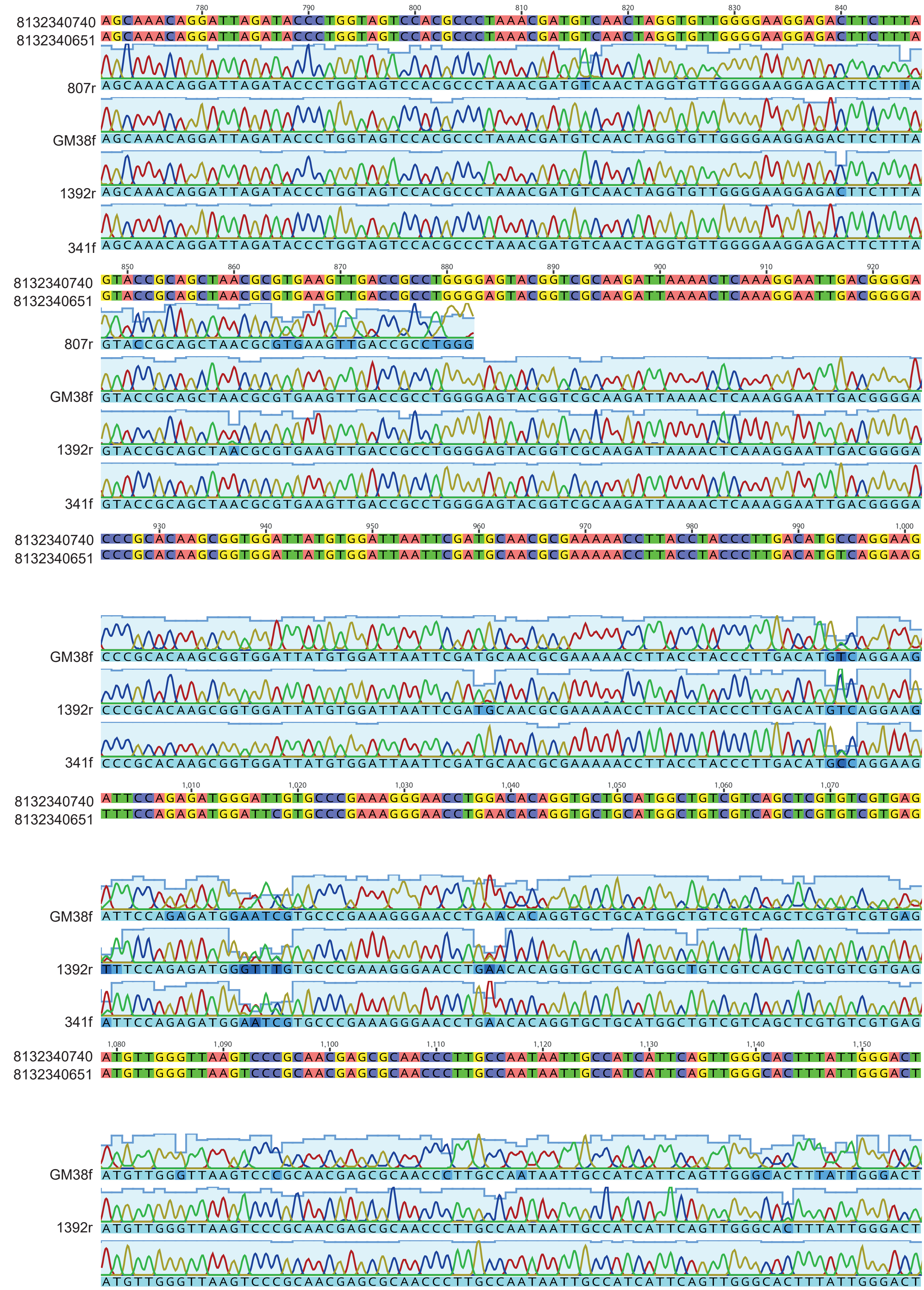
8132340740
8132340651
807r
GM38f
1392r
341f
780
790
800
810
820
830
840
850
860
870
880
890
900
910
920
930
940
950
960
970
980
990
1,000
1,010
1,020
1,030
1,040
1,050
1,060
1,070
1,080
1,090
1,100
1,110
1,120
1,130
1,140
1,150

(A : 4/4)

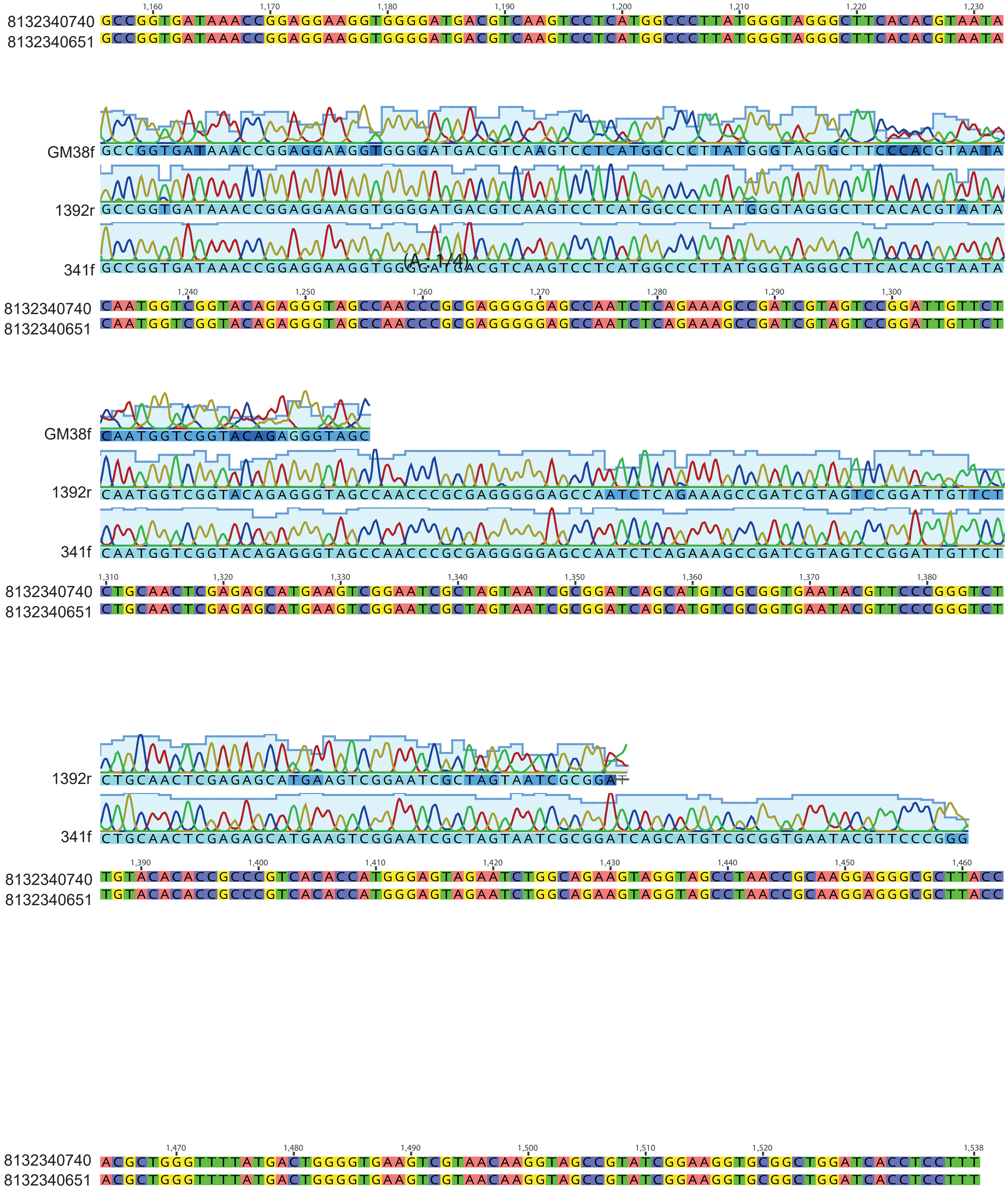

8132340740 GCCGGTGATAAACCGGAGGAAGGTGGGGATGACGTCAAGTCCTCATGGCCCTTATGGGTAGGGCTTCACACGTAATA
8132340651 GCCGGTGATAAACCGGAGGAAGGTGGGGATGACGTCAAGTCCTCATGGCCCTTATGGGTAGGGCTTCACACGTAATA
GM38f GCCGGTGATAAACCGGAGGAAGGTGGGGATGACGTCAAGTCCTCATGGCCCTTATGGGTAGGGCTTCCCACGTAATA
1392r GCCGGTGATAAACCGGAGGAAGGTGGGGATGACGTCAAGTCCTCATGGCCCTTATGGGTAGGGCTTCACACGTAATA
341f GCCGGTGATAAACCGGAGGAAGGTGGGGATGACGTCAAGTCCTCATGGCCCTTATGGGTAGGGCTTCACACGTAATA
(A : 1/4)
8132340740 CAATGGTCGGTACAGAGGGTAGCCAACCCGCGAGGGGGAGCCAATCTCAGAAAGCCGATCGTAGTCCGGATTGTTCT
8132340651 CAATGGTCGGTACAGAGGGTAGCCAACCCGCGAGGGGGAGCCAATCTCAGAAAGCCGATCGTAGTCCGGATTGTTCT
GM38f CAATGGTCGGTACAGAGGGTAGC
1392r CAATGGTCGGTACAGAGGGTAGCCAACCCGCGAGGGGGAGCCAATCTCAGAAAGCCGATCGTAGTCCGGATTGTTCT
341f CAATGGTCGGTACAGAGGGTAGCCAACCCGCGAGGGGGAGCCAATCTCAGAAAGCCGATCGTAGTCCGGATTGTTCT
8132340740 CTGCAACTCGAGAGCATGAAGTCGGAATCGCTAGTAATCGCGGATCAGCATGTCGCGGTGAATACGTTCCCGGGTCT
8132340651 CTGCAACTCGAGAGCATGAAGTCGGAATCGCTAGTAATCGCGGATCAGCATGTCGCGGTGAATACGTTCCCGGGTCT
1392r CTGCAACTCGAGAGCATGAAGTCGGAATCGCTAGTAATCGCGGA
341f CTGCAACTCGAGAGCATGAAGTCGGAATCGCTAGTAATCGCGGATCAGCATGTCGCGGTGAATACGTTCCCGGG
8132340740 TGTACACACCGCCCGTCACACCATGGGAGTAGAATCTGGCAGAAGTAGGTAGCCTAACCGCAAGGAGGGCGCTTACC
8132340651 TGTACACACCGCCCGTCACACCATGGGAGTAGAATCTGGCAGAAGTAGGTAGCCTAACCGCAAGGAGGGCGCTTACC
8132340740 ACGCTGGGTTTTATGACTGGGGTGAAGTCGTAACAAGGTAGCCGTATCGGAAGGTGCGGCTGGATCACCTCCTTT
8132340651 ACGCTGGGTTTTATGACTGGGGTGAAGTCGTAACAAGGTAGCCGTATCGGAAGGTGCGGCTGGATCACCTCCTTT

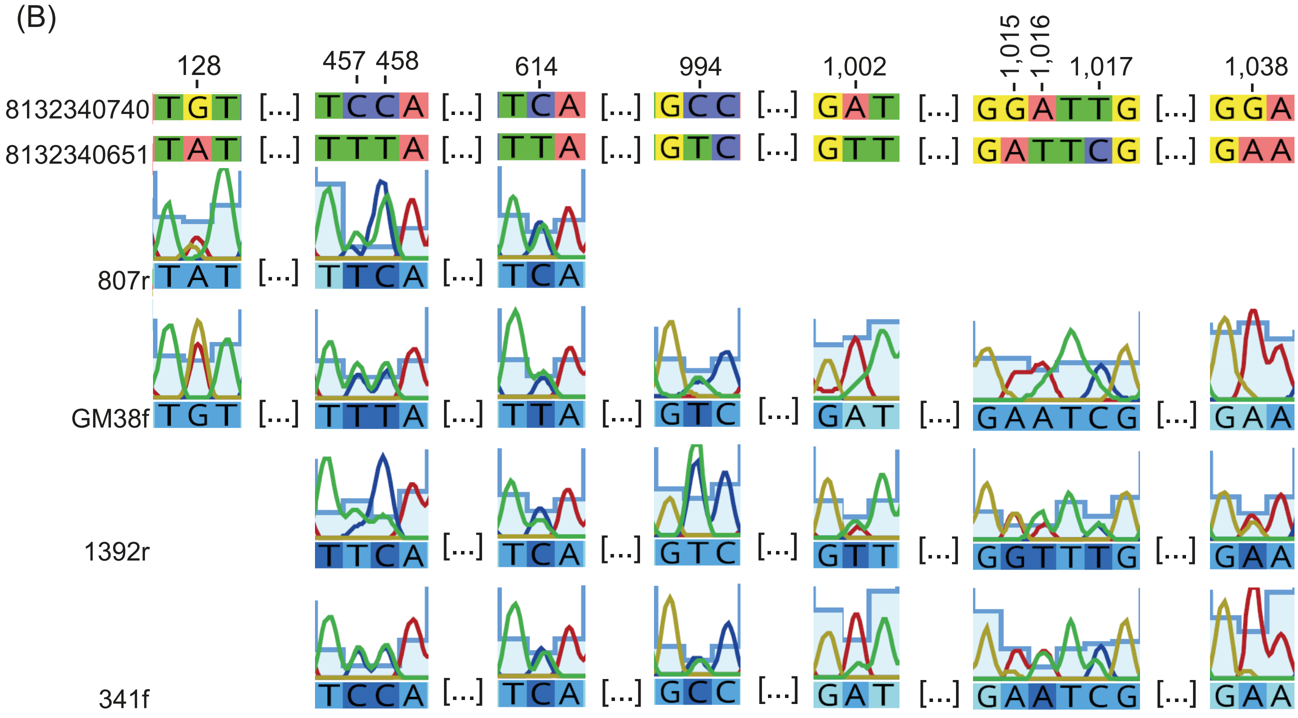

Figure S3: (A 1-4) Sanger sequencing reads corresponding to primers 807R, GM38F, 1392R, and 341F were mapped to 16S rRNA genes (sequences 813240740 and 813240651) of "*Ca*. F. straubiae" strain KS. These genes were downloaded from IMG (Chen *et al.* 2023) and correspond to the newly derived genome of the pure strain KS (Taxon ID: 8132338324). Sequences at the beginning and end of the reads that were of low quality were trimmed. Reference primers used: 807r (Lane *et al.* 1985); GM38f (Muyzer *et al.* 1995); 1392r (Lane *et al.* 1985)and 341f (Klindworth *et al.* 2013). (B) Close up of all differences in the two 16S rRNA gene copies.

Figure SI-4: Contamination screening by fluorescence microscopy

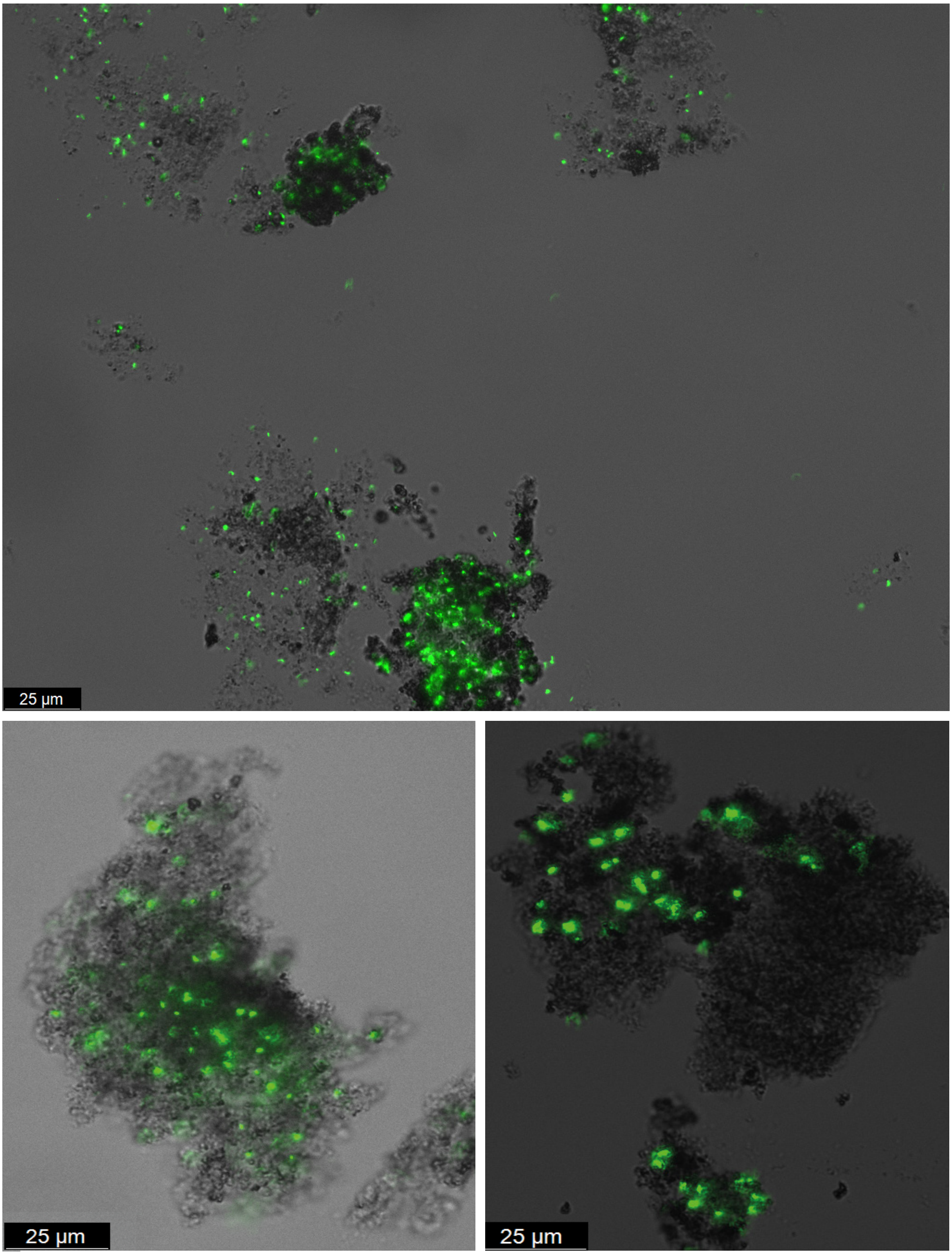

Figure S4: Fluorescence micrographs (all same condition) showing "*Ca.* F. straubiae" strain KS stained with SYTOX™ Green associated with Fe(III) minerals. The sample got a heat treatment of 5 min at 70°C prior to staining. Culture grew in an agarose-stabilized Fe(II)-O_2 gradient tube and steamed from the oxidized zone just above the FeS plug.

Software output SI-1: QUAST (Gurevich *et al.* 2013) results from comparing the metagenome-derived genome of strain KS (Huang *et al.* 2021, 2022) and the genome derived from the pure culture of strain KS (this study).

Report

	Cfs_polished_genome
# contigs (>= 0 bp)	1
# contigs (>= 1000 bp)	1
# contigs (>= 5000 bp)	1
# contigs (>= 10000 bp)	1
# contigs (>= 25000 bp)	1
# contigs (>= 50000 bp)	1
Total length (>= 0 bp)	2666173
Total length (>= 1000 bp)	2666173
Total length (>= 5000 bp)	2666173
Total length (>= 10000 bp)	2666173
Total length (>= 25000 bp)	2666173
Total length (>= 50000 bp)	2666173
# contigs	1
Largest contig	2666173
Total length	2666173
Reference length	2659708
GC (%)	60.24
Reference GC (%)	60.26
N50	2666173
NG50	2666173
N90	2666173
NG90	2666173
auN	2666173.0
auNG	2672653.7
L50	1
LG50	1
L90	1
LG90	1
# misassemblies	11
# misassembled contigs	1
Misassembled contigs length	2666173
# local misassemblies	4
# scaffold gap ext. mis.	0
# scaffold gap loc. mis.	0
# unaligned mis. contigs	0
# unaligned contigs	0 + 1 part
Unaligned length	4774
Genome fraction (%)	99.998
Duplication ratio	1.002
# N's per 100 kbp	0.00
# mismatches per 100 kbp	28.46
# indels per 100 kbp	2.07
Largest alignment	1120297
Total aligned length	2660018
NA50	599139
NGA50	599139
NA90	186567
NGA90	186567
auNA	691337.2
auNGA	693017.6
LA50	2
LGA50	2
LA90	5
LGA90	5

Misassemblies report

	Cfs_polished_genome
# misassemblies	11
# contig misassemblies	11
# c. relocations	5
# c. translocations	6
# c. inversions	0
# scaffold misassemblies	0
# s. relocations	0
# s. translocations	0
# s. inversions	0
# misassembled contigs	1
Misassembled contigs length	2666173
# local misassemblies	4
# scaffold gap ext. mis.	0
# scaffold gap loc. mis.	0
# unaligned mis. contigs	0
# mismatches	757
# indels	55
# indels (<= 5 bp)	35
# indels (> 5 bp)	20
Indels length	1348

Unaligned report

	Cfs_polished_genome
# fully unaligned contigs	0
Fully unaligned length	0
# partially unaligned contigs	1
Partially unaligned length	4774
# N's	0

All statistics are based on contigs of size >= 500 bp, unless otherwise noted (e.g., "# contigs (>= 0 bp)" and "Total length (>= 0 bp)" include all contigs).

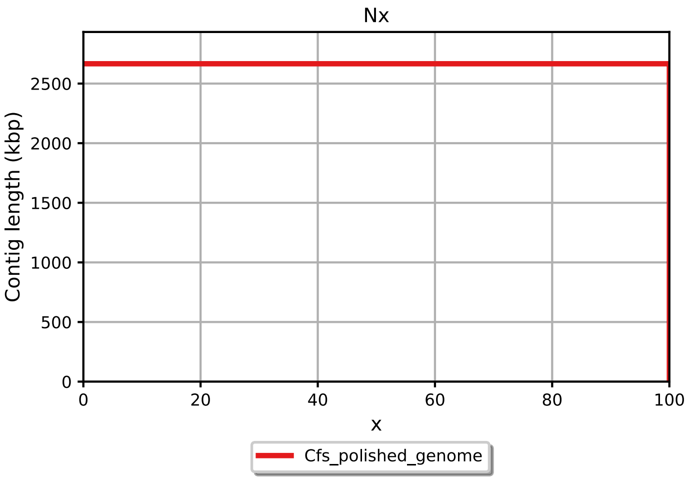
Nx
Contig length (kbp)
0
500
1000
1500
2000
2500
0
20
40
60
80
100
x
Cfs_polished_genome

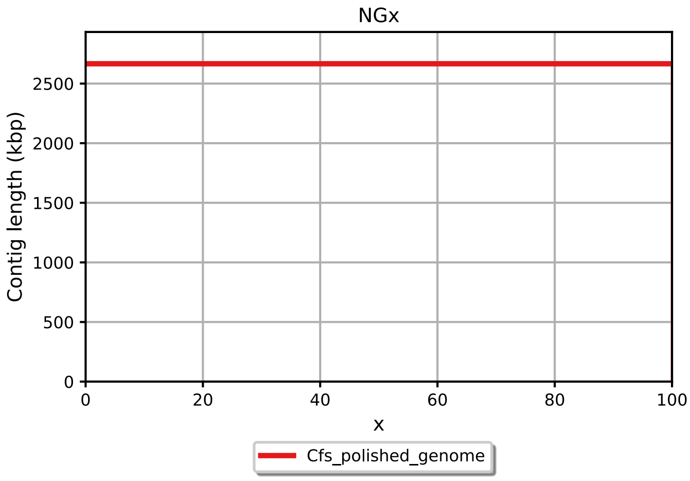
NGx
Contig length (kbp)
0
500
1000
1500
2000
2500
0
20
40
60
80
100
x
Cfs_polished_genome

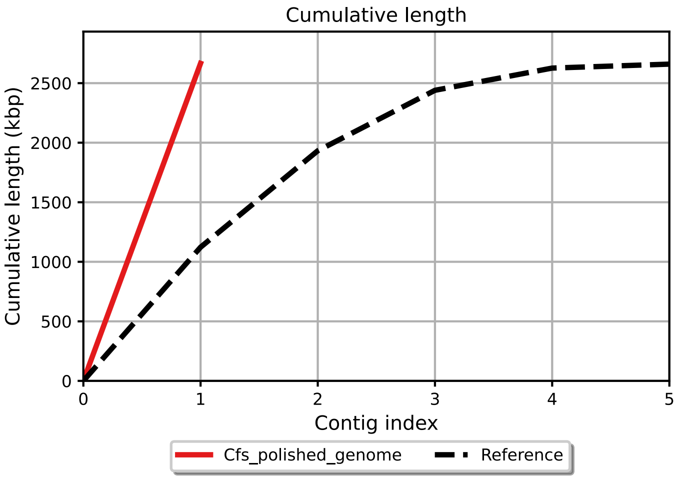
Cumulative length
Cumulative length (kbp)
0
500
1000
1500
2000
2500
0
1
2
3
4
5
Contig index
Cfs_polished_genome
Reference

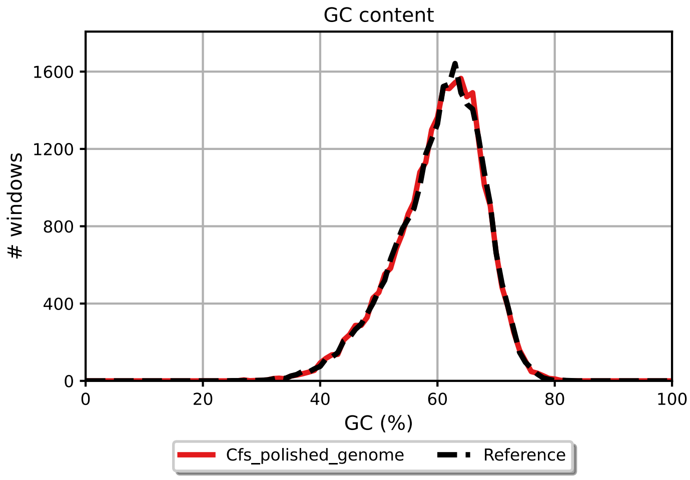
GC content
windows
0
400
800
1200
1600
0
20
40
60
80
100
GC (%)
Cfs_polished_genome
Reference

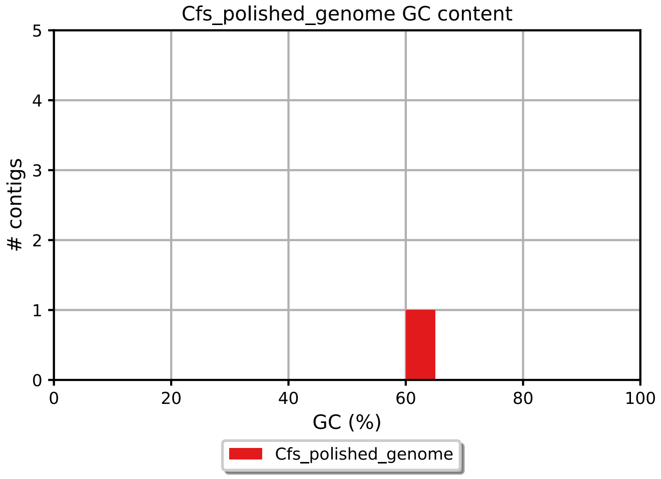
Cfs_polished_genome GC content
contigs
0
1
2
3
4
5
0
20
40
60
80
100
GC (%)
Cfs_polished_genome

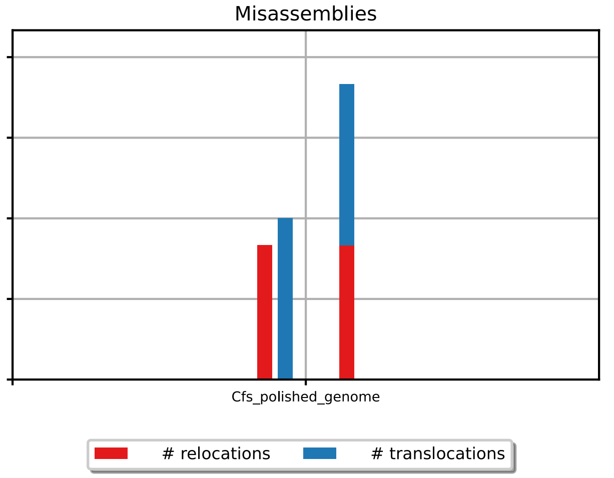
Misassemblies
0
3
6
9
12
Cfs_polished_genome
relocations
translocations

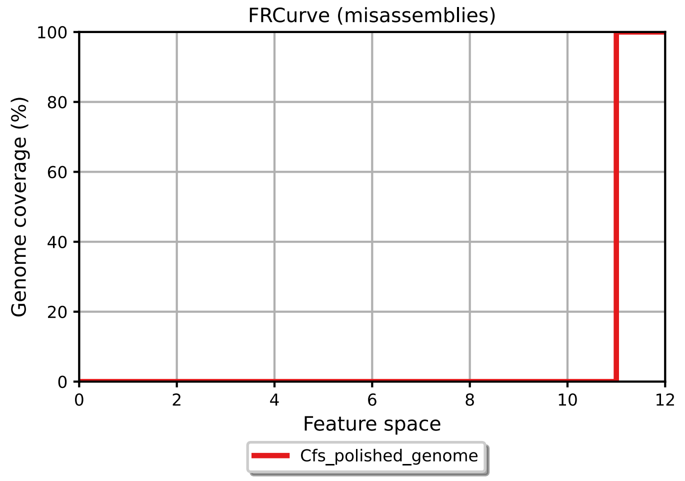
FRCurve (misassemblies)
Genome coverage (%)
Feature space
Cfs_polished_genome

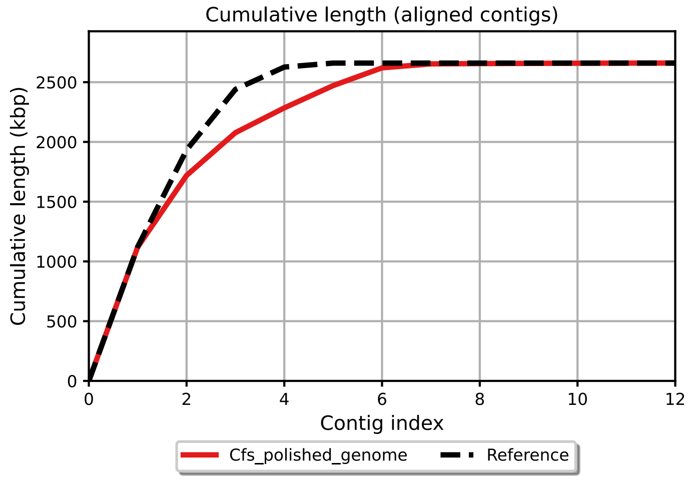
Cumulative length (aligned contigs)
Cumulative length (kbp)
Contig index
Cfs_polished_genome
Reference

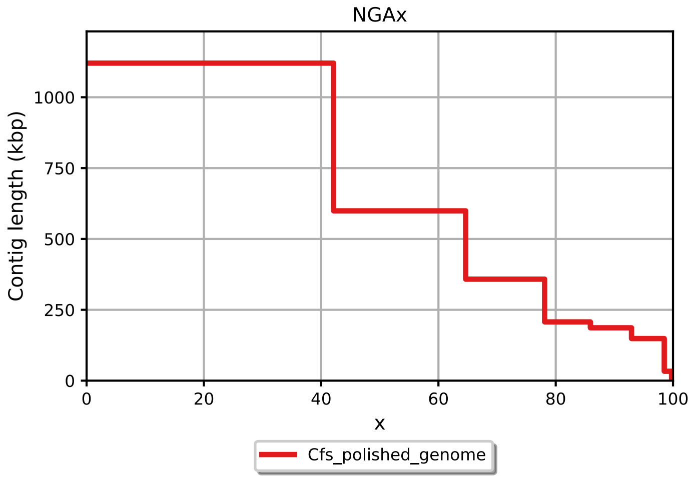
NGAx
Contig length (kbp)
x
Cfs_polished_genome

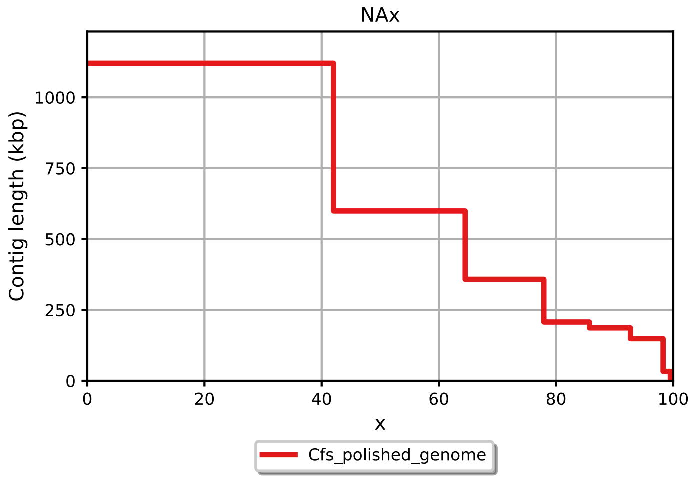
NAx
Contig length (kbp)
x
Cfs_polished_genome

Software output SI-2: BUSCO v5.8.3 (Manni *et al.* 2021) results from completeness and contamination test.

Results from 116 bacterial markers:

BUSCO version is: 5.8.3
The lineage dataset is: bacteria_odb12 (Creation date: 2025-05-14, number of genomes: 3130, number of BUSCOs: 116)
Summarized benchmarking in BUSCO notation for file /home/daniel/DATA/25-05-27_QTTUU_Stefanie/results_unicycler/Medaka/Cfs_polished_genome.fa
BUSCO was run in mode: prok_genome_prod
Gene predictor used: prodigal

```
        ***** Results: *****

        C:100.0%[S:100.0%,D:0.0%],F:0.0%,M:0.0%,n:116
        116     Complete BUSCOs (C)
        116     Complete and single-copy BUSCOs (S)
        0       Complete and duplicated BUSCOs (D)
        0       Fragmented BUSCOs (F)
        0       Missing BUSCOs (M)
        116     Total BUSCO groups searched

Assembly Statistics:
        1       Number of scaffolds
        1       Number of contigs
        2666173         Total length
        0.000%  Percent gaps
        2 MB    Scaffold N50
        2 MB    Contigs N50

Dependencies and versions:
        hmmsearch: 3.4
        bbtools: None
        prodigal: 2.6.3
        python: sys.version_info(major=3, minor=12, micro=10, releaselevel='final', serial=0)
        busco: 5.8.3
```

<u>Results from 667 *Nitrosomonas* markers:</u>

BUSCO version is: 5.8.3
The lineage dataset is: nitrosomonadales_odb12 (Creation date: 2025-05-14, number of genomes: 65, number of BUSCOs: 667)
Summarized benchmarking in BUSCO notation for file /home/daniel/DATA/25-05-27_QTTUU_Stefanie/results_unicycler/Medaka/Cfs_polished_genome.fa
BUSCO was run in mode: prok_genome_prod
Gene predictor used: prodigal

```
        ***** Results: *****

        C:97.6%[S:96.6%,D:1.0%],F:0.7%,M:1.6%,n:667
        651     Complete BUSCOs (C)
        644     Complete and single-copy BUSCOs (S)
        7       Complete and duplicated BUSCOs (D)
        5       Fragmented BUSCOs (F)
        11      Missing BUSCOs (M)
        667     Total BUSCO groups searched

Assembly Statistics:
        1       Number of scaffolds
        1       Number of contigs
        2666173         Total length
        0.000%  Percent gaps
        2 MB    Scaffold N50
        2 MB    Contigs N50

Dependencies and versions:
        hmmsearch: 3.4
        bbtools: None
        prodigal: 2.6.3
        python: sys.version_info(major=3, minor=12, micro=10, releaselevel='final', serial=0)
        busco: 5.8.3
```
